# Precise, predictable genome integrations by deep-learning-assisted design of microhomology-based templates

**DOI:** 10.1038/s41587-025-02771-0

**Published:** 2025-08-12

**Authors:** Thomas Naert, Taiyo Yamamoto, Shuting Han, Ruth Röck, Melanie Horn, Philipp Bethge, Nikita Vladimirov, Fabian F. Voigt, Joana Figueiro-Silva, Ruxandra Bachmann-Gagescu, Kris Vleminckx, Fritjof Helmchen, Soeren S. Lienkamp

**Affiliations:** 1 Institute of Anatomy, University of Zurich, Zurich, Switzerland; 2 Department of Biomedical Molecular Biology, Ghent University, Ghent, Belgium; 3 PhD Program in Molecular Life Sciences, Life Science Zurich Graduate School, Zurich, Switzerland; 4 Brain Research Institute, University of Zurich, Zurich, Switzerland; 5 Neuroscience Center Zurich, University of Zurich, Zurich, Switzerland; 6 University Research Priority Program (URPP) Adaptive Brain Circuits in Development and Learning (AdaBD), University of Zurich, Zurich, Switzerland; 7 Center for Microscopy and Image Analysis (ZMB), University of Zurich, Zurich, Switzerland; 8 Department of Molecular and Cellular Biology, Harvard University, Cambridge, MA USA; 9 Institute of Medical Genetics, University of Zurich, Zurich, Switzerland; 10 Department of Molecular Life Sciences, University of Zurich, Zurich, Switzerland; 11 Zurich Kidney Center, Zurich, Switzerland; 12 Department of Health Sciences and Technology, ETH Zurich, Zurich, Switzerland

**Keywords:** Genetic engineering, Genetic engineering, Mutagenesis, Double-strand DNA breaks, Xenopus

## Abstract

Precise CRISPR-based DNA integration and editing remain challenging, largely because of insufficient control of the repair process. We find that repair at the genome–cargo interface is predictable by deep learning models and adheres to sequence-context-specific rules. On the basis of in silico predictions, we devised a strategy of base-pair tandem repeat repair arms matching microhomologies at double-strand breaks. These repeat homology arms promote frame-retentive cassette integration and reduce deletions both at the target site and within the transgene. We demonstrate precise integrations at 32 loci in HEK293T cells. Germline-transmissible transgene integration and endogenous protein tagging in *Xenopus* and adult mouse brains demonstrated precise integration during early embryonic cleavage and in nondividing, differentiated cells. Optimized repair arms also facilitated small edits for scarless single-nucleotide or double-nucleotide changes using oligonucleotide templates in vitro and in vivo. We provide the design tool Pythia to facilitate precise genomic integration and editing for experimental and therapeutic purposes for a wide range of target cell types and applications.

## Abstract (web summary)

Genomic integration of DNA templates is made more precise through microhomology-focused design.

## Main

The precise and targeted integration of transgenes using CRISPR–Cas technology holds great promise for applications in biotechnology and gene therapy^1^. However, it is paramount that genomic integrity is maintained to avoid unintended side effects and the integration technique is suitable for targeting the intended cell types^2,3^. Typically, CRISPR–Cas-mediated integration relies on homology-directed repair (HDR), which necessitates large homology arms and is only active in proliferating cells, or on nonhomologous end joining (NHEJ), microhomology (µH)-mediated end joining (MMEJ) or single-strand annealing^4^. However, NHEJ and MMEJ may result in unintended genomic alterations at transgene–genome borders, including deletions within the surrounding genome or transgene, potentially disrupting neighboring genes^5,6^.

In humans, naturally occurring double-strand breaks (DSBs) are typically repaired accurately; however, occasionally, inherently mutagenic MMEJ repair results in genetic errors. Microdeletion variants account for 20–25% of all clinically pathogenic sequence variants^7–9^. The majority of these mutations display a local sequence signature characteristic of deletions through µHs and are often three adjacent base pairs in length. Using this natural MMEJ mechanism for frame-retaining DSB repair of coding sequences offers biotechnological opportunities.

MMEJ as a repair mechanism for DSBs induced by CRISPR–Cas is conserved across a broad spectrum of organisms, ranging from Hydrozoa^10^ and plants^11^ to zebrafish^12,13^, *Xenopus*^14^ and humans^15,16^. Such MMEJ repair occurs in a nonrandom fashion and is predictable by algorithms and deep learning models, such as inDelphi^17–19^. This predictability has been harnessed to establish programmable smaller^17^ and larger^20,21^ deletions after DSB repair but never transgene insertions. While MMEJ-mediated approaches have been successfully used for integration (for example, GeneWeld^22^ and PITCh^23–25^), these did not offer control over gene-editing outcomes at genome–transgene repair boundaries. On the other hand, prime editing’s effectiveness depends on the coordination of multiple components and is traditionally restricted to edits ranging from 1 to ~50 bp, rendering larger insertions inaccessible^26^. New tools that combine prime editors with serine integrases, such as TwinPE^27^, PASTE^28^ and PASSIGE^29^, have been shown to enable larger DNA insertions yet leave a footprint, making them less suitable for protein tagging applications.

The CRISPR–Cas system has been widely adopted in biotechnology and basic research. Here, we explore the insertion of transgenic cassettes using the CRISPR–Cas system and the predictable nature of DSB repair mechanisms when introducing exogenous genetic material. We harnessed deep learning models, pretrained on DNA repair outcomes, to develop optimal rules for designing repair arms, both to integrate transgenic cassettes and to establish small point mutations. This results in predictable editing outcomes driving intended edits and integrations.

We used tandem repeats of µHs, placed at the edges of transgene cassettes to facilitate on-target integration by MMEJ using CRISPR–Cas. We find that DSB repair is nonrandom on the interface between the genome and such µH tandem repeat repair arms of transgenic cassettes in vitro and in vivo. Moreover, µH tandem repeat repair arms safeguard the boundaries during integration, precluding extensive DNA trimming. We deduced optimal design rules and showed integration using µH tandem repeats to be effective in cell contexts where HDR is largely ineffective, such as rapidly cycling vertebrate embryos (*Xenopus*) and adult postmitotic mouse neuronal cells. Lastly, we extend the notion of predictability to the rational design of small repair templates for the introduction of desired point mutations at permissive loci with single-stranded oligodeoxynucleotide (ssODN) donor templates.

## Cas9 integration with donor templates is nonrandom and predictable

**Fig. 1:**
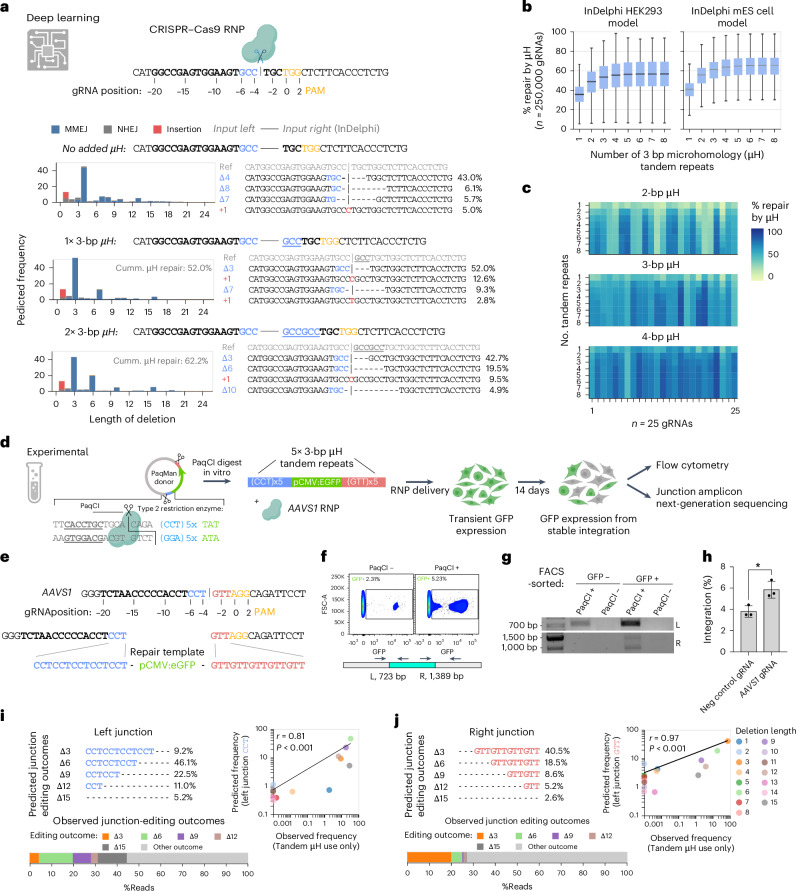
Modeling predicted gene-editing outcomes using inDelphi while providing synthetic µHs. **a**, Predicted editing outcomes are shown using inDelphi (HEK293T) on synthetic DNA. Adding tandem repeats of the bases left of the CRISPR–Cas cut site to the right of the cut affected the predicted editing outcomes. Cumulative µH repair is defined as the percentage of editing outcomes that mobilize (delete) synthetic µHs during repair. Iterative recutting of products is not computationally modeled. **b**, Modeling of expected editing outcomes across 250,000 distinct gRNAs target sites across human Chr1, when adding the 3 bp flanking the left site of the CRISPR–Cas cut site either as a single repeat (1×) or as tandem repeats (2×–8×). The percentage of repair by µH usage is shown. Box plots show the median, interquartile range (IQR) and whiskers extending to 1.5× the IQR with *n* = 250,000. **c**, Heat map highlighting the expected percentage of repair by µH as a function of the length of µH and the number of tandem repeats for 25 gRNAs, demonstrating that there is a sequence-context-specific optimal solution for maximizing the percentage of µH repair outcomes. **d**, Schematic of the experimental setup: PaqCI digestion releases the linear dsDNA donor, which contains 5× 3-bp µH tandem repeat arms, and is codelivered with RNP targeting *AAVS1*. **e**, Sequence of the target locus and 3-bp µH tandem repeat repair arms. **f**, After 14 days, flow cytometry indicates an increase in stable integration in cells transfected with the linear dsDNA template. **g**, Integration occurs specifically with PaqCI-linearized templates; circular templates show no detectable on-target integration. **h**, Quantification of integration efficiency of *AAVS1* gRNA compared to a negative control gRNA. Statistical analysis was performed using an unpaired two-tailed *t*-test; *P* = 0.021 (*n* = 3 independent biological replicates). Error bars represent the s.d. **i**,**j**, The InDelphi HEK293T model accurately predicts the observed frequency of distinct editing outcomes in the µH tandem repeat arms at both junctions. Data points are the means of three independent biological replicates. A two-sided Pearson correlation was applied (**i**, *r* = 0.815, *P* = 0.00022; **j**, *r* = 0.969, *P* = 1.10 × 10^−8^). No multiple comparisons were performed. Some schematics were created with BioRender.com. Source data

Endogenous DNA repair outcomes following DSBs induced by CRISPR–Cas (specifically *Streptococcus pyogenes* Cas9) are nonrandom and can be predicted on the basis of the local sequence context^15–18^. We explored whether one such algorithm, inDelphi^17^, could also predict editing outcomes at the interface between endogenous DSB edges and exogenous donor DNA. When the inDelphi model predicted a µH-mediated 4-bp deletion as the major editing outcome of an example sequence (Fig. 1a), adding the 3 bp present on the left side of the cut to the sequence right of the cut pivoted the most frequent predicted outcome toward a 3-bp deletion. This effectively removed the inserted 3-bp µH, overruling the previously dominant 4-bp deletion. Further repeating the 3-bp sequences in tandem increased the proportion of predicted editing outcomes that use an inserted artificial µH from 52% to 62% (Fig. 1a). Extending the in silico simulation to 250,000 putative guide RNA (gRNA) target loci on human chromosome 1 revealed an increase in artificial µH usage for DNA repair with an increasing number of tandem repeats, plateauing at five tandem repeats (Fig. 1b and Supplementary Fig. 1). The local sequence context strongly influenced the use of µH tandem repeats (Fig. 1c), suggesting that the optimal design needs to be computed for each gRNA and its surrounding genomic sequence.

Next, we experimentally investigated whether inDelphi predictions of repair outcomes between endogenous DSB edges and exogenous donor DNA would facilitate CRISPR–Cas-mediated knock-in. For this, the *AAVS1* landing site was targeted in HEK293T cells (Fig. 1d). We added five tandem repeats of 3-bp µH (5× 3-bp µH) to the left and right of the donor cassette, matching the sequence context left and right of the *AAVS1* cut site (Fig. 1e). We assessed the resulting scarring patterns and validated the predictability of DNA repair at genome–transgene borders and the increased frame retention. To more easily customize the donor edges without undesired 5′->3′ overhangs, we added two PaqCI type IIS endonuclease restriction sites invertedly flanking the donor cassette (pCMV:eGFP) for in vitro release of linear DNA (PaqMan plasmids; Supplementary Fig. 2a). PaqMan linearization facilitated on-target genomic integration (5.2% GFP^+^), whereas nonlinearized plasmid donor merely resulted in random integration (2.3% GFP^+^), demonstrated by boundary PCR analysis (Fig. 1f,g and Supplementary Fig. 2b). On-target integration only occurred with an *AAVS1-*targeting ribonucleoprotein (RNP) and never with control RNP (gRNA target site not present in the human genome) (Fig. 1h and Supplementary Fig. 2c).

Using 3-bp µH tandem repeat repair arms provided us with a unique way to sample the distribution of editing outcomes at the interface between endogenous DNA and exogenous cargo. Targeted amplicon sequencing of the boundary PCR products revealed that the rate of µH tandem repeat use after DNA integration observed experimentally correlated well with the inDelphi predictions at the left (*r* = 0.81, *P* < 0.001) and right (*r* = 0.97, *P* < 0.001) junctions (Fig. 1i,j and Supplementary Fig. 3a). Furthermore, 73% of the reads at the left junction boundary did not trim into the genome. Of these, 63% (46% of total reads) also did not trim into the transgene (Supplementary Fig. 3b,c). On the other hand, 78% of the reads on the right junction did not trim into the genome and 55% of these (43% of total) also did not trim into the transgene. The most common genetic lesion after trimming-free integration was the loss of one or more of the µH tandem repeats in the repair arms (45% of total reads on the left and 28% of total reads on the right) (Supplementary Fig. 3b,c). To investigate integration in a clinically relevant site for chimeric antigen receptor (CAR) T production, we introduced a second-generation CAR^30^ into the *TRAC* locus (Supplementary Fig. 4a,b). Using 6-bp frame-retaining µH repair arms, we found boundary products, also detected in NHEJ-driven HITI^31^ and HDR-mediated integration methods performed in parallel (Supplementary Fig. 4c,d).

Thus, merely 3–6 bp of µHs were sufficient to mobilize DNA donor arms during CRISPR–Cas knock-in. In conclusion, Cas9-mediated MMEJ integration is nonrandom and predictable.

## µH tandem repeat repair arms safeguard the genome and integration efficiency is influenced by local sequence context

**Fig. 2:**
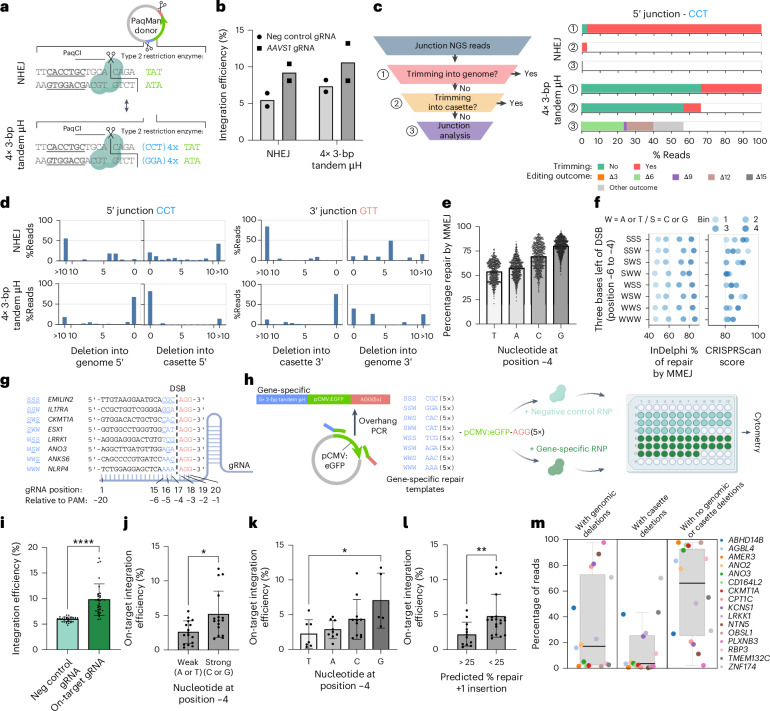
µH tandem repeat repair arms protect the genome and the relationship between integration efficiencies and local sequence context. **a**, Schematic representation of the experimental setup comparing NHEJ integration (no repair arms) to 4× 3-bp µH tandem repeat repair arms using PaqMan plasmids. **b**, Comparison of µH tandem repeat-mediated and NHEJ integration efficiencies (*n* = 2 independent biological repeats). **c**, Visualization of genome-editing outcomes on both genome–transgene junctions showing the percentage of reads that trimmed the genome (1), the percentage of reads that trimmed the cassette (2) and specific editing outcomes of reads that trimmed neither the genome nor the cassette (3). **d**, Quantification of genome-editing outcomes on both genome–transgene junctions demonstrating that NHEJ leads to extensive trimming, while 4× 3-bp µH tandem repeat arms protect both the genome and the transgene cassette. **e**, In the absence of exogenous DNA, in silico modeling predicts that the nucleotide at position −4 will influence the percentage of repair outcomes that is expected to be driven by MMEJ (total *n* = 10,813,171; plotted random subselection of 500,000 data points). **f**,**g**, The 32 gRNAs designed to target coding exons of nonessential genes with four in each of eight classes covering all possible permutations of strong (G or C) and weak (A or T) bases at 3 bp left of the DSB. Each class was composed of four gRNAs binned across the inDelphi-predicted percentage of repair by MMEJ and had similar expected on-target efficiencies (CRISPRScan scores). **h**, For each gRNA, a distinct dsDNA repair template was generated with 5× 3-bp µH tandem repeat repair arms matching the gRNA-specific context left of the DSB and 5× 3-bp µH tandem repeat repair arms matching the AGG right of the DSB. These were delivered with nontargeting control RNP (top) or gene-targeting RNP (bottom) to HEK293T cells. Each data point represents an independent biological replicate. **i**, Integration efficiencies at day 14, determined by flow cytometric quantification of GFP⁺ cells. Statistical analysis was performed using a Mann–Whitney test (two-tailed, exact, *P* = 6.23 × 10⁻⁷, *n* = 32). **j**, Quantification of on-target integration efficiencies comparing the presence of a strong or weak base at position −4, just left of the DSB. Statistical analysis was performed using a Mann–Whitney test (two-tailed, exact, *P* = 0.0211, n = 16. **k**, On-target integration efficiencies by base identity at position −4, with guanine showing the highest. Each point represents the mean of three biological replicates. Sample sizes: T, *n* = 7; A, *n* = 9; C, *n* = 11; G, *n* = 5. Statistical analysis was performed using a Kruskal–Wallis test (*P* = 0.0445) with Dunn’s post hoc test (two-sided, corrected for six comparisons); T versus G, adjusted *P* = 0.0397. **l**, inDelphi modeling of the junction product between the sequence left of the DSB and the dsDNA donor. A higher percentage of predicted editing outcomes that have a +1 insertion will result in a lower on-target integration efficiency. Samples were grouped on the basis of the predicted percentage repair with +1 insertion (>25% and <25%). Statistical analysis was performed using a Mann–Whitney test (two-tailed, exact, *P* = 0.0092, *U* = 54, *n*₁ = 12, *n*₂ = 20). In **i**–**l**, error flags represent the s.d.; the center is the mean and each data point represents the mean of three independent biological replicates. **m**, NGS of left (5′) junction product and the percentage of reads containing genomic deletions or cassette deletions or neither genomic nor cassette deletions (*n* = 16 genes, each analyzed by sequencing after equimolar pooling of DNA from three independent biological replicates). Box plots show the median, IQR (box) and whiskers extending to 1.5× the IQR. Some schematics were created with BioRender.com.

Next, we benchmarked our methodology to NHEJ-mediated gene cassette knock-in, such as HITI, which does not use homology arms^31^. PaqMan plasmid donors (Fig. 2a and Supplementary Fig. 2d) showed no detectable differences in integration efficiencies when using either zero (NHEJ, 9.3%) or four (10.7%) 3-bp µH tandem repeats matching the *AAVS1* target site in HEK293T cells (Fig. 2b) (*P* > 0.05). When NHEJ was used, however, amplicon sequencing revealed extensive deletions at the genomic integration site (95% of reads) (Fig. 2c,d and Supplementary Fig. 2d). All remaining reads showed substantial trimming of the transgene cassette. In contrast, using µH tandem repeat repair arms decreased DNA trimming both into the genome and on the repair cassette, with over 50% of reads free from any deletions in either direction.

Next, we tested whether the nucleotide composition of µH tandem repeat arms affected their integration efficiency. In silico simulation with the inDelphi HEK293 model for >10 million gRNAs across the human genome revealed variations in predicted repair outcomes driven by µH composition, particularly linked to the nucleotide at position −4 (counting the NGG protospacer-adjacent motif (PAM) as nucleotides 0–2) (Fig. 2e). G at position −4 was predicted to enhance integration over C, A or T and was independent of the PAM sequence used (Supplementary Figs. 5 and 6). No similar effects were noted for any other position in the gRNA (Supplementary Figs. 5 and 6). This indicated that the nucleotide located immediately to the left of the CRISPR–Cas-induced DSB (position −4) could be a parameter to improve integration.

To test this, we targeted 32 genes in HEK293T cells and codelivered target-specific repair templates with five µH tandem repeats. To avoid a potential negative selection effect, we chose nonessential genes^32^. We ensured that the gRNAs had similar predicted on-target efficiency and a balanced distribution across different G+C contexts (Fig. 2f). To directly assess whether the nucleotides at position −7 to −4 influence integration, we only considered gRNA target sites with AGG at nucleotides −3 to −1. The 32 targets were chosen to fall into one of eight classes, each representing a distinct combination of strong (G or C) or weak (A or T) bases at positions −4 to −7 (*n* = 4 per class) (Fig. 2f,g). Within each class, we binned gRNAs according to predicted MMEJ repair usage. Target-specific repair templates, incorporating five 3-bp µH tandem repeats, were generated by overhang PCR (Fig. 2h).

Across all 32 targets, we observed a median 1.6-fold increase in integration efficiencies comparing on-target RNP to negative control RNP (median on-target integration of 3.61%, *P* < 0.0001, *n* = 32) (Fig. 2i). Next we assessed whether genomic µHs flanking the DSB compete with synthetic µHs at the genome–cassette interface. There was no correlation between on-target integration efficiency and inDelphi-modeled MMEJ repair at the DSB in the absence of exogenous repair templates (Supplementary Fig. 7a). This suggests that such local motifs flanking the DSB do not influence integration efficiency when a repair cassette is provided, meaning that preselecting gRNA target sites to avoid them is not necessary for successful integration. gRNAs that had a strong base (G or C) at nucleotide −4 drove integration at a median 1.8-fold more efficiently than those with a weak base (*P* < 0.05) (Fig. 2j). We found a hierarchical trend at nucleotide −4, where G (7% ± 4%), C (4.3% ± 2.9%), A (2.8% ± 1.3%) and T (2.2% ± 2.1%) influenced the use of µH-mediated integration rates (Fig. 2k), completely matching the predicted distribution (Fig. 2e). Next, we used inDelphi to predict gene-editing outcomes at the left junction between the endogenous locus and the cargo template. We observed a moderate inverse correlation between integration efficiencies and the percentage of repair predicted to be a +1 insertion (*r* = −0.512, *P* < 0.01) and between integration efficiencies and the predicted percentage of perfect repair products (defined as having used one µH tandem repeat) (*r* = 0.51, *P* < 0.01) (Supplementary Fig. 7b–d). We observed a median 2.2-fold higher rate of integration efficiency at junction events where inDelphi predicted the editing outcomes to be <25% +1 insertions than >25% +1 insertions (*P* < 0.01) (Fig. 2l). Of note, 5′ junction analysis revealed that, across these sites (*n* = 16 sequenced), a median of 83% ± 36% reads showed no genomic deletions, with 66% ± 35% exhibiting deletions in neither the genome nor the cassette (Fig. 2m). Additionally, we tested whether single-stranded DNA (ssDNA) repair templates could be used instead of double-stranded DNA (dsDNA), again using five 3-bp µH tandem repeats (Supplementary Fig. 8a). While this approach greatly reduced random integration when using a negative control gRNA (0.79% ± 0.11%; *n* = 8), the integration efficiencies were lower than with dsDNA templates (Supplementary Fig. 8b). On-target Integration efficiencies (*P* < 0.001 versus negative control) were comparable between sense (1.32% ± 0.32%, *n* = 4) and antisense (1.25% ± 0.18%, *n* = 4) orientations (Supplementary Fig. 8c,d).

On the basis of these observations, we propose the following for selecting gRNAs for optimal µH tandem repeat-mediated integration: (1) G nucleotide at position −4; (2) low rate (<25%) of predicted editing outcomes with a +1 insertion; and (3) a high percentage of predicted editing outcomes that use µH tandem repeats. Collectively, our findings demonstrate that deep-learning-based predictions improve integration outcomes and inform the rational design of optimal integration strategies.

## µH tandem repeat integration in vivo at the *hipp11* (*h11*) landing site of *Xenopus tropicalis*

**Fig. 3:**
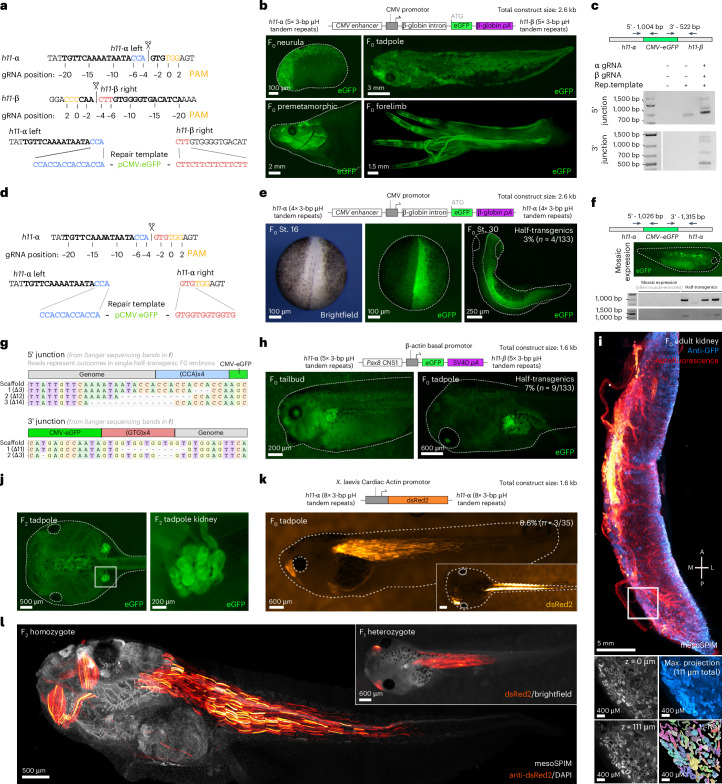
µH tandem repeat-mediated integration at stable landing site *h11* in *X.* *tropicalis* with germline transmission. **a**, Schematic of the CRISPR–Cas integration strategy. **b**, Mosaic but stable GFP expression after 5× 3-bp µH tandem repeat-mediated integration of a pCMV:eGFP in F_0_ founders at various developmental stages. **c**, Detection of PCR products demonstrating on-target integration into the *h11* locus. **d**, Schematic of the CRISPR–Cas integration strategy, using only the *h11-α* RNP. **e**, Unilateral nonmosaic GFP expression in F_0_ founders because of pCMV:eGFP integration into the *h11* locus at the two-cell stage (half-transgenic embryos). **f**, Nonintegrative mosaic expression pattern in muscle cells. Junction PCR analysis shows that this represents merely transient expression as correct junction products can only be detected in half-transgenic animals shown in **e**. **g**, Sequencing of junction products reveals usage of µH tandem repeats in 60% of reads (*n* = 5). **h**, Tissue-restricted expression pattern of *pax8*-CNS1:eGFP knocked in at the *h11-α* and *h11-β* loci in the F_0_ generation by five µH tandem repeats is observed in 7% of the injected embryos (*n* = 133). **i**, Benchtop mesoSPIM whole-organ imaging of a kidney from an adult F_0_ *pax8*-CNS1:eGFP founder, confirming stable integration and expression in renal tubules amenable for U-Net-based segmentation. **j**, Reporter expression in the embryonic kidneys of the F_2_ generation. **k**, Tissue-restricted expression pattern of CarAct:dsRed knocked in at the *h11-α* locus in the F_0_ generation by eight µH tandem repeats is observed in 8.6% of injected embryos (*n* = 35). **l**, Benchtop mesoSPIM imaging of F_1_ and F_2_ CarAct:dsRed knock-in animals revealing stable and strong tissue-restricted transgene expression.

Existing transgenesis methods (I-SceI^33^ and REMI^34^) to generate reporter lines in *Xenopus* are limited to random and multiple integration events. We identified a conserved *h11* locus^35^ on chromosome 1 of the *X.* *tropicalis* genome, in the intergenic sequence between *drg1* and *eif4enif1*, as a potential landing site for stable transgene integration. *X.* *tropicalis h11* flanking gene models showed direct synteny with chicken, pig, human and rat orthologs (Supplementary Fig. 9a)^36^. We identified two gRNAs (*h11*-α and *h11*-β), spaced 767 bp apart, and verified efficient editing activity (*h11*-α, 91% ± 9%; *h11-*β, 80.3% ± 2%) (Supplementary Fig. 9b–f). Linear donor DNA containing four 3-bp µH tandem repeat repair arms corresponding to *h11*-α on the left and with *h11*-β on the right was generated by overhang PCR of a plasmid encoding CMV:eGFP (Fig. 3a). We coinjected *h11*-α and *h11*-β Cas9 RNP together with the 3-bp µH tandem repeat donor template into both blastomeres of two-cell stage embryos and consistently observed eGFP expression across developmental stages, indicative of stable integration events (Fig. 3b and Supplementary Fig. 10d). PCR analysis of embryo pools (*n* = 25) revealed deletion of DNA between the *h11*-α and *h11*-β target sites (Supplementary Fig. 10a,b) and PCR junction fragments indicative of exogenous cassette integration in *h11* (Fig. 3c and Supplementary Fig. 10c,e). To further simplify the procedure, freshly fertilized embryos were targeted with only *h11-*α gRNA and a CMV:eGFP transgene containing four 3-bp µH tandem repeats (Fig. 3d). Furthermore, 3% of embryos (4 of 134) were half-transgenic, suggesting that integration occurred at the two-cell stage (Fig. 3e), clearly distinguishable from embryos with a mosaic expression pattern (Fig. 3f). Junction PCR products indicative of on-target integration were present for half-transgenic embryos but never for embryos with mosaic eGFP expression (Fig. 3f). Sequencing confirmed the usage of µH tandem repeats for MMEJ-mediated repair (60%; *n* = 5) (Fig. 3g).

One application of µH tandem repeat-mediated integration is characterizing *cis*-regulatory elements by integrating a candidate noncoding element with a minimal promoter and analysis of reporter expression levels and tissue specificity^37^. Such assays, ideally, require the number and sites of integration to be controlled^38,39^. Therefore, a *pax8*-CNS1:eGFP construct was targeted to the *h11* locus^40^. In 7% of the injected embryos (9 of 133), eGFP expression was observed in the pronephros, otic vesicle and, to a lesser extent, the neural crest, consistent with the described activity of the *cis*-regulatory element (Fig. 3h and Supplementary Fig. 11a)^40^. Integration resulted in stable and persistent transgene reporter activity observed in the kidney tubules of adult F_0_ frogs (Fig. 3i and Supplementary Video 1). Germline transmission was confirmed in 50% (*n* = 6) of F_0_ founder animals crossed with wild type and 33% ± 12% of F_1_ embryos exhibited tissue-specific GFP expression, which was then outcrossed to obtain stable F_2_ animals (Fig. 3j, Supplementary Fig. 11b,c and Supplementary Video 2).

The µH tandem repeat-mediated integration approach was further validated in vivo by integrating a *Xenopus* cardiac actin (CarAct):dsRed2 reporter cassette (Supplementary Fig. 11d)^38,41^. Strong nonmosaic muscle-specific dsRed2 expression was observed in 8.6% (3 of 35) of the F_0_ animals (Fig. 3k). Germline transmission was successfully confirmed in both assessed founder animals showing transmission rates of 10.5% and 45.5%, respectively (Fig. 3l, Supplementary Fig. 11e and Supplementary Video 3). In F_2_ homozygotes, we confirmed tissue-specific dsRed2 activity in myotomes (Fig. 3l and Supplementary Video 4) and found single-copy integration of the reporter construct at *h11* (Supplementary Fig. 11f). Taken together, we successfully achieved single-copy integration at the *h11* landing site in *X.* *tropicalis* of multiple donor templates without position effects or generational silencing.

## µH tandem repeats enable endogenous protein labeling in *X.tropicalis*

We next explored whether our transgene integration approach was suitable for endogenous protein tagging in *X.* *tropicalis*. Predicting integration scores for each possible gRNA target in the final 3′ exons revealed that 3-bp µH tandem repeats enable efficient tagging of 3% of genes and satisfactory targeting of 16%. Incorporating 6-bp µH tandem repeats instead improved design flexibility, ensured frame preservation and was predicted to increase the percentage of efficiently (35%) and satisfactorily (51%) targetable genes (Supplementary Fig. 12).

**Fig. 4:**
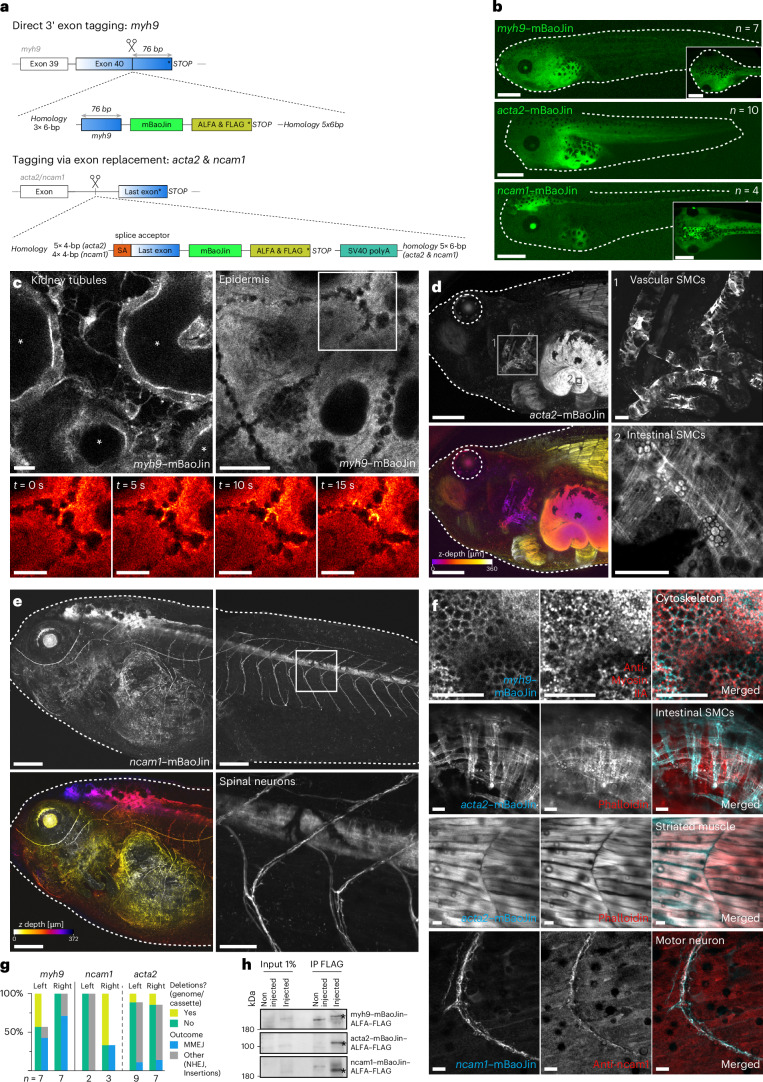
Endogenous fluorescent protein tagging in *X.* *tropicalis.* **a**, Schematic representation of the repair templates for endogenous gene tagging. Coding sequences linked with GSG linkers. **b**, Unilateral (Myh9 and Acta2) and bilateral (Ncam1) mBaoJin expression in F_0_ animals because of endogenous gene tagging. Scale bars, 500 μm. **c**, Imaging of tagged Myh9 in a living stage 45 tadpole. Top left, kidney tubules with a luminal Myh9 layer (*tubular lumen) and Myh9 signal in intertubular fibroblasts. Top right, epidermal cells showcasing the role of Myh9 in cell–cell adhesions. Bottom right, live imaging of actomyosin dynamics within cell–cell boundaries. Scale bars, 10 μm (top) and 5 μm (bottom). **d**, Imaging of tagged Acta2 in a living stage 45 tadpole. Left, overview showing fluorescence signal in intestinal smooth muscle cells (SMCs), vascular SMCs, heart muscle and skeletal muscle. Line-scanning artifacts in heart muscle because of heartbeat during acquisition. Gamma correction of 0.2 because of strong signal from intestinal SMCs. Top right, vascular SMCs wrapping around developing blood vessels. Bottom right, actomyosin network of the two perpendicular layers of intestinal SMCs. Scale bars, 250 μm (left) and 25 μm (right). **e**, Imaging of tagged Ncam1 in a living stage 45 tadpole. Expression of Ncam1 in the central and peripheral nervous system. Bottom right, spinal cord with branching motor and sensory neurons. Scale bars, 200 μm (top and bottom left) and 50 μm (bottom right). **f**, mBaoJin signal (cyan), immunofluorescence staining (red) and overlay in stage 45 fixed tadpoles. Top, intracellular Myh9 network in the epidermis. Middle top, intestinal SMCs in a unilaterally transgenic tadpole. Bilateral origin of SMCs leading to mosaic expression of labeled Acta2. Middle bottom, Striated skeletal muscle. Bottom, tail motor neuron. Scale bars, 10 μm. **g**, Repair outcomes of genome–cassette boundaries. **h**, Western blots detecting tagged endogenous protein.

Next, we targeted the last exon of *myh9* with a transgenic cassette containing the remaining 3′ exon fragment after the DSB, the fluorescent protein mBaoJin (a monomeric StayGold), an ALFA tag and a 3×FLAG tag^42–44^, flanked by 6-bp frame-retentive tandem repeats on the left and the right. The half-transgenic mBaoJin signal was detected in 0.90% (*n* = 222) of the injected embryos (Fig. 4a,b and Supplementary Tables 4 and 5). Live fluorescence imaging revealed intricate Myh9 protein dynamics at cellular junctions (Fig. 4c and Supplementary Video 5) and tagged Myh9 colocalized with anti-myosin signal in immunostainings (Fig. 4f).

While effective, the success of precise in-frame tagging within a 3′ exon is constrained by PAM availability and local sequence context, which influences integration prediction scores. Even with 6-bp tandem repeats, 14% of *X.* *tropicalis* genes were predicted to be untargetable at high efficiencies (Supplementary Fig. 12). Targeting the last intron, however (Supplementary Fig. 13), allows greater design flexibility on the repair arms, as frame retentiveness is no longer required and is predicted to be efficient for 98.4% of *X.* *tropicalis* genes. Using a repair cassette containing a splice acceptor, the last exon sequence fused in frame to mBaoJin and a tag cassette (Fig. 4a), we successfully tagged *acta2* (0.81%, *n* = 1,299) (Fig. 4b,d) and *ncam1* (0.82%, *n* = 365) (Fig. 4b,e). Live imaging revealed expected expression patterns of mBaoJin-tagged Acta2 and Ncam2 (Fig. 4d–f and Supplementary Video 6). Boundary and whole-insert PCR products for *myh9* and *ncam1* confirmed single-copy integration into the genome (Supplementary Fig. 14a) and Sanger sequencing revealed MMEJ-mediated integration (Fig. 4g), next to other repair outcomes. We detected more homology repeats than expected (2–5 extra repeats) in some of the boundaries (Supplementary Fig. 14b), likely because of cassette amplification or sequencing artifacts. Lastly, immunoprecipitation using the FLAG tag in mBaoJin-positive embryos confirmed successful tagging for each protein (Fig. 4h).

## µH tandem repeat-mediated in vivo fluorescent tagging of Tubb2a in mice

Traditional HDR is ineffective in nonproliferating cells but NHEJ-dependent HITI is frequently used^31,45,46^. Thus, we asked whether addition of frame-retentive µH tandem repeat repair arms could concurrently activate NHEJ and MMEJ, potentially increasing the proportion of in-frame tagged repair products.

**Fig. 5:**
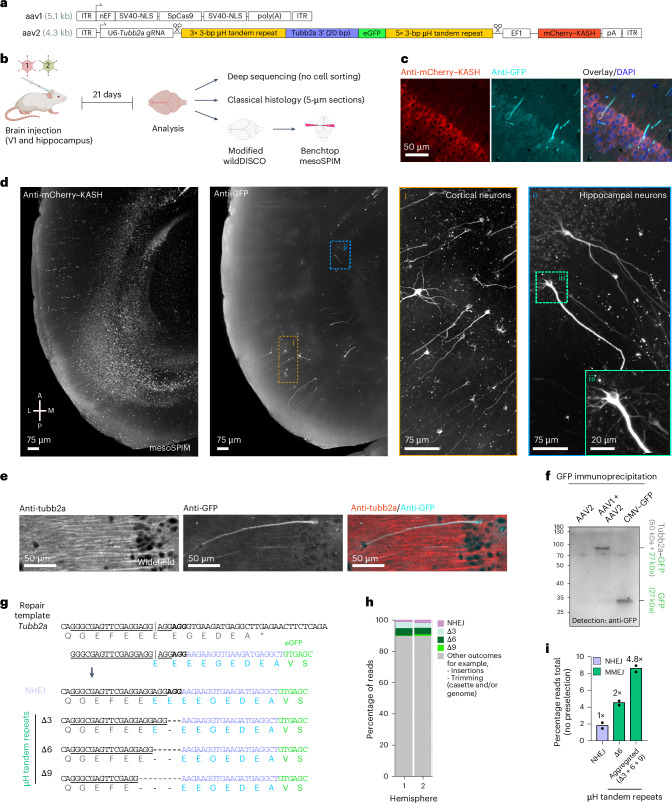
Endogenous fluorescence tagging of Tubb2a in vivo in adult mouse brains by µH tandem repeat-mediated integration. **a**, Schematic of AAV constructs for targeted eGFP knock-in at the 3′ CDS of *Tubb2a*. **b**, Schematic of the experimental setup and subsequent analysis. **c**, Histology of brain tissue and immunofluorescence detects eGFP-tagged Tubb2a in individual neurons. **d**, Benchtop mesoSPIM light-sheet imaging of wildDisco-cleared whole mouse brain shows eGFP-tagged Tubb2a in cortical and hippocampal neurons. **e**, Representative widefield immunofluorescence images showing GFP and Tubb2a expression in neurons. **f**, Western blot analysis comparing GFP immunoprecipitation from brains infected with either AAV2 alone, codelivered AAV1 and AAV2 or a control virus constitutively expressing GFP under the control of a CMV promoter. **g**, Sequence of the targeted *Tubb2a* locus (gRNA underlined, PAM in bold), the repair template and possible NHEJ and µH tandem repeat-mediated editing outcomes. **h**, Summary of integration outcomes using NGS reads spanning *Tubb2a*–eGFP amplified from two mouse hemispheres. **i**, Frequency of in-frame reads of *Tubb2a*–eGFP detecting either NHEJ or µH tandem repeat-mediated integration outcomes as defined in **g**. Each data point represents a single, independently injected brain hemisphere zone from the same mouse. Some schematics were created with BioRender.com.

We targeted the 3′ end of *Tubb2a*, a neuronal-specific tubulin localizing to both axons and soma^47^, for in-frame eGFP tagging. We performed in vivo transduction by adeno-associated virus (AAV) into adult mouse brains (Fig. 5a,b). One AAV carried Cas9, while the other carried *Tubb2a*-targeting gRNA, promoterless eGFP and a ubiquitous promoter driving mCherry for assessing cargo delivery. Then, 3 weeks after transduction, eGFP-positive neuronal cells, featuring eGFP-tagged Tubb2a protein driven from the endogenous *Tubb2a* promoter, were observed by classical histology (Fig. 5c) and in volumetric mesoSPIM imaging of a whole-mount mouse brain optically cleared by modified wildDISCO^48^ (Fig. 5d and Supplementary Video 7). Cortical and hippocampal neurons with eGFP expression along their projections were seen exclusively in virus-infected areas. eGFP expression also colocalized with Tubb2a (Fig. 5e). Compared to a control mouse with AAV-driven widespread eGFP expression (not fused to any protein), immunoprecipitation and western blot analysis detected a band at the combined sizes of Tubb2a and eGFP, demonstrating that eGFP was exclusively linked to *Tubb2a* (Fig. 5f). Next, we deep-sequenced the expected *Tubb2a*–eGFP junction site in two independently injected mouse hemispheres. Compared to earlier studies^31^, we did not preselect for cells expressing eGFP, thus getting an unbiased view of the gene-editing outcomes at the expected junction site. While we detected NHEJ-mediated gene tagging, it accounted for 1.8% of editing outcomes (Fig. 5g,h). MMEJ-mediated mechanisms were active in postmitotic cells as we observed 8.6% ± 0.5% of gene-editing outcomes that used µH tandem repeat-dependent repair. As predicted by inDelphi, the most common editing outcome was a deletion of 6 nt occurring at a frequency of 4.5% ± 0.4%. As such, our design strategy and use of µH tandem repeat repair arms increased the number of reads containing in-frame mutations 4.8-fold and rate of scar-free gene tagging 2-fold when compared to reads containing HITI or NHEJ outcomes (Fig. 5i).

Taken together, µH tandem repeat-mediated integration dramatically increased the efficiencies of in-frame gene tagging in mouse brains by engaging not only the NHEJ but also the MMEJ repair pathways.

## Pythia editing: precise genome rewriting by rational design of ssODN repair templates driving predictable DNA repair

**Fig. 6:**
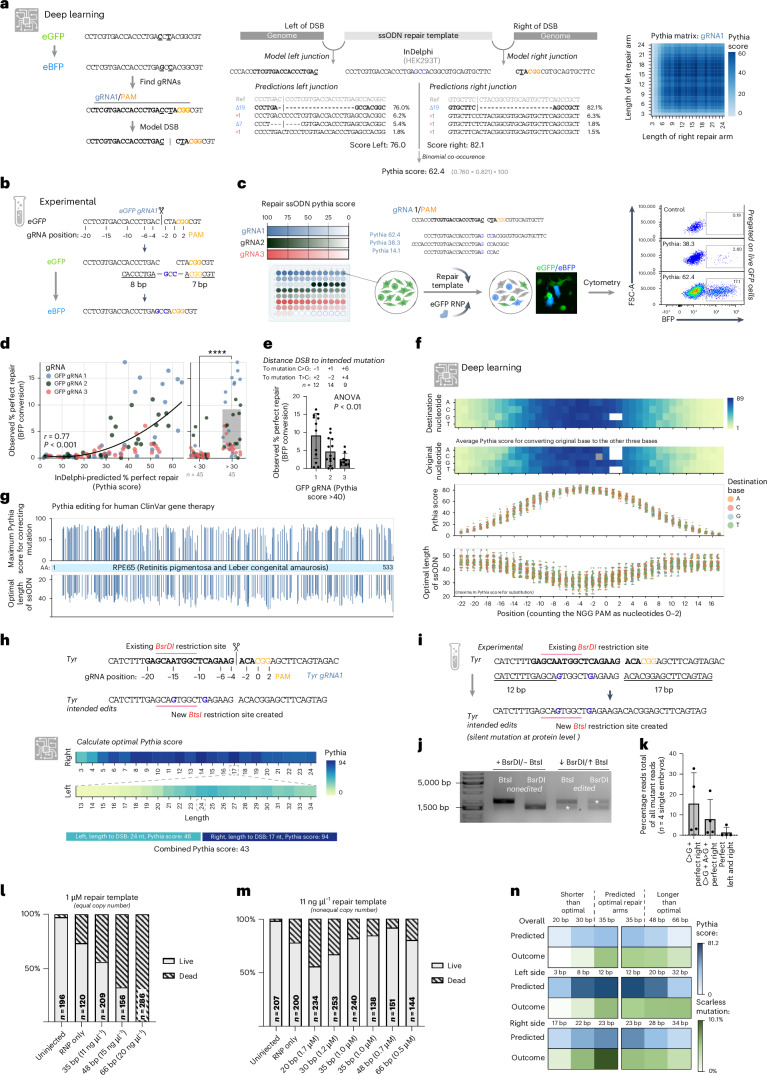
Pythia editing, leveraging predictability to create small point mutations in vitro and in vivo in *X.* *tropicalis.* **a**, eGFP-to-eBFP conversion can be achieved by establishing two point mutations. Schematic representation of Pythia, a bioinformatics pipeline, deploying the inDelphi model to calculate expected editing outcomes on both junctions, which yields a combined Pythia score defined as the binomial co-occurrence of the intended edit. Right, the Pythia scores for different repair arm lengths is depicted as a Pythia matrix. **b**, Strategy for converting eGFP into eBFP using an 18-bp-long ssODN designed by Pythia (homologous sequences underlined). **c**, Experimental setup for determining eGFP-to-eBFP conversion efficiencies using three different gRNAs, with 30 distinct ssODN repair templates binned across deciles of Pythia scores. **d**, Scatter plot showing a direct correlation between Pythia scores and fluorescence conversion, across all three tested gRNAs (Spearman’s two-tailed, exact, *P* = 3.66 × 10^−15^, *ρ* = 0.774, *n* = 90). Comparison of conversion rates between ssODN repair templates with a predicted Pythia score of below and above 30. Samples were grouped on the basis of the predicted percentage repair: <30%, *n* = 45; >30%, *n* = 45. Statistical analysis was conducted using a Mann–Whitney test (two-tailed, exact, *P* = 3.20 × 10^−12^, *U* = 148.5, *n*₁ = 45, *n*₂ = 45). Box plots show the median, IQR (box) and whiskers extending to 1.5× the IQR. **e**, The distance between the induced DSB and the site of the intended point mutation influences the median percentage of gene conversion. Statistical analysis was conducted using a one-way two-sided analysis of variance (*P* < 0.01). Sample sizes: gRNA1, *n* = 12; gRNA2, *n* = 14; gRNA3, *n* = 9. Error bars represent the s.d. In **d**,**e**, each data point represents the mean of three independent biological replicates. **f**, Modeling of potential Pythia editing outcomes for 35 gRNAs targeting the *X.* *tropicalis tyr* gene. From top to bottom, the average Pythia score for converting a base to one of the other three bases is shown, plotted first for each destination nucleotide at each position and below for each original nucleotide at each position. Scatter plot of maximum Pythia scores for optimal ssODN design at each position and the length of optimal ssODN (*n* = 75; each data point represents one in silico simulation). Box plots show the median, IQR (box) and whiskers extending to 1.5× the IQR. **g**, At-scale modeling of Pythia editing for restoring human *RPE65* pathogenic missense variants annotated in ClinVar to restore the wild-type amino acid. For each variant and the closest gRNA, the maximal achievable Pythia score (top) and the length of the optimal repair ssODN repair template (bottom) are shown. **h**, Strategy for establishing two silent point mutations in the *X.* *tropicalis tyr* gene, using an RNP and a 41-bp ssODN repair template as designed using the Pythia pipeline. **i**, Schematic of experimental design to detect and quantify successful editing events. **j**, Evidence of gene editing by restriction digest. **k**, Quantification of NGS amplicon read analysis. Each data point represents one unique embryo that was individually sequenced (*n* = 4). Error bars represent the s.d. **l**, Embryonic survival rates after injection with RNP and 1 µM ssODN template. Increased template length significantly correlates with increased lethality (Pearson’s *r* = 0.9440, one-sided *P* = 0.0280). **m**, Survival rates at a fixed nucleotide concentration. No significant correlation between molarity and lethality (Pearson’s *r* = 0.6047, one-sided *P* = 0.0752). **n**, Predicted repair outcomes (blue) versus sequencing results (green). Left, increasing Pythia score leads to higher perfect repair outcomes in all but one site. Some schematics were created with BioRender.com.

Because junctional products of µH tandem repeat-mediated integration were successfully predicted, we asked whether the predictive power of inDelphi could also be used to design more customized editing strategies. For example, would the model be able to predict the optimal repair sequence to maximize small but precise edits? To investigate this, we used ssODN templates to obtain gene edits exploiting single-strand templated repair (SSTR) through the Fanconi anemia (FA) DNA repair pathway^49^. Previously, numerous studies focused on enhancing gene-editing efficiency using HDR by adjusting the lengths of repair arms^50^, chemically modifying repair templates^51^ or inhibiting DNA repair regulators^52^. We next investigated whether inDelphi could be used for optimal ssODN repair design, forecasting predicted gene-editing efficiencies and the ratio of intended versus unintended editing outcomes. We used an eGFP-to-eBFP conversion assay^53^, which depends on the change of two nucleotides (CCT to GCC) to explore the design space by inDelphi predictions. We computed the predicted percentage of on-target repair as a function of both the left and right repair arm lengths and calculated the chance for overall perfect repair as the joint probability of perfect repair occurring between the genome and both repair arms (Fig. 6a). Because this extended the use of the inDelphi model beyond previous applications, we termed this approach Pythia, in reference to the priestess at the Greek temple of Delphi in antiquity^54^. We introduce a bioinformatics-based solution for generating ‘Pythia matrices’ (Fig. 6a and Supplementary Fig. 15), which depict the predicted gene-editing efficiencies in relation to the lengths of both the left and right repair arms. Next, we investigated whether Pythia predictions correlated to experimental observations by designing repair templates with high and low Pythia scores (Fig. 6b).

For each eGFP gRNA (*n* = *3)*, we generated 30 repair templates with three repair templates in each decile of Pythia scores (bin) and quantified eGFP-to-eBFP conversion rates in HEK293T cells (Fig. 6c and Supplementary Table 6). This revealed gene-editing efficiencies of up to 18%, adhering to a monotonic correlation between Pythia prediction matrices and experimentally determined conversion rates (combined Spearman correlation *r* = *0.77*, *P* < 0.001) (Fig. 6c,d and Supplementary Fig. 16). As the distance from the intended base-pair modification to the DSB increased, gene conversion efficiency decreased, a trend accurately predicted by the Pythia matrices (Fig. 6e).

Next, we explored computationally whether the predictability would allow us to model the editing window for small but precise nucleotide substitutions. We computed the maximum Pythia score to establish individual base changes at positions −22 to +17 to all three possible nucleotide substitutions for 35 distinct gRNAs. This revealed no preference in substitution efficiency, suggesting that all possible substitutions are theoretically achievable (Fig. 6f). Furthermore, the greater the distance between the intended substitution and the DSB, the lower the highest possible Pythia score was. Thus, a longer ssODN template is needed to achieve an optimal Pythia score (Fig. 6f). Notably, at the individual gRNA level, sequence contexts exerted a profound influence, which led to considerable variation in the optimal ssODN repair length. Our model suggested that a window of −11 (mean Pythia score 61.1 ± 6.3) to +4 (mean Pythia score 61.5 ± 4.6) from the cut site constituted a suitable window for Pythia editing. We predicted the optimal ssODN repair templates for gene correction of all *RPE65* missense mutations associated with retinal degeneration and annotated in ClinVar^55^ where a suitable gRNA was found in proximity (*n* = 248). We observed an average Pythia score of 84.4% ± 12.5%, with an ssODN length of 33.9 ± 5.7 nt (Fig. 6g). Given that 81% (*n* = 293) of *RPE65* missense mutations could be edited with a Pythia score > 60, Pythia editing may hold promise for clinical applications.

## Pythia editing in vivo: computationally guided design of repair templates for precise CRISPR–Cas editing

Lastly, we questioned whether Pythia editing could be validated by in vivo experiments in *Xenopus* embryos. For this, we chose to design an ssODN repair template to introduce two silent mutations (spaced 5 bp apart) in tyrosinase (*tyr*), a gene essential for pigmentation. Pythia predicted highly efficient repair between the right template arm and the genome (17-bp distance to cut site, Pythia score = 94) but suboptimal welding between the left repair arm and the genome (24-bp distance to cut site, Pythia score = 46), yielding a total Pythia score of 43 (Fig. 6h). In 65% (*n* = 20) of pigmented animals (class 3) (Fig. 6i and Supplementary Fig. 17a–c) that received a high dose of the RNP–ssODN mixture, we observed restriction enzyme patterns indicating successful insertion of the desired point mutations (Fig. 6j). Next-generation sequencing (NGS) analysis of four animals with altered restriction enzyme digest patterns revealed efficient repair between the ssODN and the genome to the right of the DSB, incorporating the C>G edit in 16% ± 7.5% of reads, with 51% of these also containing the expected A>G edit (8% ± 4.8% of total reads) (Fig. 6k). In contrast, repair on the left arm was less efficient (24-bp distance to cut site, Pythia score = 46), resulting in only 1.4% ± 1.42% of reads exhibiting perfect repair on both arms with the intended silent mutations.

To further increase editing efficiencies and assess whether Pythia scores were indicative of in vivo gene-editing efficiency in *Xenopus* embryos, we again targeted the *tyr* locus. We used a predicted optimal repair template generating a single-base substitution, two templates of decreasing repair arm length and Pythia score and two templates of increasing length but decreasing Pythia score (Supplementary Table 7). Injections of equimolar ssODN templates resulted in dramatic lethality (up to 100%) with increasing template length (Fig. 6l), indicating toxicity as a function of total nucleotide concentration. At equal, sublethal nucleotide concentrations, the predicted optimal template resulted in the highest percentage of scarless base substitution, compared to both longer and shorter repair templates (Fig. 6m,n). Even without preselection, we achieved up to 3.2% perfect base substitution in a pool of 75 injected embryos, gene-editing levels sufficient to enable germline transmission and the establishment of patient-mimicking genetic models in *X.* *tropicalis*.

We provide a freely accessible web tool (https://www.pythia-editing.org) to allow custom design strategies for base edits or integration using Pythia (Supplementary Figs. 12a and 17d).

## Discussion

Improvements in gene-editing strategies often rely on rational design or systematic protein engineering^56^. Alternatively, we used a pretrained model (inDelphi^17^) toward optimizing transgenic cassette integration, gene tagging and gene editing (Pythia). Exploiting a system of µH tandem repeats, our key finding is that DNA repair is predictable at the interface between Cas9-mediated breaks and exogenously delivered DNA, both in vitro and in vivo. We distill a rule set for selecting gRNAs driving high integration efficiencies and for designing said target-specific repair templates. As such, rational design of donor repair arms to maximize desired editing outcomes is achievable, substantially aided by the deep learning network, and delivers mechanistic insights into how genomic context (G at nucleotide position −4) impacts the efficiencies of gene integration. This is inverse to the relationship between the nucleotide at position −4 and the propensity to repair by a +1 insertion^17,19^. Of note, the presence of a G at this position predicts blunt DSB induction by CRISPR–Cas^57^, possibly directly connecting the Cas9 incision type to the preferential engagement of distinct DNA repair pathways. Together, this allows for gRNA selection and rational design of repair arms using deep learning approaches in a sequence-context-specific manner.

These findings have numerous applications in biotechnology. Some of the advantages of µH tandem repeat-mediated integration are that it is directional, single copy and locus specific, thus avoiding most of the drawbacks of other in vivo transgenesis techniques, such as positional effects in enhancer screenings^38,39^. We demonstrated that µH tandem repeat-mediated integration enables cargo insertion and endogenous tagging in human, mouse and *Xenopus*. A key advantage of such repair arms is their short lengths (6–15 bp), which simplifies the generation of repair templates that can be efficiently produced using straightforward overhang PCR methods, facilitating large-scale cell screening projects and reducing cargo size associated with viral delivery strategies.

By co-opting the MMEJ repair pathway, µH tandem repeat-mediated integration is applicable in certain cellular contexts when HDR is known to be inefficient or even inactive, such as in early developing vertebrates^50^ or postmitotic adult tissues such as the retina or brain^46^, providing potential for gene therapy approaches^58^. µH tandem repeat-mediated integration allows endogenous protein tagging, overcoming the limitations of previously reported HDR-mediated methods^59,60^. Although our approach achieved relatively low efficiencies (0.5–1%), it successfully targeted all three sites tested in *Xenopus* embryos. Because of the ease of delivery, a single-injection experiment can reliably produce more than ten half-transgenic founder animals, a sufficient number for establishing stable lines.

Together, we show that integration with tandem repeat repair arms is sufficient for predictable in-frame repair and offers higher predictability than error-prone NHEJ-based methods^31,61,62^. Notably, we demonstrate that µH tandem repeat repair arms safeguard the genome and the donor template from extensive deletions during DNA integration.

Next, we demonstrate the potential for transfer learning of pretrained deep learning models (such as inDelphi) toward optimizing gene editing. We establish a metric called Pythia score that provides a predictive measure toward the efficiency of establishing intended point mutations but not bystander mutations using CRISPR-mediated SSTR with ssODN repair templates. As such, rational design of mass-producible small ssODN repair templates specifically designed to maximize gene editing is possible.

We demonstrate single-base-pair substitutions in rapidly developing *Xenopus* vertebrate embryos with ssODN repair templates without resorting to host transfer methods^59^. Although our efficiency rates are modest, they align with previous studies conducted in rapidly developing zebrafish embryos^50,63^. These efficiencies can be potentially enhanced through modifications to the ssODNs^51^ or the addition of small interfering molecules targeting mediators of DNA repair pathways^64^. Next, these findings suggest that DNA repair outcomes can also be predictably influenced when using ssODN templates. This opens up new possibilities for enhancing Pythia-based integration by using ssODN or hybrid ssDNA templates to further reduce off-target integration and cellular toxicity^65^.

One limitation of our methodologies is their dependence on DSBs, which are known to activate the p53 pathway and can sometimes result in complex genomic rearrangements, including genomic deletions, chromosomal translocations and chromothripsis. While several alternative strategies, such as base editing^66^, prime editing^26^, integrase-based approaches^27–29^ and retrotransposons^67^, offer potential solutions, they are not without their own challenges. Meanwhile, base editing is constrained by its editing window, limited in the variety of genetic substitutions it can achieve^66^. Despite the inherent limitations associated with inducing DSBs, this study demonstrates that sequence context specificity can be leveraged to optimize outcomes of both gene integration, gene tagging and small base-pair exchanges. These findings highlight deterministic patterns underlying such editing events, opening avenues to further refine and optimize gene-editing tools relying on DSB repair mechanisms. Another limitation is that CRISPR–Cas carries a risk of off-target cleavage^68^; we mitigated this by consistently applying gold-standard off-target prediction algorithms^69^ to substantially reduce the likelihood of unintended edits.

Indeed, deep learning has been shown to effectively predict outcomes for CRISPR–Cas^17^, base editing^70^ and prime editing^71^. In our study, we revealed an unanticipated level of nonrandomness of DNA repair on the interface between the genome and exogenous donor DNA, which is explainable by deep learning models trained on CRISPR–Cas-induced DSB repair^17^. While our approach was validated experimentally, further transfer learning could be performed by fine-tuning models for emerging Cas nucleases exhibiting distinct incision patterns or by addressing cell-specific repair contexts. We believe that our findings open an unexplored design space to optimize genome rewriting and will serve as a primer for training additional cell-type-specific models^72^. This may have profound implications for CRISPR–Cas-mediated gene therapy approaches.

To facilitate easy access, we created an online tool for automated design of repair templates for both µH tandem repeat-mediated integration and Pythia editing (https://www.pythia-editing.org). Drawing inspiration from the ancient world, we named our approach Pythia after the high priestess at the Temple of Apollo in Delphi. Renowned for her perceived ability to foretell the future, the Pythia was a revered figure whose prophecies guided countless decisions in antiquity^54^. Like the Pythia, our methodology predicts outcomes, albeit in the realm of CRISPR–Cas genome editing.

## Methods

### Cell culture

HEK293T (American Type Culture Collection (ATCC), CRL-11268) were cultured as recommended by the ATCC. Cell lines tested negative for *Mycoplasma* and were authenticated by the suppliers.

### Modeling of gene-editing outcomes

The inDelphi model was obtained from GitHub (https://github.com/maxwshen/inDelphi-model) and deployed in a suitable Python virtual environment (https://github.com/XenoThomasNaert/Pythia-Editing). To investigate the impact of the number of tandem repeats on the expected percentage of perfect DNA repair, we developed custom Python code. The percentage repair by µH is defined as the sum of all repair outcomes that use at least one µH tandem repeat. The code iteratively analyzes µH tandem repeat lengths ranging from two to six and the number of tandem repeats from one to eight. This analysis was conducted using the inDelphi HEK293T or mouse embryonic stem cell (mESC) predictive model for the first 250,000 gRNA sites identified by presence of an NGG PAM, encountered in the human gencode v43 transcript sequences. For all HEK293T experiments, predictive modeling was performed using inDelphi’s HEK293T mode, whereas, for predictive modeling in *Xenopus* and mice, the mESC mode was used as it was validated as predictive in early-dividing *Xenopus* embryos^14^.

### Cloning and in vitro linearization of PaqMan repair plasmids and PCR generation of repair templates

Donor plasmid was assembled in a pUC19 backbone using Gibson cloning (NEBuilder HiFi DNA assembly master mix) and featured a pCMV-eGFP transgenic cassette flanked by zero, four or five µH tandem repeat repair arms and inverted PaqCI restriction enzyme sites. The insert was obtained from AAV-CMV-GFP, which was a gift from C. Cepko (Addgene, plasmid 67634; RRID:Addgene_67634). The pUC19 destination vector was commercially purchased (N3041S, New England Biolabs (NEB)). Inverted PaqCI sites and µH tandem repeat repair arms were added by overhang PCR before Gibson assembly. Linearization was performed by overnight digest at 37 °C of 10 µg of donor plasmid using 20 U of PaqCI (R0745, NEB) in 1× rCutSmart buffer (B6004S, NEB). Complete linearization was ensured using classical agarose gel electrophoresis.

Alternatively, repair templates containing µH tandem repeat repair arms were generated by overhang PCR using Phusion polymerase (Thermo Fisher, F530S) with primers designed to contain an overhang sequence containing the µH tandem repeat repair arms (listed in Supplementary Table 1). For in vitro use, PCR products were cleaned using a MinElute PCR purification kit (28004, Qiagen) and eluted in ultrapure water. For in vivo use, PCR products were cleaned by classical phenol–chloroform extraction with sodium acetate–ethanol precipitation and quantified using Nanodrop (ThermoFisher).

### µH tandem repeat-mediated integration in vitro

*AAVS1* gRNA was assembled using Alt-R CRISPR–Cas9 IDT CRISPR RNA (crRNA) and Alt-R CRISPR–Cas9 *trans*-activating crRNA (tracrRNA), according to the manufacturer’s instructions, by heating it to 95 °C and cooling it to room temperature, yielding a duplex at a final concentration of 1 μM. Cas9 protein (PNABio, CP01) was diluted to 166.67 ng μl^−1^ in 1× PBS. HEK293T cells were reverse-transfected using Lipofectamine CRISPRMAX (Thermo Fisher, CMAX00003) as follows. RNP was assembled by incubation for 5 min at room temperature of 1 μM gRNA duplex, 250 ng of Cas9 protein and 0.6 μl of Cas9 PLUS reagent (from CRISPRMAX kit) in 23 μl of Opti-MEM (Thermo Fisher, 31985070). Then, 200 ng of PaqCI (R0745, NEB) digest product was added to the RNP. Transfection complexes were generated by incubation at room temperature for 20 min of 25 μl of RNP repair template, 1.2 μl of CRISPRMAX transfection reagent and 23.8 μl of Opti-MEM medium. Resulting transfection complexes were mixed with 40,000 HEK293T cells (suspended in a total volume of 100 μl of DMEM) and plated on 96-well Nunclon plates (Thermo Fisher, 167008). Cells were cultured for 25 days and cell sorting for GFP^+^ cells was performed.

For *TRAC* CAR knock-in, gene editing was performed identically to above, with some exceptions. Specifically, a CD19-specific CAR expression construct based on pUC19-HDRT-TRAC-CD19.CAR-Cas12a.PAM.mutated (Addgene, plasmid 215769; RRID:Addgene_215769)^30^ was ordered synthetically. The construct consisted of P2A, CD19-Car, bHg poly(A) and 400 bp of classical HDR homology arms. For targeting the *TRAC* locus, we used the following gRNA 5′-AGCTGGTACACGGCAGGGTC-3′. Repair templates were generated containing classical HDR homology arms (400 bp), no repair arms (HITI) or tandem repair arms by PCR. We used 100 ng of repair template (instead of 200 ng) and transfection was performed 1 day after seeding of 20,000 cells in a 96-well plate.

For the 32-target experiment, gRNAs were designed for the coding sequence (CDS) from human genome assembly GRCh38 using a custom python script, identifying gRNAs with each permutation of strong (S) and weak (W) bases at positions −6 to −4 and AGG at positions −3 to −1 with NGG as the PAM at positions 0 to 2. Identified gRNAs were filtered for those with CRISPRScan scores^73^ exceeding 80. To avoid negative selection because of gene essentiality when targeting CDS, we filtered the gRNA list to exclude any gene occurring in DEG15 (ref. ^32^), a database of essential genes as determined from shRNA and CRISPR–Cas screens. Next, the eight classes of permutations involving S and W bases were sorted into bins. For each class, one gRNA was selected per bin, arranged according to the degree of sequence context µH, ranging from low to high. For each gRNA, the off-target profile was determined and deemed acceptable using Cas-OFFinder^69^ (list of gRNAs in Supplementary Table 2).

Gene editing was performed identically to above, with some exceptions. Specifically, we used 100 ng of repair template (instead of 200 ng), generated by overhang PCR as described above from AAV-CMV-GFP (Addgene, plasmid 67634; RRID:Addgene_67634) (Supplementary Table 3). Transfection was performed 1 day after seeding of 25,000 cells in a 96-well plate. Cells were sorted on days 2 and 15. Here, integration efficiency was defined as follows. All cells were pregated on live cells, using SYTOX deep red nucleic acid stain (1 µM final) (Thermo Fisher, S11380). Then, the percentage of GFP^+^ cells on day 15 was calculated as a proportion of the percentage of GFP^+^ cells at day 2, thus accounting for differences in initial transfection efficiency by transient expression of the pCMV:eGFP cassette on day 2. On-target efficiency was defined as the difference between the integration efficiency of on-target gRNA and that of negative control gRNA on day 15, thus identifying the level of true on-target integration.

For ssDNA donor experiments, dsDNA repair templates were generated by overhang PCR as described above from AAV-CMV-GFP (Addgene, plasmid 67634; RRID:Addgene_67634). ssDNA was generated and quality-controlled by the Guide-it Long ssDNA production system v1 (Takara Bio, 051818) according to the manufacturer’s instructions. Here, 50 ng of ssDNA repair template was used. On day 15, gene integration by GFP^+^ cells was quantified using flow cytometry. Living cells were pregated before gating for SYTOX deep red nucleic acid stain (1 µM final) (Thermo Fisher, S11380).

### µH tandem repeat-mediated integration and gene tagging in vivo in *Xenopus*

*X.* *tropicalis* animals were kept according to Swiss law for care and handling of research animals. Husbandry and treatment were approved by the local authorities (Veterinäramt Zurich). Gene symbols follow Xenbase (http://www.xenbase.org/, RRID:SCR_003280). For *Xenopus* experiments, repair templates for pCMV:eGFP, *pax8*-CNS1:eGFP and CarAct:dsRed2 were generated by overhang PCR as described above. For pCMV:eGFP (5× 3-bp µH tandem repeats) and *pax8*-CNS1:eGFP experiments, *h11*-α and *h11*-β gRNAs were assembled as follows: 1 µl of Alt-R CRISPR–Cas9 IDT crRNA (100 µM stock) and 1 µl of Alt-R CRISPR–Cas9 tracrRNA (100 µM stock) were mixed with 3 µl of nuclease-free duplex buffer (IDT) and heated at 95 °C for 5 min and allowed to cool to room temperature. For RNP assembly, 1.8 μl of Cas9 protein (1 μg μl^−1^, PNABio CP01) was mixed with 0.2 μl of gRNA and heated to 37 °C for 5 min, before adding repair template. The final injection mix consisted of 1 μl of *h11*-α RNP, 1 μl of *h11*-β RNP and 1 μl of repair template (stock concentration: 10 ng μl^−1^), thus yielding a final repair template concentration of 3.33 pg nl^−1^. Embryos were injected unilaterally at the two-cell stage. For pCMV:eGFP (4× 3-bp µH tandem repeats) and CarAct:dsRed2, we mixed Cas9 protein (3 μl at 1 μg μl^−1^, PNABio CP01) with gRNA (1 μl) and incubated for 5 min at 37 °C to assemble RNP. RNP was mixed with repair template at a ratio of 4:1; thus, adding 1 μl of repair template (10 ng μl^−1^) to the mix yielding a final repair template concentration of 2 pg nl^−1^. Embryos were injected at the one-cell stage immediately after cortical rotation, targeting the gray sperm entry point with 5–10 nl of injection mix.

Embryo development was monitored and, at Nieuwkoop–Faber stage 40, embryos were lysed (50 mM Tris pH 8.8, 1 mM EDTA, 0.5% Tween-20 and 2 mg ml^−1^ proteinase K) overnight at 55 °C. Three classes of embryos were lysed as follows: embryos with unilateral or bilateral nonmosaic fluorophore expression, embryos with mosaic expression often restricted to a subset of the muscle cells and control embryos of the same clutch that were not microinjected. After proteinase K inactivation, junction products between the *h11* locus and transgene cassette were picked up using PCR and subjected to Sanger sequencing. Whole-embryo bleaching, staining and clearing were performed as previously described^74^ using 1:250 anti-GFP (Aves, GFP-1020) and 1:250 anti-RFP (Rockland, 600-401-379-RTU).

For tagging, the repair templates including the homology arms (Supplementary Table 4) were ordered from Twist Biosciences, PCR-amplified, and phenol–chloroform-purified. RNP was assembled as described above and was coinjected with repair template (2–8 ng μl^−1^) and TRITC–dextran (0.5 ng μl^−1^; Sigma-Aldrich). Embryos were sorted for TRITC fluorescence the next morning and mBaoJin fluorescence was assessed at the tailbud stage. Stage 45 tadpoles were anesthetized in 0.02% MS-222 (Sigma-Aldrich, A5040) for confocal live-cell imaging. Then, tadpoles were tail-clipped for genomic DNA extraction. Tails were lysed (50 mM Tris pH 8.8, 1 mM EDTA, 0.5% Tween-20 and 2 mg ml^−1^ proteinase K) at 55 °C overnight and heat-inactivated at 98 °C. Boundary products were amplified using phusion polymerase (NEB, M0530L) and sent for commercial Sanger sequencing (Microsynth). Sequence alignments were performed in Benchling. The tadpoles were fixed in 4% PFA (Merck, 158127) at 4 °C overnight and permeabilized in PBS–Tween-20 (0.1%; PanReac AppliChem, A4974). Immunostaining for *myh9*-tagged, *acta2*-tagged and *ncam1*-tagged animals was performed by incubation at 4 °C overnight in 1:100 anti-myosin IIA (Sigma-Aldrich, M8064), 1:100 phalloidin–FluoProbes 647 (Interchim, FP-BA0320) or 1:10 anti-*Ncam1* (DSHB, supernatant, XAN-3 (clone 6F11)). The *myh9* and *ncam1* animals were further incubated at 4 °C overnight with 1:200 goat anti-rabbit IgG (H + L) DyLight 633 (Thermo Fisher, 35562) and 1:200 goat anti-mouse IgG (H + L) Alexa Fluor 633 (Thermo Fisher, A21050) respectively.

### Immunoprecipitation of endogenously labeled Myh9, Ncam1 and Acta2 in *Xenopus* embryos

*X.* *tropicalis* embryos displaying unilateral or bilateral mBaoJin expression in a tissue-restricted manner were snap-frozen in liquid nitrogen at NF stage 42–45. Protein extraction was performed by placing 2–5 embryos in a 1.5-ml tube containing 500 µl of immunoprecipitation lysis buffer (Pierce, 87787) solution with freshly added protease and phosphatase inhibitor cocktail (Thermo Fisher, 78440). Homogenization of the tissue was achieved by 15 strokes of a 21G needle, followed by 10 strokes of a 26G needle. After 15-min incubation on ice, lysates were centrifuged at 16,000*g* for 30 min at 4 °C. Protein lysates were transferred to a fresh 1.5-ml tube. Then, 1% of the input samples were used for western blotting and the remaining lysate was subjected to immunoprecipitation. For precipitation, the protein lysates were incubated with anti-DYDDDK magnetic agarose beads (Thermo Fisher, A36797) for 3 h at 4 °C. Resin-associated proteins were washed four times with lysis buffer and eluted with 4× Laemmli buffer (Bio-Rad, 1610747). Samples were subjected to western blotting as described below. Anti-FLAG (Sigma-Aldrich, F3165; 1:1,000) was used as the primary antibody and anti-mouse IgG–peroxidase (Sigma-Aldrich, A8924; 1:5,000) was used as the secondary antibody for protein detection.

### µH tandem repeat-mediated integration in vivo in mouse brain

pAAV-mTubb3 and pAAV-nEFCas9 were gifts from J. Belmonte (Addgene, plasmid 87116; RRID:Addgene_87116; Addgene, plasmid 87115; RRID:Addgene_87115). The sequence between AgeI and NdeI restriction sites was exchanged for a synthetic DNA fragment containing a gRNA targeting mTubb2a (5′-GGGCGAGTTCGAGGAGGAGG-3′), the 3′ Tubb2a sequence context, µH tandem repeat repair arms and eGFP to generate pAAV-mTubb2a. pAAV-nEFCas9 and pAAV-mTubb2a were packaged with serotype 8 and were generated by the Viral Vector Facility at the University of Zurich.

All procedures of mouse animal experimentation were carried out according to the guidelines of the Veterinary Office of Switzerland and following approval by the Cantonal Veterinary Office in Zurich (license 008/2022). Four C57BL/6 mice were used for virus injections. Mice were housed on a 12-h reversed light–dark cycle at an ambient temperature of between 21 °C and 23 °C, with the humidity level between 55% and 60%. Mice were anesthetized with 1.5–2% isoflurane mixed with oxygen and were head-fixed in a stereotactic frame (Kopf Instruments). Body temperature was maintained at ~37 °C using a heating pad with a rectal thermal probe. Vitamin A cream (Bausch & Lomb) was applied over the eyes to avoid dry eyes. After analgesia treatment (extended buprenorphine release EthiqaXR, 3.25 mg kg^−1^, subcutaneous; lidocaine over scalp), an incision was made on the scalp and small holes were drilled over bilateral visual cortex using the following coordinates: 3.5 mm caudal, 2.5 mm lateral relative to bregma and 0.5 mm ventral from the pia. We used 1:1 mixture of AAV-Cas9 (1.5 × 10^13^ genome copies (GC) per ml) and AAV-mTubb3 (2.3 × 10^13^ GC per ml) and injected 600 nl of AAVs in each hemisphere. To prevent virus backflow, the pipette was left in the brain for 5–10 min after completion of injection. Mice were housed for 3 weeks to allow for gene knock-in. Next, animals were killed and perfused using 4% PFA; brains were dissected and postfixed for 2 h in 4% PFA. Whole-brain staining was performed, adapted from previously described WildDisco^48,75^. Whole-mount brains were dehydrated to 100% methanol (high purity throughout this adapted wildDISCO, Supelco Emplura; Merck, 8.22283), delipidated with dichloromethane and bleached in 3% hydrogen peroxide prepared by diluting 30% H₂O₂ 1:10 in 100% methanol. Then, brains were permeabilized and blocked for 3 days using 10% donkey serum and 2% Triton X-100 in 1× PBS. Antibody staining was performed with 1:250 anti-GFP (Aves, GFP-1020) and 1:250 anti-RFP (Rockland, 600-401-379-RTU) in 5 ml of immunostaining buffer containing 3% donkey serum (Jackson ImmunoResearch, 017-000-121), 10% CHAPS (BioChemica, A1099), 10% dimethyl sulfoxide (DMSO), 1% glycine (Sigma-Aldrich, G7126) and 1% CD5 (Santa Cruz; sc-215141B) in 0.1× PBS for 7 days at 37 °C on a rotating wheel. After 3 days of washing, 1:400 of donkey anti-rabbit–Cy3 antibody (Jackson ImmunoResearch, 711-165-152) and donkey anti-chicken–AlexaFluor594 antibody (Jackson Immuno Research, 703-585-155) in immunostaining buffer was added for 7 days at 37 °C on a rotating wheel. Brains were washed extensively, dehydrated to 100% methanol in steps and then cleared overnight in BABB (high-purity solvents required; two parts benzyl benzoate (Sigma-Aldrich, 8.18701) and one part benzyl alcohol (Sigma-Aldrich, 108006)). For colocalization of Tubb2a and GFP, we performed standard immunofluorescence^76^, with 1:250 anti-GFP (Aves, GFP-1020) and 1:300 anti-tubulin βII antibody (Abcam, ab179512/EPR16773). As secondary antibody, we used 1:500 Alexa Fluor 594 AffiniPure donkey anti-chicken IgY (IgG) (H + L) (Jackson ImmunoResearch, 703-585-155) and 1:500 goat anti-rabbit IgG (H + L) secondary antibody, DyLight 488 (Thermo Fisher, 35552).

### Immunoprecipitation of endogenously labeled Tubb2a derived from mouse brain tissue

Mice were injected with AAV-mTubb2a and AAV-Cas9 or ssAAV-8/2-hCMV-chI-EGFP-WPRE-SV40p(A)) as a control as described above, with the exception that the the AAV mixture was injected at eight locations (four locations per hemisphere) with 400 nl of AAV per injection site. After 21 days, mice were killed and brain halves were extracted and snap-frozen in liquid nitrogen. One brain half was used per immunoprecipitation reaction. Protein extraction was performed by placing thawed brain halves in 2-ml bead-beating tubes filled with 1.4-mm ceramic beads (Revvity, 19-627D) and 500 μl of 0.32 M sucrose containing immunoprecipitation lysis buffer (Pierce, 87787) and freshly added protease and phosphatase inhibitor cocktail (Thermo Fisher, 78440). Homogenization was achieved by shaking at 6 m s^−1^ for one cycle for 30 s using Bead Raptur Elite (Omni International, 19-042E). Brain lysates were incubated on ice for 5 min before being centrifuged at 500*g* for 5 min at 4 °C. The homogenized brain solution was carefully transferred to a fresh, prechilled 1.5-ml Eppendorf tube containing a layer of 1.2 M and a layer of 0.84 M sucrose lysis buffer solution. Gradient centrifugation was carried out at 21,000*g* for 60 min at 4 °C and deceleration set to 3 out of 10. The myelin-free fraction, which was found below the 0.84 M layer, was transferred to a fresh tube. An equal amount of lysis buffer was added, followed by a last centrifugation at 16,000*g* for 15 min at 4 °C. Then, 1% of input sample was taken from the clarified lysates. For immunoprecipitation of GFP or endogenous GFP-labeled Tubb2a protein, 25 μl of GFP-trap magnetic agarose beads (Proteintech, gtma) were used per reaction and incubated for 3 h at 4 °C on an overhead shaker. Resin-associated proteins were washed four times with lysis buffer and eluted with 4× Laemmli sample buffer (Bio-Rad, 1610747). Samples were loaded on 10% SDS polyacrylamide gels followed by western blotting on a PVDF membrane (Roth, T830.1). Blots were stained with Ponceau S (Roth, 5938.1) and blocked in 2.5% BSA for 1 h at room temperature. Incubation with primary antibody was performed overnight at 4 °C using anti-GFP (Thermo Fisher, MA5-15256; 1:1,000) followed by a 1.5-h incubation at room temperature with secondary antibody anti-horseradish peroxidase (Sigma-Aldrich, A8924, 1:5,000). The enhanced chemiluminescence detection system (Thermo Fisher, 32209) was used to visualize proteins using the Vilber Fusion FX machine.

### Imaging methods

For stereomicroscopy imaging, a SteREO Discovery.V8 from Zeiss and Zen2011 Blue Edition was used. In toto cleared *X.* *tropicalis* embryos and mouse brains were imaged using mesoSPIM^75,77^. For all mesoSPIM recordings, fluorophores were excited with the appropriate laser lines and a quadband emission filter (ZET405/488/561/640, Chroma) was used. Imaging was performed using dibenzyl ether as the immersion medium. Two-photon imaging was performed using a custom-built system with a reflective multi-immersion Schmidt objective^78^. A femtosecond Ti:sapphire laser (Chameleon Ultra II, Coherent) tuned to 980 nm provided excitation. A 720-nm short-pass filter (ET720SP, AHF) placed in front of the photomultiplier tube blocked excitation light and custom interchangeable filter cubes were used to select the GFP emission channel. Live time-lapse imaging for *Xenopus* embryos was performed on a widefield Thunder imager (Leica) and on a LSM980 Airyscan 2 (Zeiss). For widefield epifluorescence, a Leica DMi8 with a Leica K3M camera using a widefield light-emitting diode was applied for colocalization of Tubb2a and GFP. Stitching was performed with BigStitcher^79^. Data were rendered using Fiji^80^, Imaris (Oxford Instruments) or Napari (https://github.com/napari/napari)^81^. Segmentation was performed with U-Net^74,82^.

### DNA preparation, Sanger sequencing and NGS

Cells, *Xenopus* embryos or mouse AAV-injected hemispheres were lysed (50 mM Tris pH 8.8, 1 mM EDTA, 0.5% Tween-20, 2 mg ml^−1^ proteinase K) at 55 °C overnight. After proteinase K inactivation (10-min incubation at 98 °C), PCRs were performed using GoTaq G2 (Promega, M7845), Q5 (NEB, M0491L) or Phusion polymerase (ThermoFisher, F530S) (primers listed in Supplementary Table 1). For sequencing, amplicons were cleaned using nucleoSpin gel and PCR cleanup (Machery-Nagel, 740609) and sent for commercial sequencing (Microsynth). For NGS, amplicons were generated by PCR with appropriate adaptor sequences and commercially sequenced (INVIEW CRISPR Check (size: 450–500 bp, Illumina PE sequencing 2× 300 bp), Eurofins Genomics). Data analysis was performed using CRISPResso2 (ref. ^83^) and/or custom data processing.

### Pythia in silico modeling

The Pythia Python script is designed to simulate CRISPR–Cas-mediated gene-editing efficiencies using a given wild-type and mutant DNA sequence. It iteratively constructs potential editing templates by varying the lengths of the left and right homology arms and uses the inDelphi tool to predict repair outcomes and their frequencies. The results, including the predicted repair outcomes and their corresponding frequencies, are stored and reported to identify the most effective repair template for achieving the desired genomic modification.

We modeled the optimal ssODN repair template length, with the maximal Pythia score, across clinically relevant point mutations in *RPE65*, involved in retinitis pigmentosa and Leber congenital amaurosis, among others. For this, we obtained all *RPE65* ClinVar (accessed at January 6, 2024) single-nucleotide missense variants. For each missense variant, we calculated the minimal number of base changes required to change the codon usage from the human missense variant amino acid toward the restoration of the wild-type amino acid at that location. Next, Pythia code was used to compute the optimal ssODN repair template with the maximal Pythia score to establish this base point mutation, thus reverting the clinically relevant mutation at the amino acid level.

### Pythia editing in vitro

Potential ssODN repair templates were designed for three independent GFP gRNAs to establish two point mutations to convert eGFP to eBFP. Pythia scores were calculated with repair arm length set at 1 to 24, both left and right. From these, we performed a binning from 0 to 100 across the scores and randomly selected 30 repair templates for each gRNA, selecting three repair templates per decile bin and, thus, 90 in total. ssODN repair templates were ordered as desalted nonmodified primers from Microsynth (Supplementary Table 6). HEK293T-*AAVS1*(CMV:eGFP), featuring a stable one-copy integration of a pCMV:eGFP construct, was seeded at a density of 10,000 cells in a 96-well plate in 150 μl of standard DMEM. Then, 24 h later, cells were transfected using Lipofectamine CRISPRMAX (Thermo Fisher, CMAX00003) and Lipofectamine 3000 (Thermo Fisher, L3000001). gRNA was assembled using Alt-R CRISPR–Cas9 IDT crRNA and Alt-R CRISPR–Cas9 tracrRNA, according to the manufacturer’s instructions, by heating it to 95 °C and cooling it to room temperature, yielding a duplex at a final concentration of 1 μM. RNP was assembled by incubation for 5 min at room temperature of 1 μM gRNA duplex, 250 ng of Cas9 protein (Alt-R S.p. Cas9 Nuclease V3, IDT) and 0.6 μl of Cas9 PLUS reagent (from CRISPRMAX kit). Transfection complexes for RNP were generated by incubation at room temperature for 20 min of 25 μl of RNP repair template, 1.2 μl of CRISPRMAX transfection reagent and 23.8 μl of Opti-MEM medium. Transfection complexes for ssODN were generated using Lipofectamine 3000 (Thermo Fisher, L3000001) according to the manufacturer’s instructions. In brief, 1 μl of 20 nmol ml^−1^ of ssODN repair template was packaged in a final volume of 10 μl. Both RNP transfection (50 µl final per well) and ssODN transfection (10 µl final per well) reagents were added to the 96-well plate. On the next day (approximately 20 h later), the medium was refreshed and cells were split and maintained according to standard HEK293T principles until analysis using flow cytometry at day 18.

### Pythia editing in vivo in *Xenopus*

A gRNA targeting the *X.* *tropicalis* gene *tyr* was designed and the Pythia software was deployed to identify the optimal repair template to generate a double point mutation. Repair template was ordered as desalted ssODN from Microsynth. gRNA was assembled using Alt-R CRISPR–Cas9 IDT as described above for *Xenopus*. For RNP assembly, 3 μl of Cas9 protein (1 μg μl^−1^, PNABio CP01) was mixed with 1 μl of gRNA and incubated at 37 °C for 5 min. Next, 1 µl of ssODN repair template (5 µM stock, 1 µM final concentration) was added. Embryos were microinjected in the one-cell stage immediately after cortical rotation, targeting the gray sperm entry point with 5–10 nl of injection mix. Restriction digests of PCR products were performed with BsrDI (NEB, R0574S) overnight at 37 °C in NEB buffer r2.1 and with BtsI-v2 (NEB, R0667S) overnight at 37 °C in NEB rCutSmart.

For viability testing, RNP was assembled as described above and mixed with ssODN templates (35 bp, 48 bp and 66 bp, 1 µM final concentration) and TRITC–dextran (0.5 ng μl^−1^, Sigma-Aldrich). Then, 16 h after injections, embryos were sorted for TRITC^+^ fluorescence, followed by live–dead sorting.

To compare Pythia scores to editing outcomes in the generation of a point mutation, the Pythia software was used to identify the optimal repair template for a new locus on the *tyr* gene. Two repair templates of decreasing length and Pythia score and two templates of increasing length but decreasing Pythia score were designed. Injections were performed as described above but with a fixed DNA concentration of 10.8 ng μl^−1^ (below the identified toxicity limit). The experiment was split into two injection rounds with a different mating pair for each. The optimal repair template and the two shorter-than-optimal templates or the two longer-than-optimal templates were injected per injected round. After 40 h, the embryos were pooled into groups of 75 per condition and lysed (50 mM Tris pH 8.8, 1 mM EDTA, 0.5% Tween-20 and 2 mg ml^−1^ proteinase K) at 55 °C overnight, followed by 10 min of heat inactivation at 98 °C. After centrifugation, DNA in the aqueous middle phase was PCR-amplified using Phusion polymerase (NEB, M0530L) with overhang primers containing NGS adaptor sequences (Supplementary Table 1). The product was purified (Macherey Nagel, 740609) and analyzed by NGS (INVIEW CRISPR Check (size: 450–500 bp), Eurofins Genomics) using CRISPR-GRANT^84^.

### Statistics and reproducibility

Statistical analyses are described in detail throughout the manuscript. For *Xenopus*, stereomicroscopy and mesoSPIM light-sheet imaging were performed on multiple embryos obtained from injected clutches or natural matings. The images shown are representative examples that reflect the consistent expression patterns as observed across positive embryos (efficiencies reported throughout the manuscript) of the same reporter genotype or injection condition. These imaging experiments were designed to qualitatively assess spatial expression of tagged proteins or reporter constructs and no statistical analysis was applied.

For the mouse experiments, mesoSPIM light-sheet imaging was performed on a single injected brain hemisphere. These datasets provide near full-tissue views that are representative of outcomes observed in our injection. Histological immunofluorescence staining of gene-edited mouse brains was performed on serial sections from individual animals. The images presented are representative of expression patterns reproducibly observed across multiple sections and animals processed under the same conditions. These experiments were intended to provide qualitative spatial validation rather than quantitative comparisons.

Immunoprecipitation of endogenously tagged Myh9, Ncam1 and Acta2 in *Xenopus* was performed once using pooled lysates from 2–5 representative embryos per condition. Western blotting and GFP immunoprecipitation from mouse brain tissue were carried out once, using lysate from a single brain hemisphere of one injected animal per condition. These experiments served as qualitative validations and were not designed for direct statistical comparison.

### Reporting summary

Further information on research design is available in the Nature Portfolio Reporting Summary linked to this article.

## Online content

Any methods, additional references, Nature Portfolio reporting summaries, source data, extended data, supplementary information, acknowledgements, peer review information; details of author contributions and competing interests; and statements of data and code availability are available at 10.1038/s41587-025-02771-0.

## Supplementary information

Supplementary Information Supplementary Figs. 1–17 (and raw uncropped source data).

Reporting Summary

Supplementary Video 1 Benchtop mesoSPIM recording of an adult kidney from an F_0_ *X.* *tropicalis* with a stable integration of *pax8*-CNS1:eGFP in the *h11* stable landing site.

Supplementary Video 2 Time-lapse imaging of tadpole development in a stable F_1_ *X.* *tropicalis* line with a stable i ntegration of *pax8*-CNS1:eGFP in the *h11* stable landing site.

Supplementary Video 3 Time-lapse imaging of tadpole development in a stable F_1_ *X.* *tropicalis* with a stable integration of CarAct:dsRed2 in the *h11* stable landing site.

Supplementary Video 4 Benchtop mesoSPIM and Schmidt objective two-photon microscopy recording of a stable F_2_ *X.* *tropicalis* with a stable integration of CarAct:dsRed2 in the *h11* stable landing site.

Supplementary Video 5 Time-lapse imaging of Myh9–BaoJin in F_0_ *Xenopus tropicalis* embryo epidermis, revealing intricate Myh9 protein dynamics by direct protein tagging.

Supplementary Video 6 Time-lapse imaging of Acta2–BaoJin in F_0_ *Xenopus tropicalis* embryo highlighting dynamics of Acta2-positive intestinal SMCs.

Supplementary Video 7 Benchtop mesoSPIM recording of wildDisco processed adult mouse brain demonstrating viral delivery and in-frame eGFP tagging of *Tubb2a*.

Supplementary Table 1–7 Supplementary Tables 1–7.

## Source data

Source Data Fig. 1 Unprocessed western blots and gels.

## Data Availability

The sequencing data generated in this study were deposited to the National Center for Biotechnology Information Sequence Read Archive under BioProject PRJNA1282594. Source data are provided with this paper.

## Code availability

The code used for analysis and implementation in this study is publicly available from GitHub (https://github.com/XenoThomasNaert/Pythia-Editing). The inDelphi model is available online (https://indelphi.giffordlab.mit.edu/ and https://github.com/maxwshen/inDelphi-model). The Pythia design tools can be accessed at (https://pythia-editing.org).
