# Supplementary material for: Precise, predictable genome integrations by deep-learning-assisted design of microhomology-based templates
Source: Nat Biotechnol. 2025 Aug 12;44(6):1023–36. doi: 10.1038/s41587-025-02771-0 (PMC13271894; doi:10.1038/s41587-025-02771-0)

nature biotechnology

Supplementary information https://doi.org/10.1038/s41587-025-02771-0

Precise, predictable genome integrations by deep-learning-assisted design of microhomology-based templates

In the format provided by the authors and unedited

Contents:

Supplementary Figures 2

Supplementary Figure 1: Computational modelling of predicted %repair by µH in relationship to a number and length of tandem repeats. 2

Supplementary Figure 2: Microhomology tandem repeat mediated integration in the *AAVS1* locus 4

Supplementary Figure 3: Targeted deep amplicon sequencing of genome-transgene boundary products. 6

Supplementary Figure 4: *TRAC* targeting in HEK293T Cells. 8

Supplementary Figure 5: Modelling the predicted percentage of repair via MMEJ based on the base-composition of the gRNA binding site in the genome reveals that base composition at position -4 increases the % of gene editing outcomes by MMEJ for gRNAs with PAM "NGG". 10

Supplementary Figure 6: Modelling the predicted percentage of repair via MMEJ based on the base-composition of the gRNA binding site in the genome reveals that base composition at position -4 increases the % of gene editing outcomes by MMEJ for gRNAs with PAM "NAA" 12

Supplementary figure 7: Correlating experimental on-target integration efficiencies to InDelphi predictions. 14

Supplementary figure 8: Generation and validation of tandem microhomology repair arm ssDNA repair templates for targeted gene integration.. 16

Supplementary figure 9: The *Xenopus tropicalis hipp11* locus can be gene edited by CRISPR/Cas9. 18

Supplementary figure 10: Stable integration into the *X. tropicalis hipp11* locus. 20

Supplementary figure 11: Stable integration into and tissue-specific expression from the *X. tropicalis hipp11* locus. 22

Supplementary figure 12: Computational framework and experimental strategies for genome-wide identification of optimal gRNAs and repair templates for transcript tagging in Xenopus tropicalis...... 24

Supplementary figure 13: Genome-wide analysis of last exon and intron length distributions in Xenopus tropicalis. 27

Supplementary figure 14: Validation of cassette Integration and repair outcomes at targeted genomic loci. 29

Supplementary Figure 15: Pythia Matrices for GFP gRNAs. 31

Supplementary Figure 16: Relationship between lengths of repair arms, Pythia scores and eGFP- to eBFP conversion efficiencies 32

Supplementary Figure 17: Pythia editing *in vivo* in *Xenopus tropicalis*. 34

Supplementary Figures

Supplementary Figure 1: Computational modelling of predicted %repair by µH in relationship to a number and length of tandem repeats. For 250,000 gRNAs binding the human genome, we modelled the expected editing outcomes when adding local sequence context left of the CRISPR/Cas9-mediated DSB to the right of the cut. Predicted %repair by µH is defined as any repair that mobilizes any of the available tandem repeats. This was computed using the InDelphi-HEK293T model (Top) and the InDelphi-mESC model (bottom). Box plots show the median, IQR, and whiskers extending to 1.5×IQR with circles representing outliers and *n* = 250,000. Colour scheme represents difference in number of tandem repeats used in the modelling.

Supplementary Figure 1:

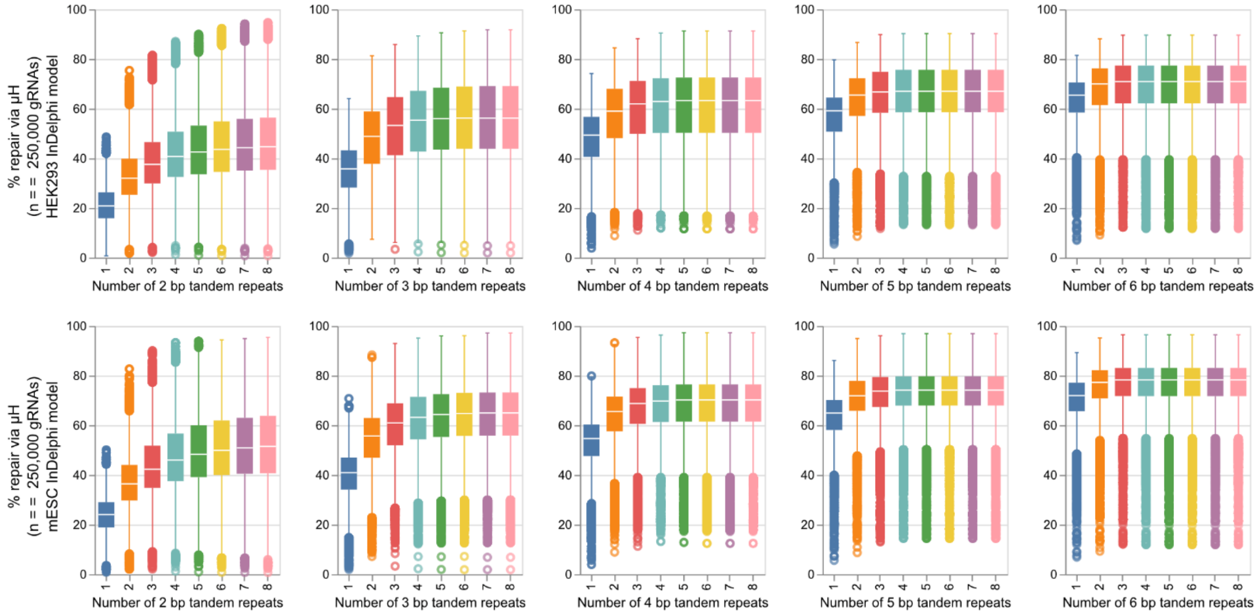

Supplementary Figure 2: Microhomology tandem repeat mediated integration in the *AAVS1* locus (a) Scheme of CRISPR/Cas9 integration strategy. The donor plasmid consists out of pCMV-eGFP expression cassette, flanked by 5x microhomology tandem repeat arms and inverted PaqCI restriction enzyme binding sites. dsDNA donor template is liberated by *in vitro* PaqCI digest, where this type II restriction enzyme provides a cut away from its recognition sequence, allowing fully customizable edges of the dsDNA donor template. This digest is co-delivered with *AAVS1* RNP into HEK293T cells. **(b)** PCR verification of targeted integration efficiency in GFP+ PaqCI+ single-cell clones. Left: Representative agarose gels demonstrating PCR amplification products corresponding to the expected sizes for the left and right integration junctions. Right panel: Pie charts summarizing PCR screening results of 154 GFP+ clones, showing that 25% (38/154) displayed the correct right junction product. Of these 38 positive clones, 100% also exhibited the correct left junction product, confirming precise on-target integration. **(c)** 5' (L) and 3' (R) junction products can only be amplified when co-delivering *AAVS1*, and not when co-delivering negative control RNP. **(d)** dsDNA repair templates as obtained after *in vitro* PaqCI digest of donor plasmids. This repair templates where co-delivered together with either negative control RNP or *AAVS1* RNP and contain either 4x microhomology tandem repeat repair arms or 0x microhomology tandem repeat repair arms (NHEJ).

Supplementary Figure 2:

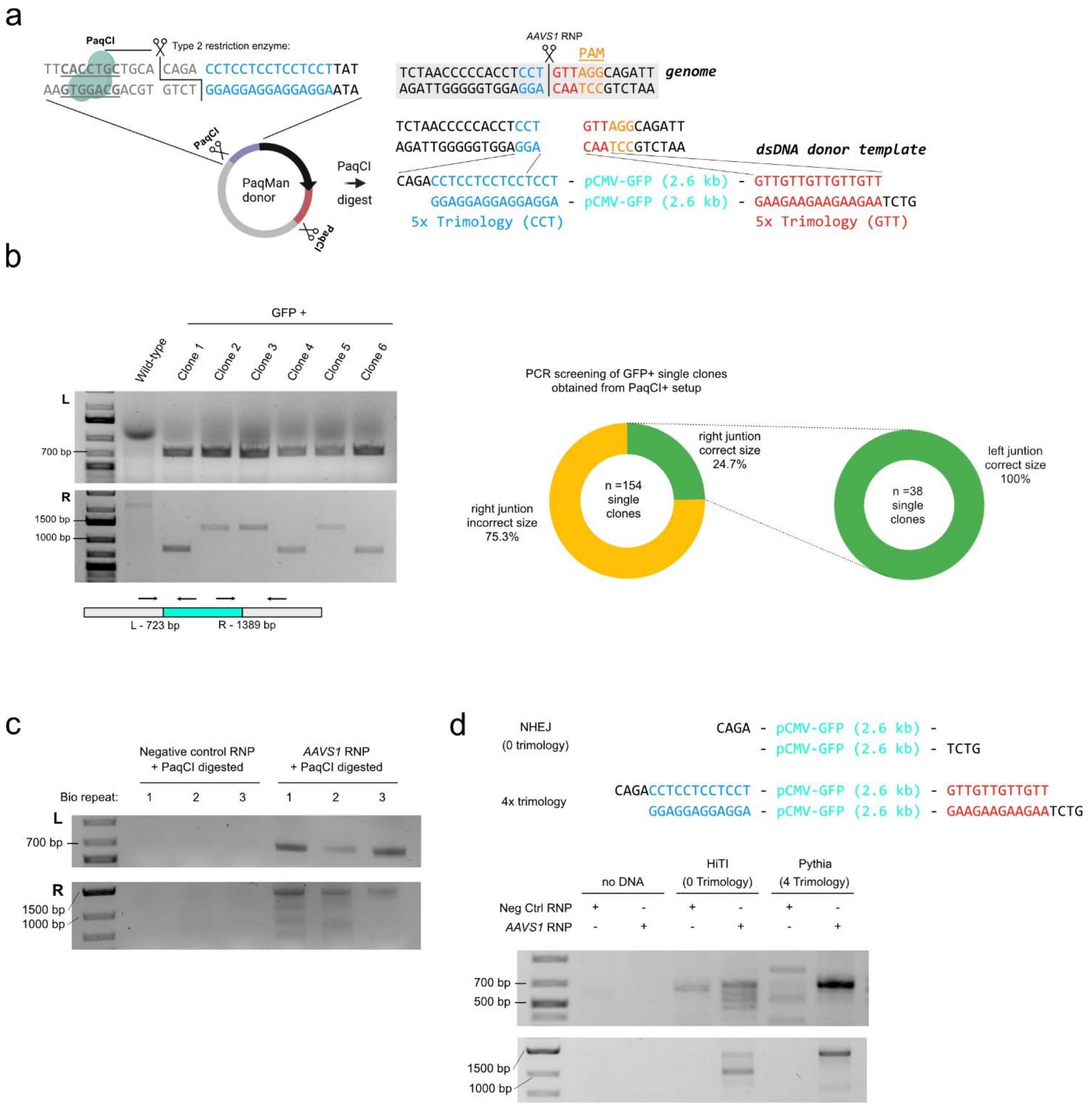

Supplementary Figure 3: Targeted deep amplicon sequencing of genome-transgene boundary products. (a) 5' (L) and 3' (R) junction products can only be amplified when co-delivering *AAVS1*, and not when co-delivering negative control RNP. Numbers 1 through 3 denote biological repeats (*n*=3). **(b)** CRISPResso2 analysis of next-generation sequencing of 5' (L) and 3' (R) junction products. Numbers indicate the shown biological repeat. **(c)** Visualisation of genome editing outcomes on both genome-transgene junctions demonstrating trimming both into the genome (1) and the transgene cassette (2). For each read in which no trimming into the genome or the cassette was observed, the number of deletions in the repair arms are shown. This data represents the average of the three independent biological repeats.

Supplementary Figure 3:

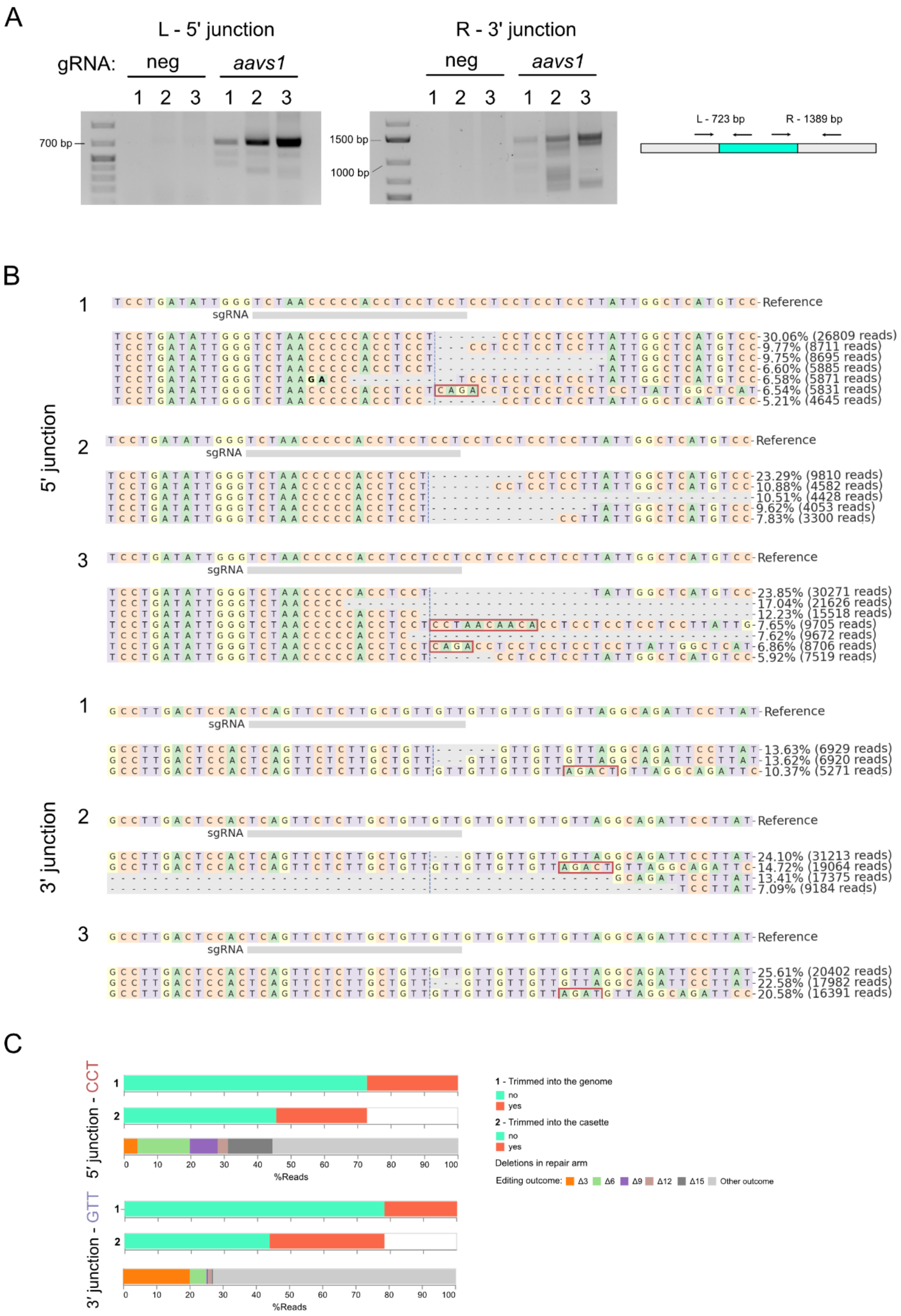

Supplementary Figure 4: *TRAC* targeting in HEK293T Cells. (a) Schematic representation of the CD19-CAR construct (from Glaser et al.; PMID: 37095570). **(b)** Strategy for targeting the first exon of *TRAC* in HEK293T cells using a ribonucleoprotein (RNP) complex, co-delivered with one of three repair templates: (i) a template with homology-directed repair (HDR) arms, (ii) a template without repair arms utilizing the HiTi (homology-independent targeted integration) approach, or (iii) a template with tandem repeat repair arms. **(c)** PCR-based strategy for amplifying 5′ junction products between *TRAC* and the CAR-T cassette. **(d)** Agarose gel electrophoresis showing on-target integration at *TRAC* exon 1 for three independent biological replicates, for all different repair arm designs of the CD19-CAR construct.

Supplementary Figure 4:

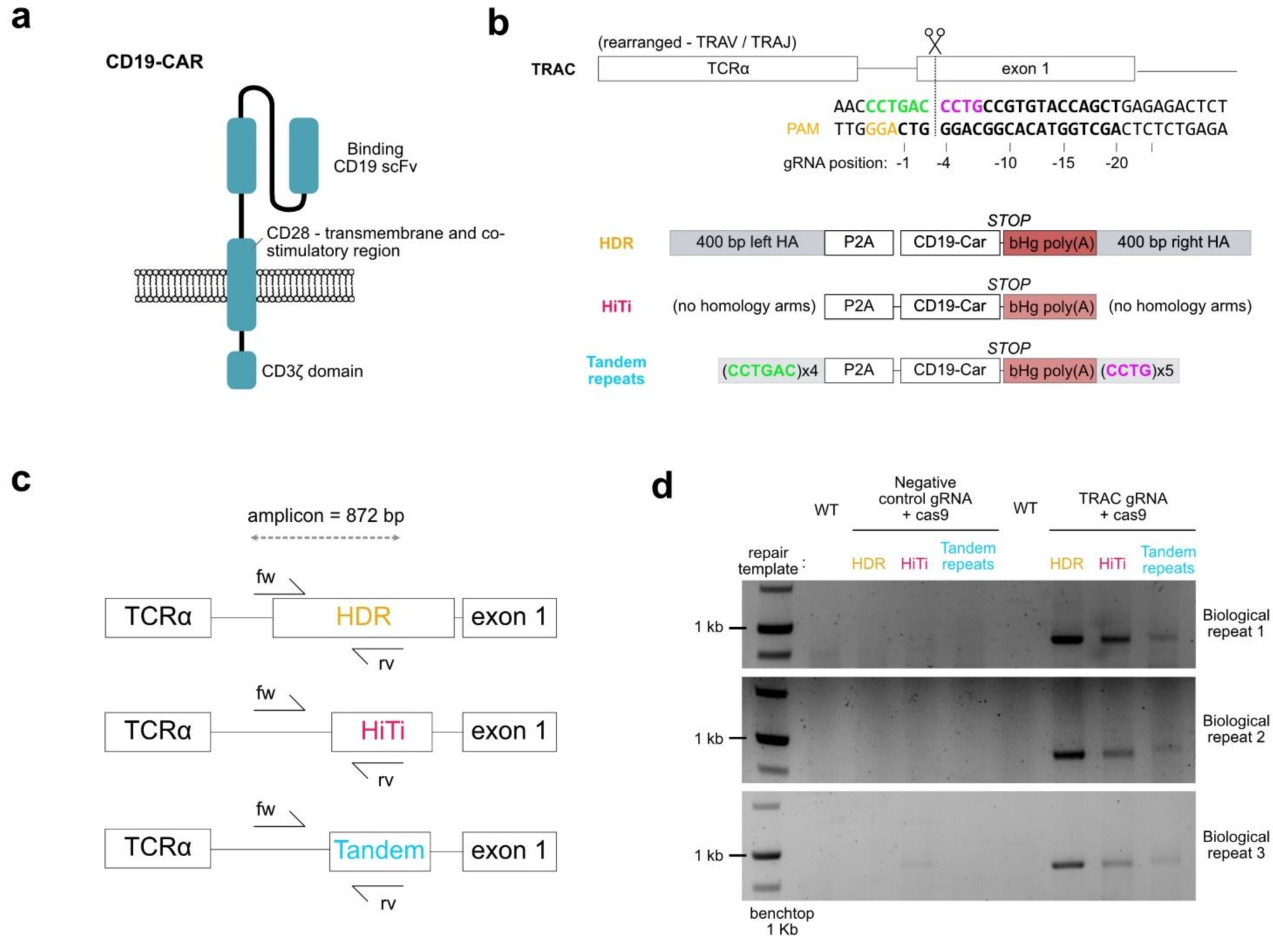

Supplementary Figure 5: Modelling the predicted percentage of repair via MMEJ based on the base-composition of the gRNA binding site in the genome reveals that base composition at position -4 increases the % of gene editing outcomes by MMEJ for gRNAs with PAM "NGG". We performed an exome-wide analysis across the human coding genome using the InDelphi-HEK293 model retrieving 10,813,171 unique gRNAs. This suggests an observable trend towards enrichment for expected % of gene editing outcomes by MMEJ when the base at position -4 is a C or G, when compared to A or T. Such enrichment is not apparent for the positions -5 through -9 (counting the NGG PAM as nucleotides 0-2). No formal statistical tests were applied due to the large dataset size (10,813,171 unique gRNAs). For visualization purposes, a random subset of 500,000 gRNAs was plotted; all analyses were conducted on the full dataset. Bars show the median, individual points not plotted due to large sample size.

Supplementary Figure 5:

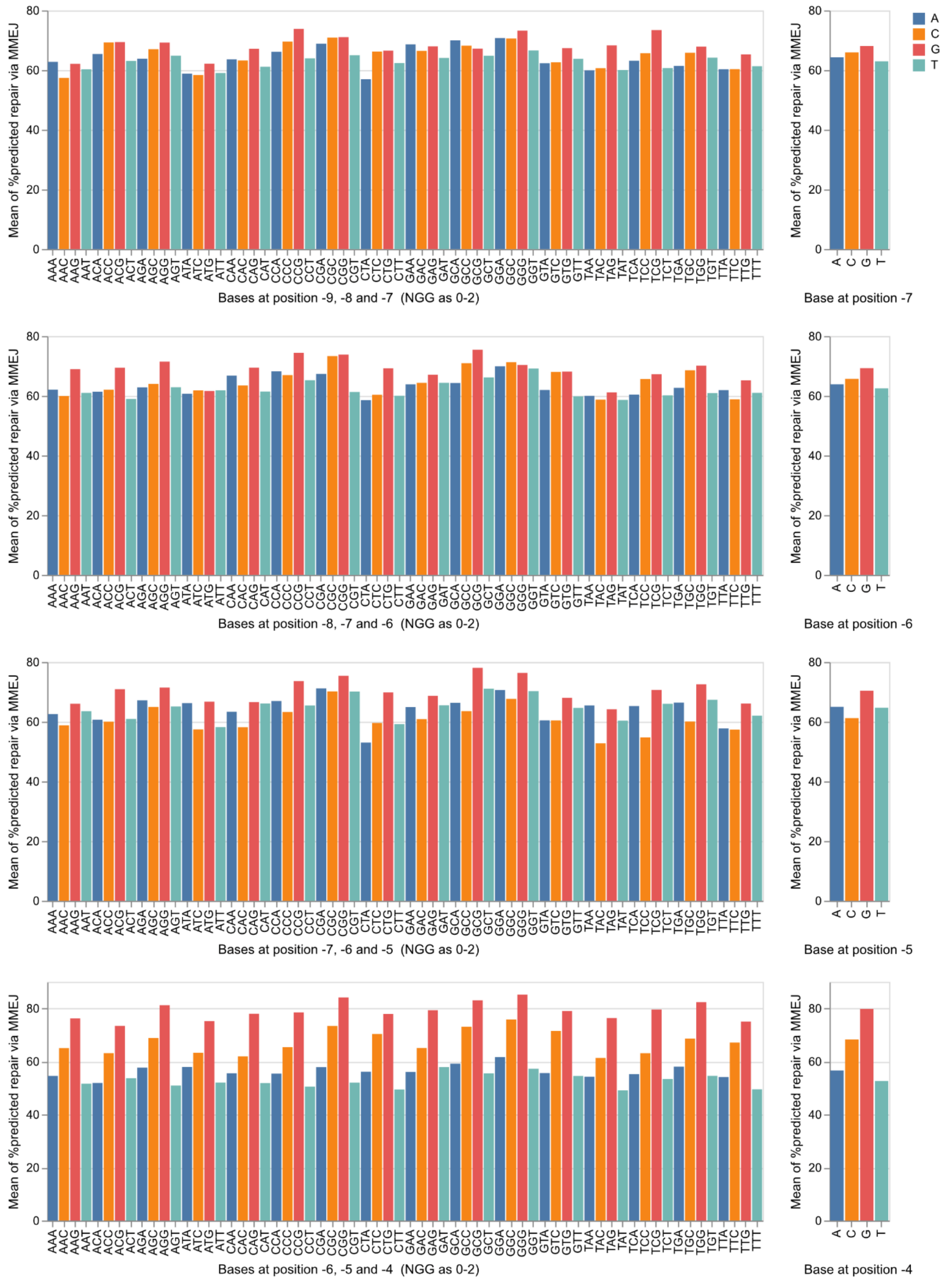

Supplementary Figure 6: Modelling the predicted percentage of repair via MMEJ based on the base-composition of the gRNA binding site in the genome reveals that base composition at position -4 increases the % of gene editing outcomes by MMEJ for gRNAs with PAM "NAA". We performed an analysis across a subset of the human coding exome using the InDelphi-HEK293 model retrieving 1,751,128 unique gRNAs. This suggests an observable trend towards enrichment for expected % of gene editing outcomes by MMEJ when the base at position -4 is a C or G, when compared to A or T. Such enrichment is not apparent for the positions -5 through -9 (counting the NGG PAM as nucleotides 0-2). No formal statistical tests were applied due to the large dataset size (10,813,171 unique gRNAs). For visualization purposes, a random subset of 500,000 gRNAs was plotted; all analyses were conducted on the full dataset. Bars show the median, individual points not plotted due to large sample size.

Supplementary Figure 6:

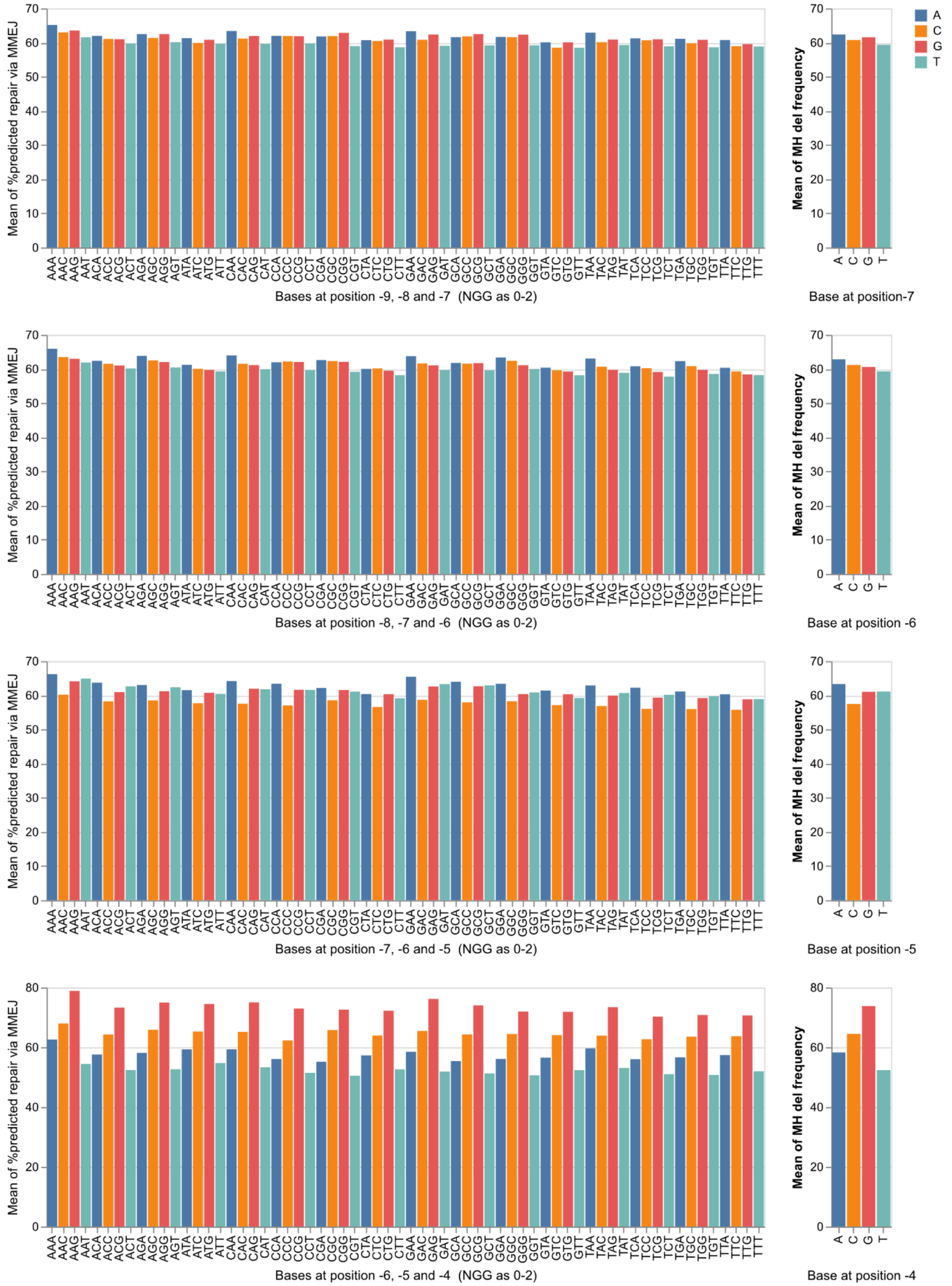

Supplementary figure 7: Correlating experimental on-target integration efficiencies to InDelphi predictions. (a) Scatter plot of InDelphi predicted % of repair by MMEJ at the endogenous locus (in the absence of exogenous DNA) and the on-target integration efficiency (normalized for random background integration). **(b)** There is an inverse correlation between the % of predicted perfect repair (defined as any repair mobilizing a microhomology tandem repeat) and the % of predicted +1 insertions. **(c-d)** As such, on-target integration efficiencies decrease when there is a higher % of prediction for +1 insertions and increase when there is a higher % of predicted perfect repair. Data points are means of three independent biological replicates; two-sided Pearson correlation was used. Statistical significance indicated (***; $p < 0.001$). $n = 32$ for each panel.

Supplementary figure 7:

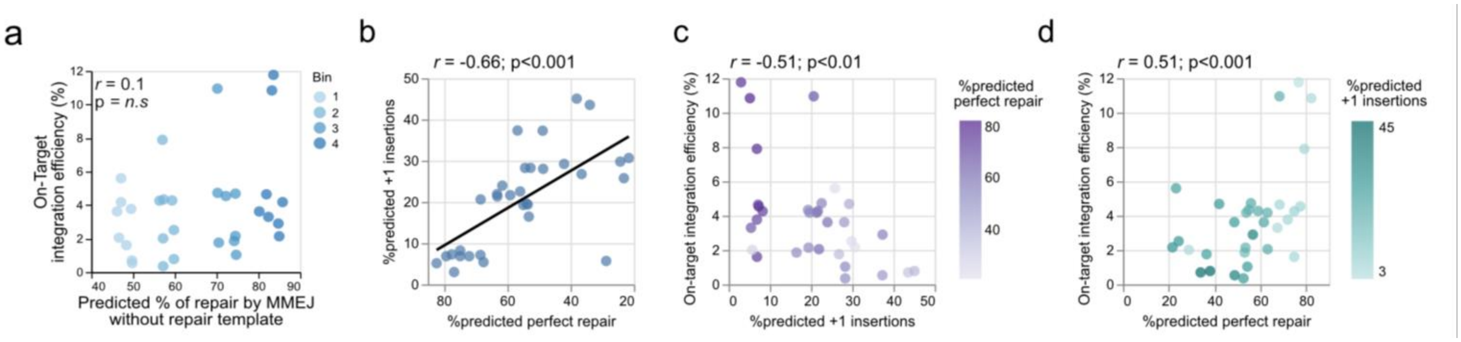

Supplementary figure 8: Generation and validation of tandem microhomology repair arm ssDNA repair templates for targeted gene integration. (a) Quality control of ssDNA production from dsDNA amplicons using the Takara Guide-it method. As per manufacturer's recommendations, successfully generated ssDNA migrates at approximately half the molecular weight of the corresponding dsDNA amplicons and shows reduced signal intensity due to decreased intercalation of Midori Green dye. RNA was synthesized once for this blot, and quality was assessed by agarose gel electrophoresis. **(b–d)** Integration efficiencies using ssDNA repair templates in tandem microhomology repeat-mediated gene targeting. **(b–c)** Integration efficiencies, **(b)** color-coded based on the targeted gene or **(c)** the orientation (sense vs. antisense) of the ssDNA repair template. Unpaired Independent two-tailed t-test, $n = 8$, $p = 1.35 \times 10^{-4}$. **(d)** Direct comparison reveals no significant difference in integration efficiency between sense and antisense-oriented ssDNA templates. Unpaired independent two-tailed Student's t-test, $n = 4$, $p = 0.719$ (n.s.). Statistical significance indicated (***; $p < 0.001$). Box plots show the median, IQR, and whiskers extending to 1.5×IQR.

Supplementary figure 8:

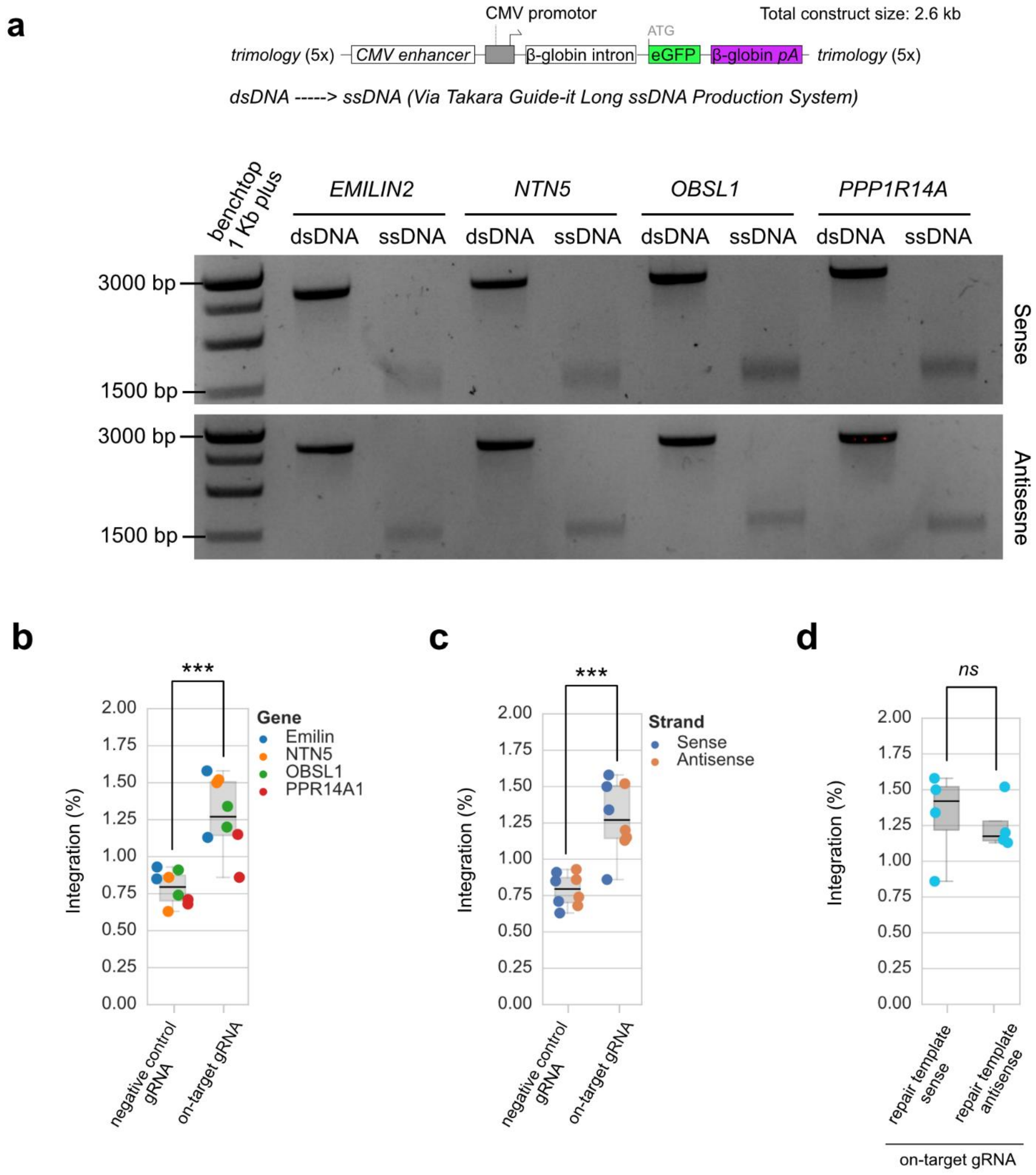

Supplementary figure 9: The *Xenopus tropicalis hipp11* locus can be gene edited by CRISPR/Cas9. (a) Using genome synteny (Genomicus[35]), we identify conservation of the intergenic region between *drg1* and *eif4enif1* corresponding to the *Hipp11* (*H11*) transgene landing site previously described in human, mouse and pig. Of note, synteny here is not conserved in the teleost lineage. **(b)** 55.3% (447 gRNAs/809) of potential gRNAs targeting this region have an unacceptable off-target profile with >1 perfect off-target match in the *Xenopus tropicalis* genome. **(c-e)** For the remaining gRNAs, we calculated via InDelphi-mESC the predicted frequency of editing outcomes via MMEJ, predicted frameshift repair frequency and the on-target CRISPRScan score to identify two suitable gRNA target sites (*h11-α* and *h11-β*) spaced 767bp apart. **(f)** One-cell stage *X. tropicalis* embryos were injected with CRISPR/Cas9 targeting either *h11-α* or *h11-β*. Embryos were grown until stage 23, lysed (pools of 3 to 5 embryos) and genome editing efficiencies are shown, as determined by sanger sequencing and trace deconvolution.

Supplementary figure 9:

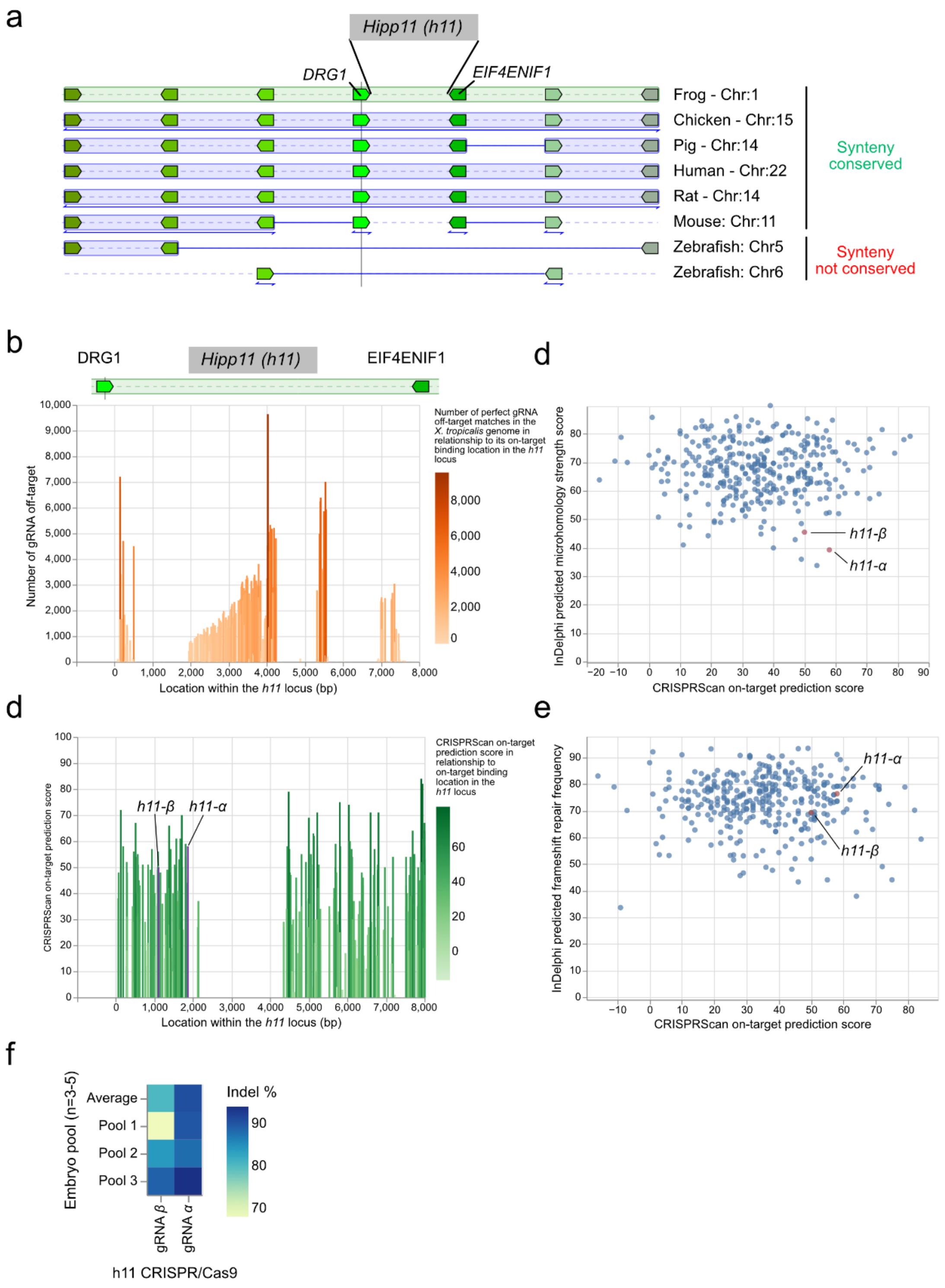

Supplementary figure 10: Stable integration into the *X. tropicalis hipp11* locus. (a) Schematic of the CRISPR/Cas9 integration strategy. Co-delivering two RNPs (*h11-α* and *h11-β*) together with dsDNA donor template leads to several potential gene editing outcomes. Firstly D1a, amplified are unedited and locally edited (Indels) genome copies. Secondly D1b, amplified in case of CRISPR-mediated deletion of intervening DNA between the binding site of *h11-α* and *h11-β*. Thirdly, integration of the dsDNA donor template, in place of the intervening DNA between the binding site of *h11-α* and *h11-β*. **(b)** Co-delivering *h11-α* and *h11-β* RNP results in a lower amplification of product D1a, and an increase in product D1b, showcasing CRISPR-mediated deletion as an editing outcome. All lanes are pools of 50 injected embryos. **(c)** 5' (L1, L2, L3) and 3' (R1) junction products can be amplified, revealing targeted integration of pCMV-eGFP in the *hipp11* locus. **(d)** Example of clonal expansion of GFP+ cells as an embryo develops from neurula to metamorphosis. **(e)** Several GFP+ tissues were dissected, lysed and the 3' (R1) junction products amplified, revealing stable long-term integration into the *hipp11* locus

Supplementary figure 10:

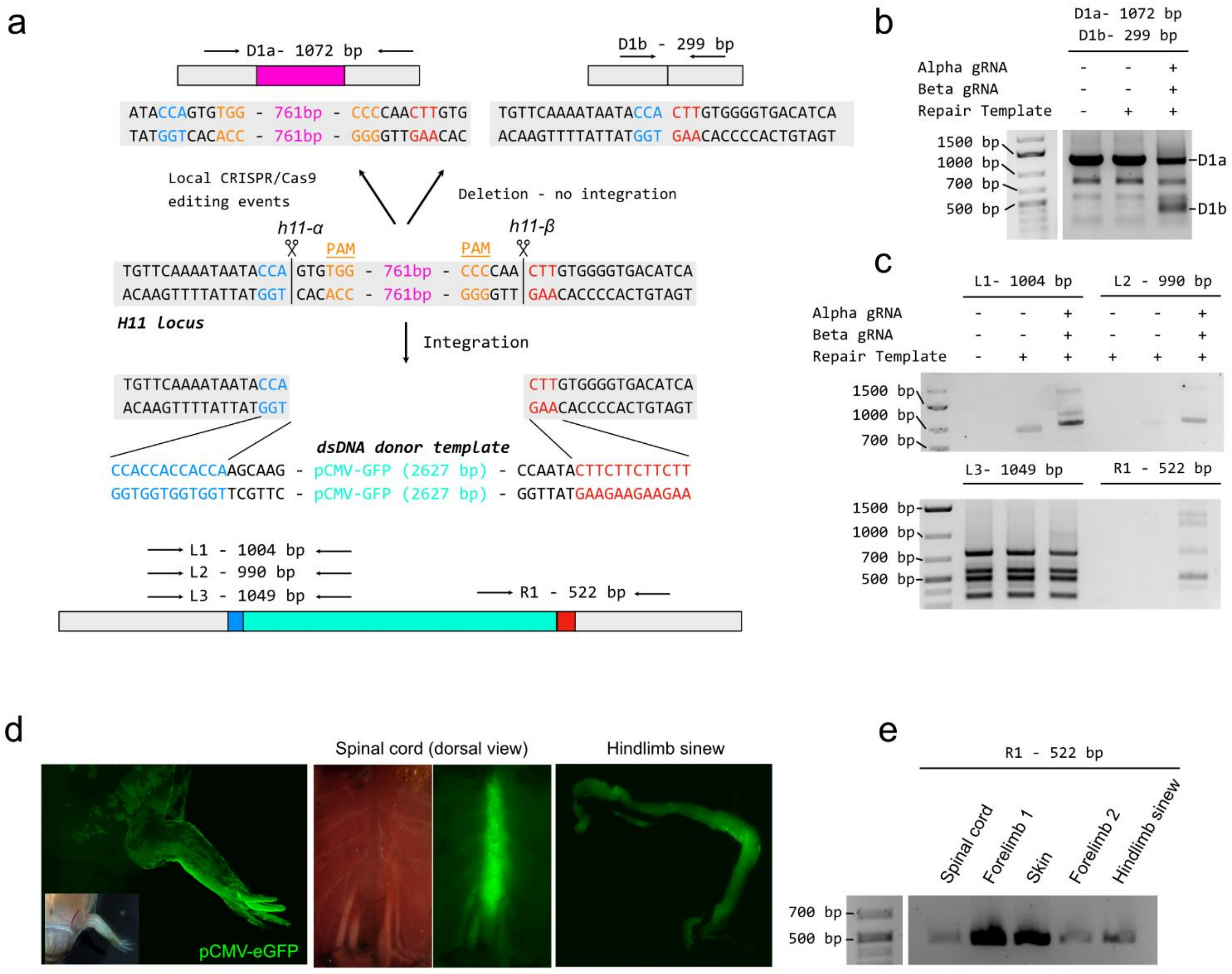

Supplementary figure 11: Stable integration into and tissue-specific expression from the *X. tropicalis hipp11* locus. (a) Schematic CRISPR/Cas9 integration strategy. Co-delivering two RNPs (*h11-α* and *h11-β*) together with *Pax8*-CNS1:eGFP dsDNA donor template (5x microhomology tandem repeats) generated by simple overhang PCRs from a plasmid **(b)** In F1 offspring, 3' (R) junction products were amplified, revealing targeted integration of *Pax8*-CNS1:eGFP in the *hipp11* locus and positive germline transmission. **(c)** In six F0 founder animals, three (50%) demonstrated transmission through germline at mean rate of 29.1% ± 8.6%. **(d)** Schematic CRISPR/Cas9 integration strategy. Delivering two RNPs (*h11-α*) together with CarAct:dsRed2 dsDNA donor template (8x microhomology tandem repeats) generated by simple overhang PCRs from a plasmid. **(e)** In F1 offspring, 3' (R) junction products were amplified, revealing targeted integration of *Pax8*-CNS1:eGFP in the *hipp11* locus and positive germline transmission. **(f)** F2 homozygote knock-in animals (stars) can be identified by a PCR with primers binding the genomic sites left and right of *h11-α*, thus amplifying the entire fragment (2738 bp) in homozygotes knock-in animals. This confirms single-copy integration of the construct.

Supplementary figure 11:

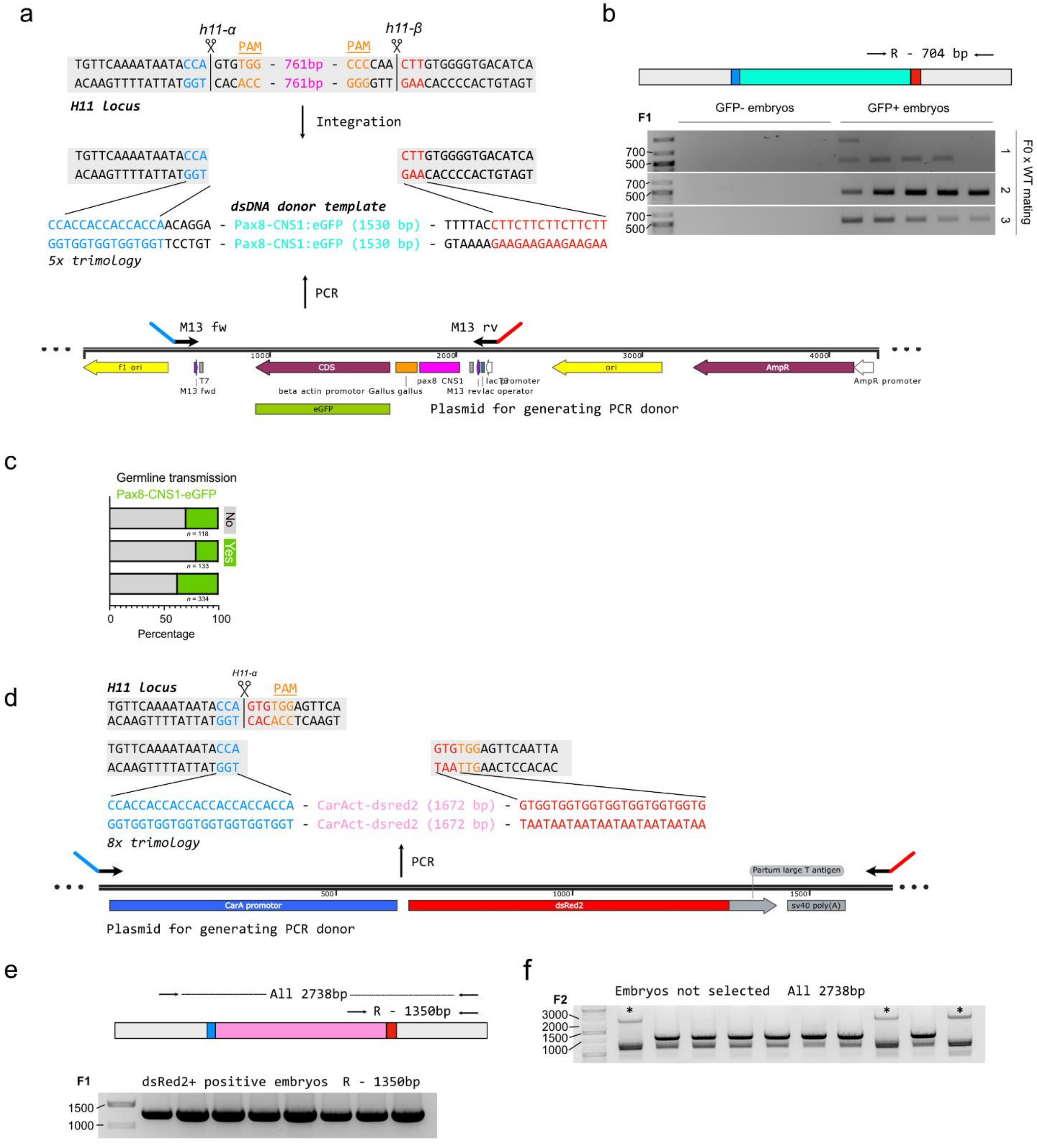

Supplementary figure 12: Computational framework and experimental strategies for genome-wide identification of optimal gRNAs and repair templates for transcript tagging in Xenopus tropicalis

(a) Schematic representation of the *Pythia* software suite and associated algorithms designed for predicting efficient genome-editing integration strategies. The software is freely accessible at pythia-editing.org.

(b) Outline of transcript tagging strategies used for labeling each *Xenopus tropicalis* transcript. Two distinct strategies were employed:

1. **Direct 3' exon tagging**: The DNA repair construct comprises the exonic sequence downstream of the CRISPR/Cas9-induced double-strand break (DSB), followed by a Glycine-Serine (GS) linker and enhanced GFP (eGFP).
2. **Exon replacement via intronic integration**: The repair construct contains a splice acceptor site, the complete last exon sequence, followed by a GS linker and eGFP. This construct integrates within the final intron of the targeted transcript.

(c) Computational pipeline for selecting and evaluating gRNAs:

For **direct 3' exon tagging (1)**, all potential gRNAs targeting the last exon of every *Xenopus tropicalis* transcript were identified genome-wide. Each gRNA's predicted on-target efficiency was scored using CRISPRscan. Integration efficiencies were calculated using the *Pythia* software based on varying repair arm lengths defined as:
Left repair arm length: $n \times i \ nucleotides$
Right repair arm length: $m \times j \ nucleotides$
Where:

$$(\mathbf{a})\ n, m = 3\ to\ 5, \quad i, j = 3 \qquad \text{or} \qquad (\mathbf{b})\ n, m = 3\ to\ 5, \quad i, j = 6$$

For the **exon replacement strategy (2)**, all potential gRNAs targeting the last intron genome-wide were initially identified and scored using CRISPRscan. Due to the extensive number of gRNAs present in intronic regions (which are generally longer than exons), computational efficiency was increased by retaining only those gRNAs with CRISPRscan scores >45. These candidates were further filtered using Cas-OFFinder to

exclude any gRNA with additional perfect off-target genomic matches. Integration scores were subsequently calculated using *Pythia*, employing longer repair arms defined as:

Left repair arm length: $n \times i\ nucleotides$

Right repair arm length: $m \times j\ nucleotides$

Where:

$$n, m = 3\ to\ 8, \qquad i, j = 3\ to\ 5$$

Subsequently, for each unique transcript ID, only the gRNA with the highest predicted on-target efficiency was retained. These top-performing gRNAs were then categorized into three quality classes based on their predicted efficiencies:

- **Low:** CRISPRscan score <40 and Integration score <65
- **Good:** CRISPRscan score 40–50 and Integration score 65–75
- **Very Good:** CRISPRscan score >50 and Integration score >75

(d) Comparative scatter plots illustrating the highest-scoring gRNA for each *Xenopus* transcript. Plots compare the distributions of gRNAs across the three defined efficiency classes (low, good, very good) for direct 3' exon tagging strategies using either 3 bp or 6 bp tandem repeats, as well as for the exon replacement strategy via intronic integration.

Supplementary figure 12:

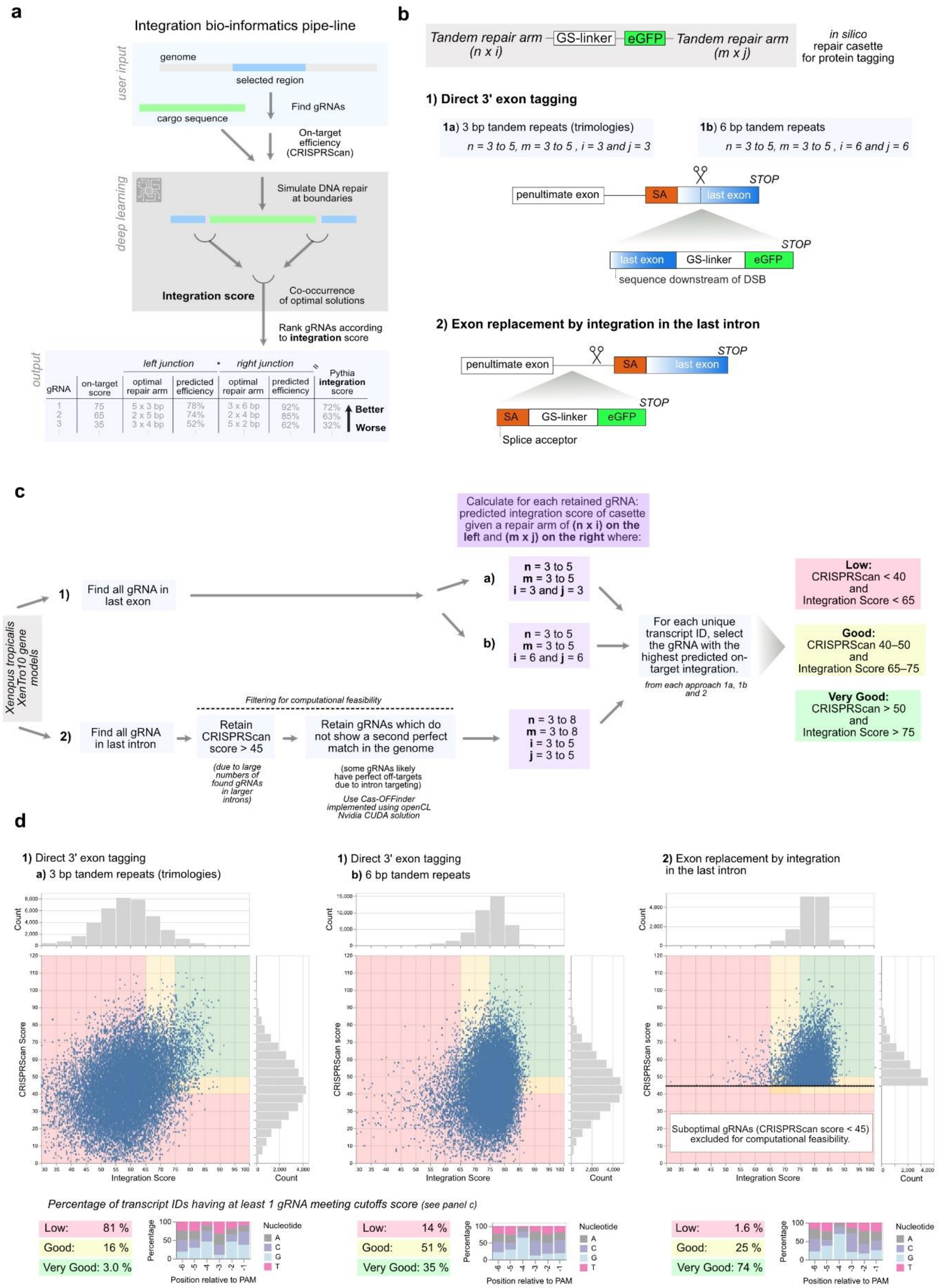

Supplementary figure 13: Genome-wide analysis of last exon and intron length distributions in Xenopus tropicalis. (a-b) Scatter plots showing the genome-wide distribution of last exon and intron lengths in *Xenopus tropicalis*. Mean lengths (± standard deviation) are 271 ± 538 bp for last exons and 2935 ± 9152 bp for last introns. The mean is shown as a broad dashed line, and the standard deviation (SD) as a fine dashed line. **(c)** the proportion of genes in which the last intron length exceeds the last exon length, compared to those where the last intron is shorter or equal in length.

Supplementary figure 13:

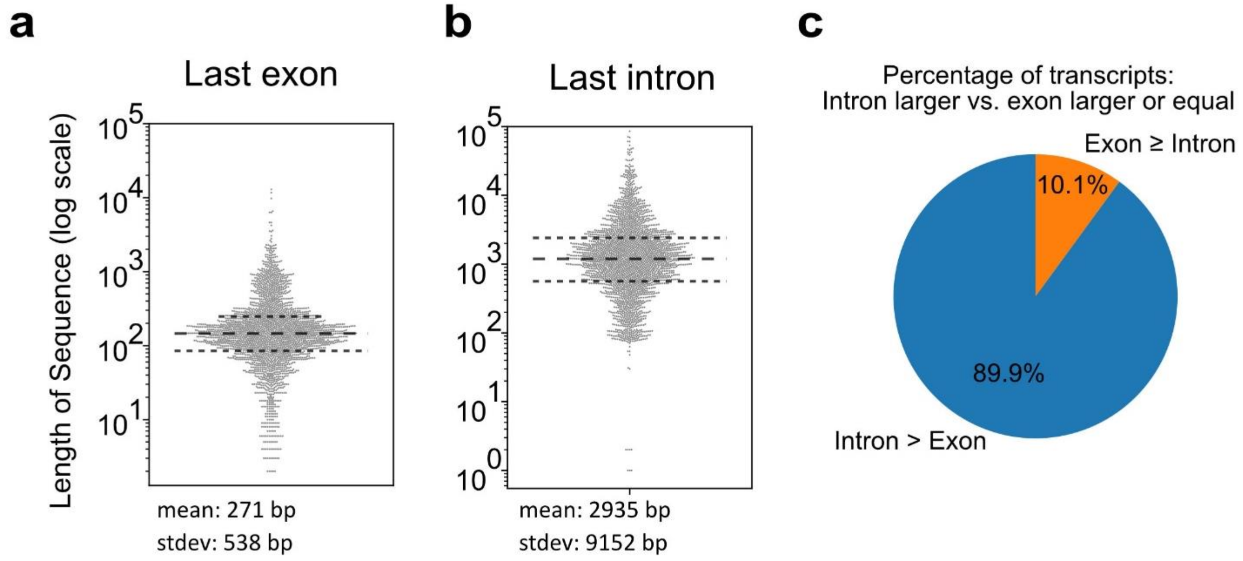

Supplementary figure 14: Validation of cassette Integration and repair outcomes at targeted genomic loci. (a) PCR products of 5’ boundary (genomic primer + cassette primer), 3’ boundary (cassette primer + genomic primer), and 5’-3’ (genomic primer + genomic primer, across insertion site). As expected, in wild type samples no products were amplified across cassette-genome borders. In mBaoJin positive samples, border products were present. Amplification with genome-genome primers across the insertion site resulted in a single short product for wild type DNA and in an additional long product in mBaoJin positive samples, corresponding to a single insertion of the tagging cassette. **(b)** Selection of different repair outcomes across the three insertion sites. Homology repeats in red, substituted bases in orange.

Supplementary figure 14:

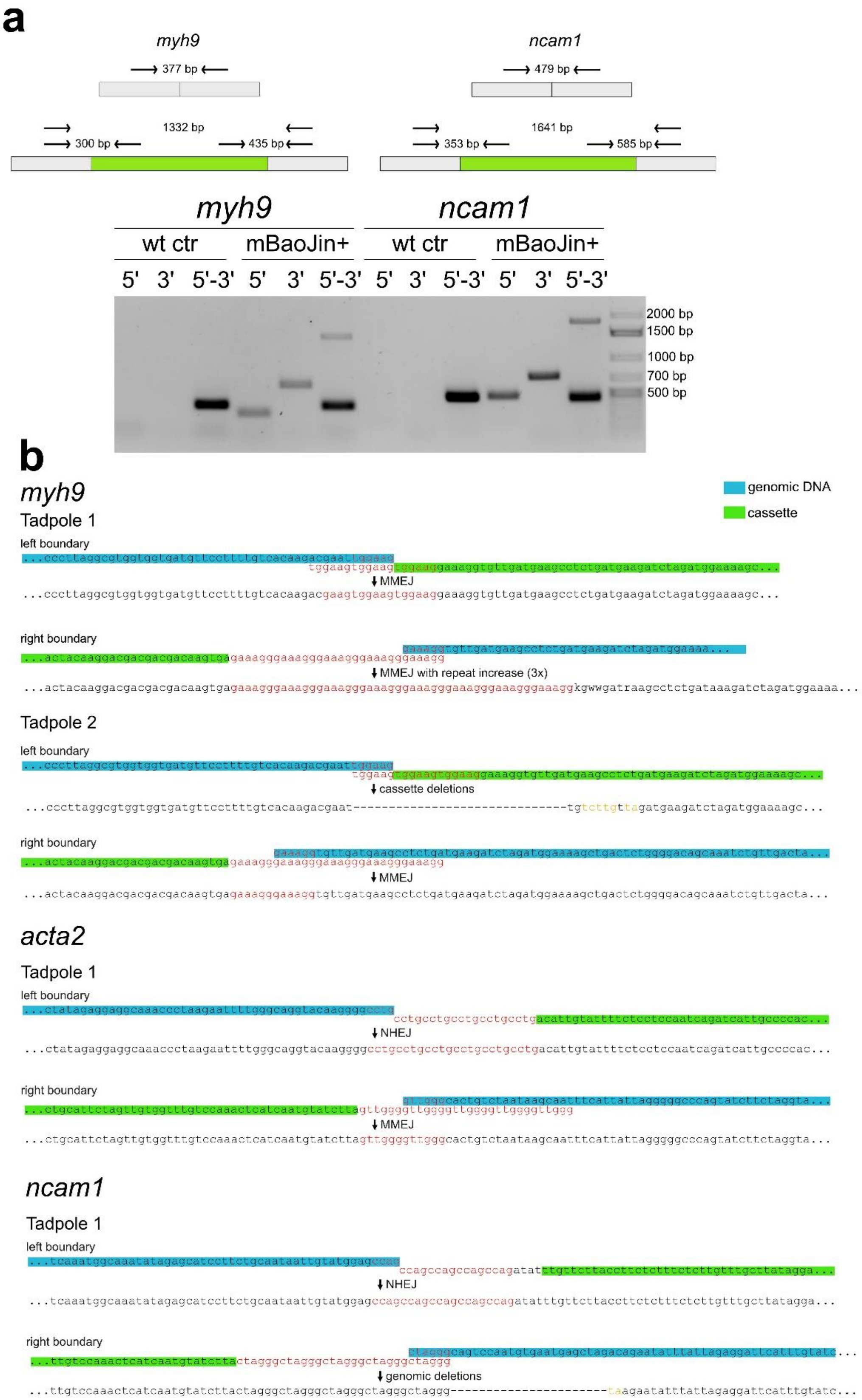

Supplementary Figure 15: Pythia Matrices for GFP gRNAs.

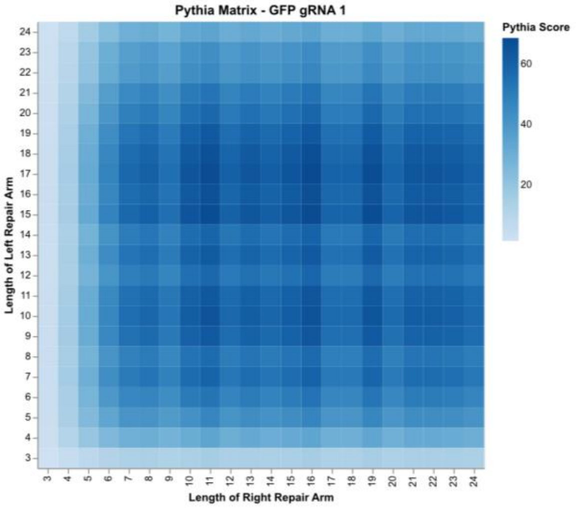

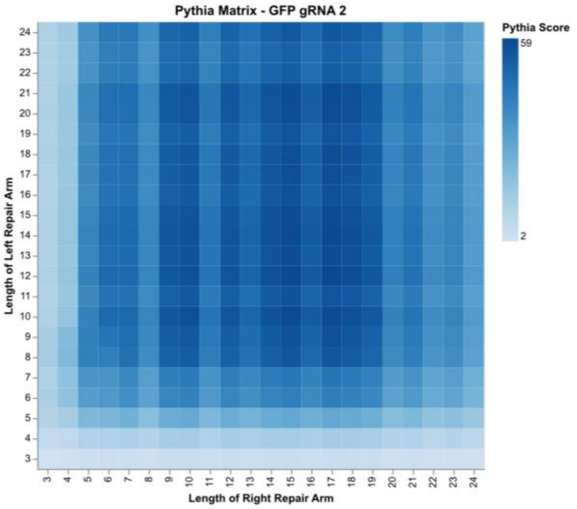

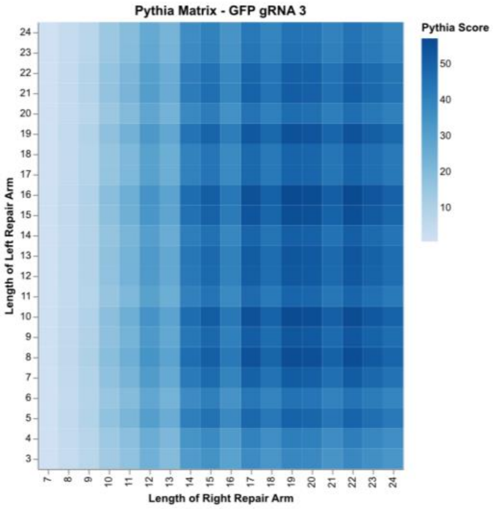

Supplementary Figure 16: Relationship between lengths of repair arms, Pythia scores and eGFP- to eBFP conversion efficiencies. Each row represents one GFP gRNA (top 1, middle 2, bottom 3) each with 30 distinct repair templates binned across Pythia prediction score (predicted %perfect repair). Both left and right repair arm length is related to conversion (eGFP- to eBFP) efficiencies. Further, there is a direct correlation between Pythia prediction scores and conversion efficiencies.

Supplementary Figure 16:

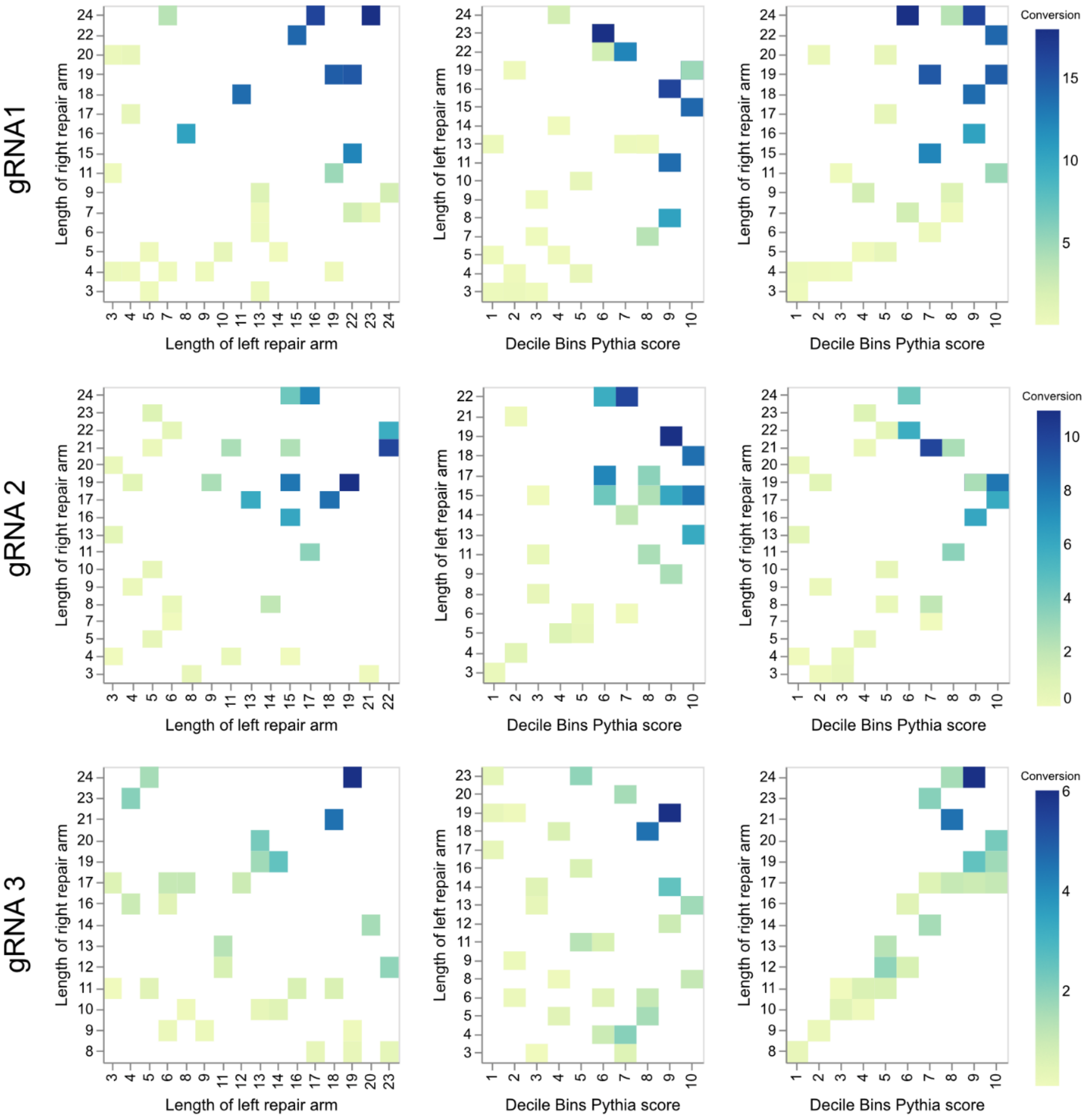

Supplementary Figure 17: Pythia editing *in vivo* in *Xenopus tropicalis*. (a) Schematic of experimental design to detect and quantify successful editing events. **(b)** Compared to non-injected animals, injected animals showed different levels of residual pigmentation (due to correct establishment of silent point mutations), which was binned across three classes **(c)** Frequency of phenotype occurrence grouped by class and evidence for gene editing in class 3 animals according to restriction enzyme analysis. >60% of animals showed evidence of gene editing (**d)** Schematic of the Pythia software suite and algorithms for designing editing strategies. The software is freely accessible at pythia-editing.org.

Supplementary Figure 17:

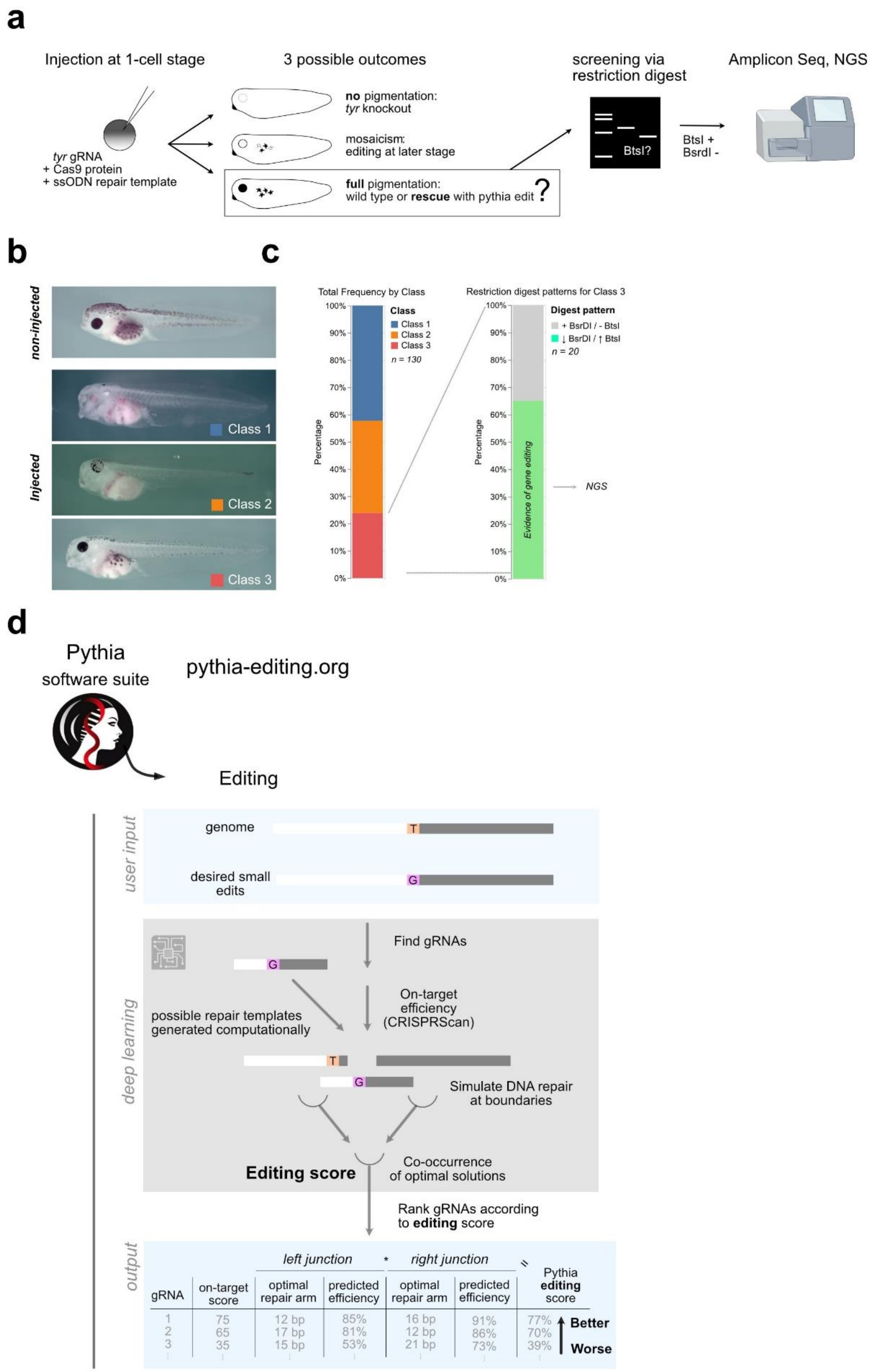

Uncropped scans of blots and gels in the supplementary.

Fig. S2b

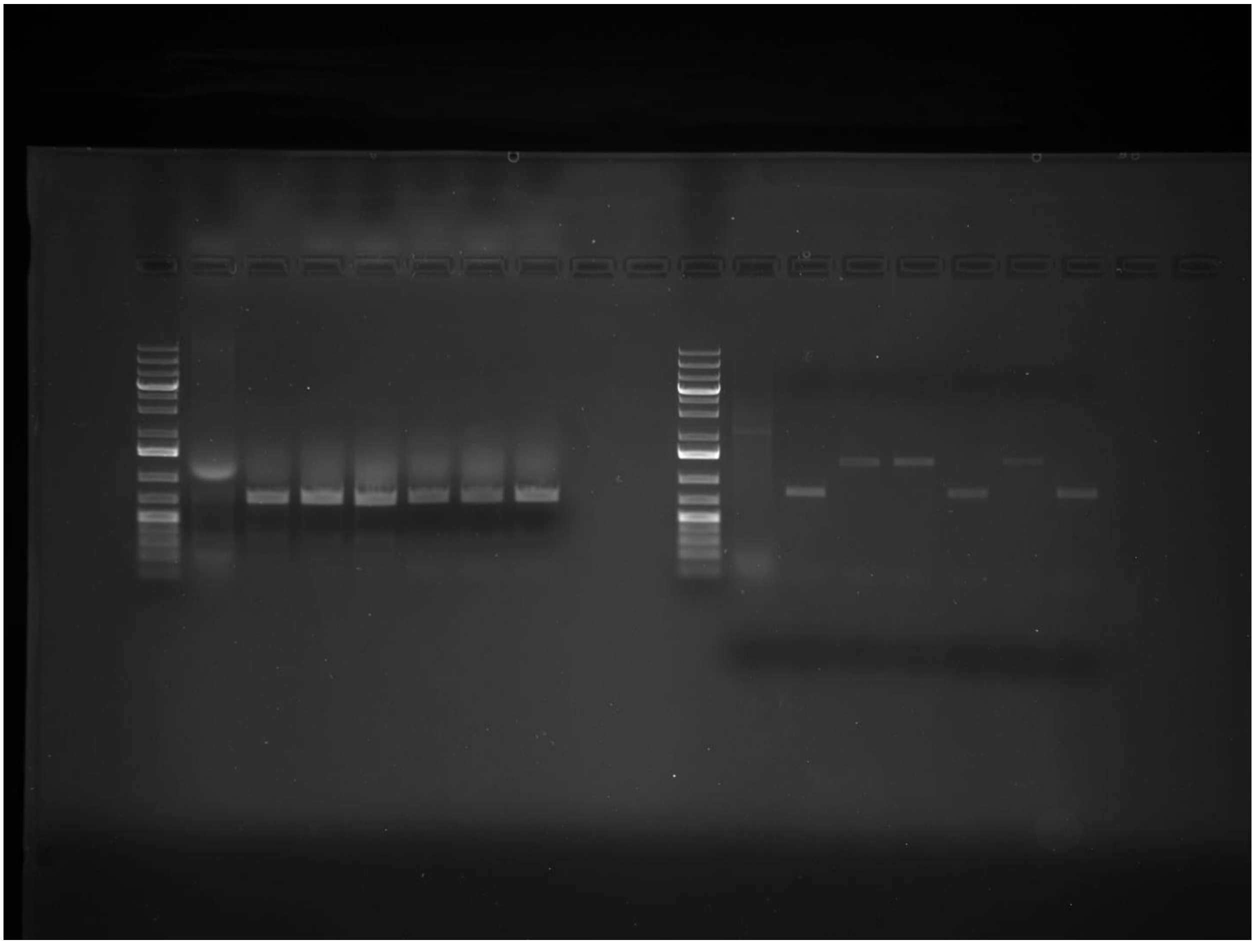

Fig. S2c

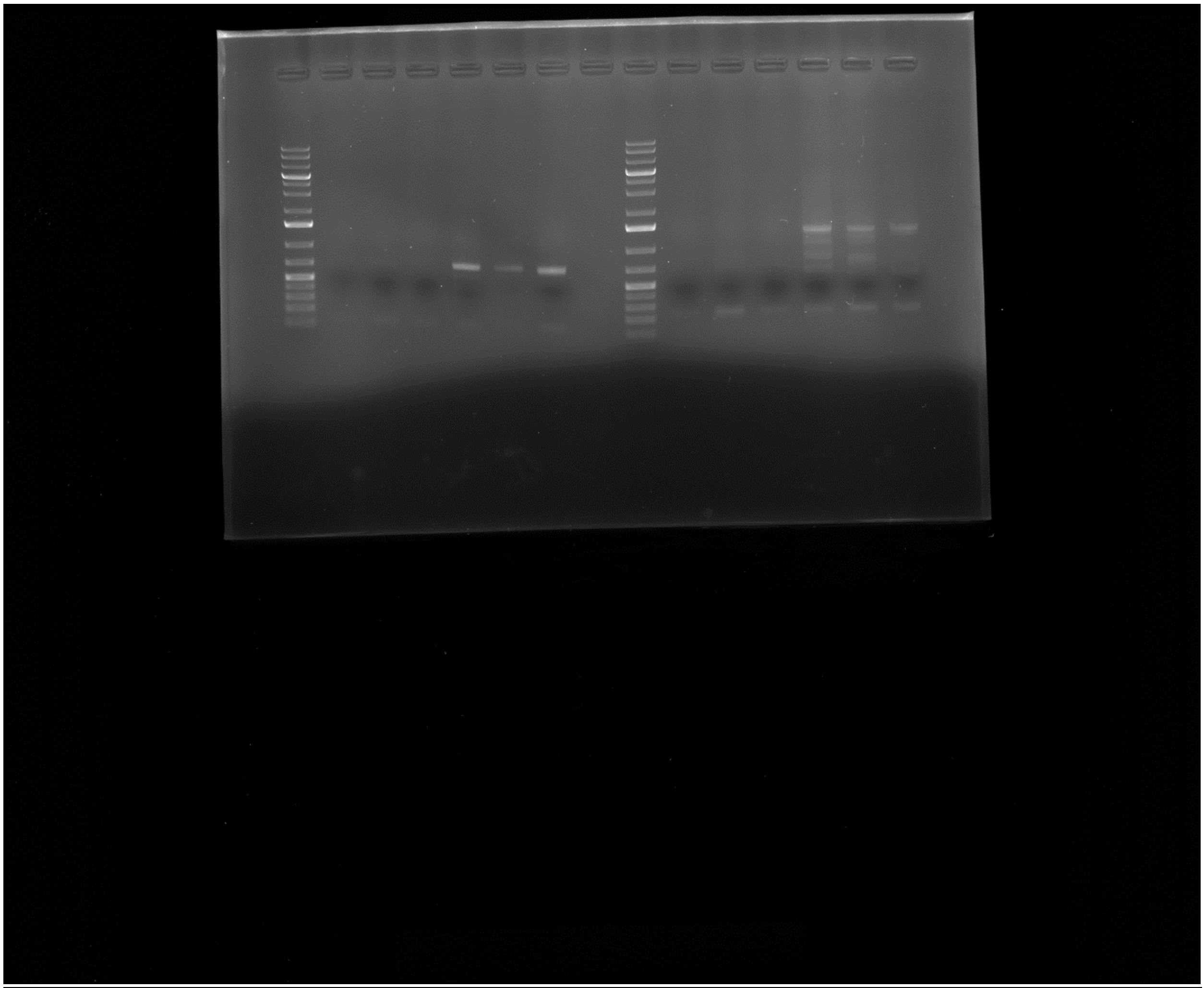

Fig. S2d - 1

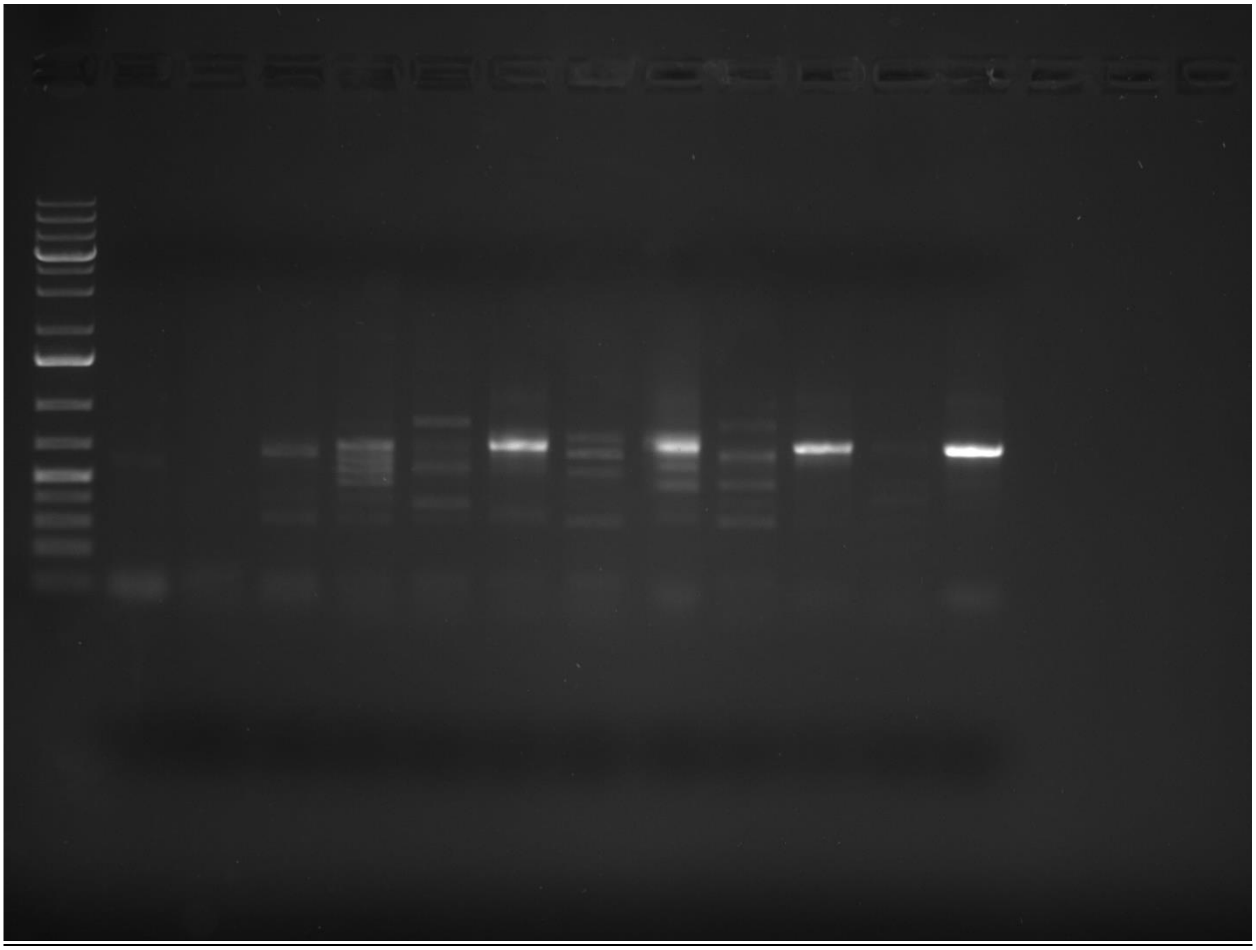

Fig. S2d – 2

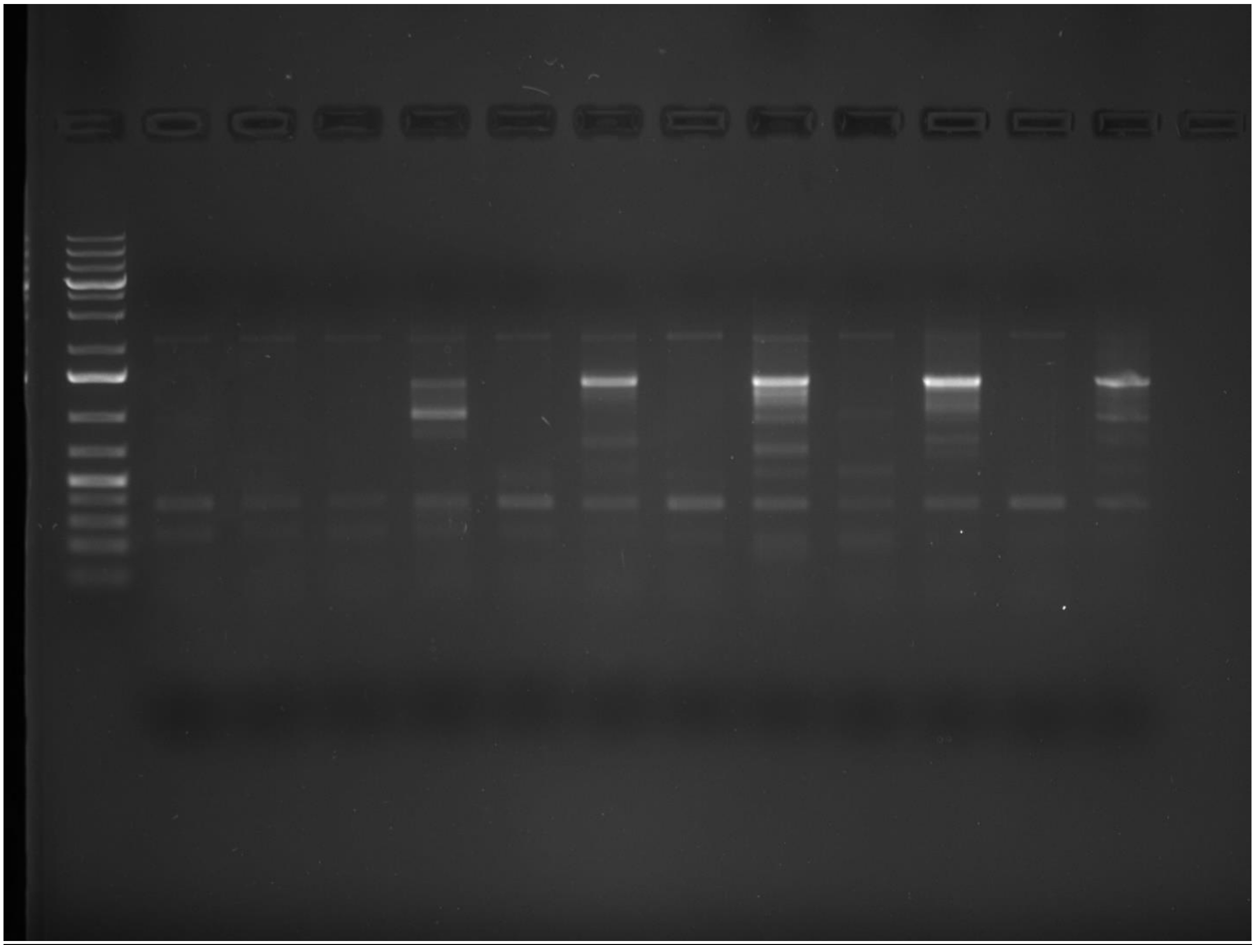

Fig. S3

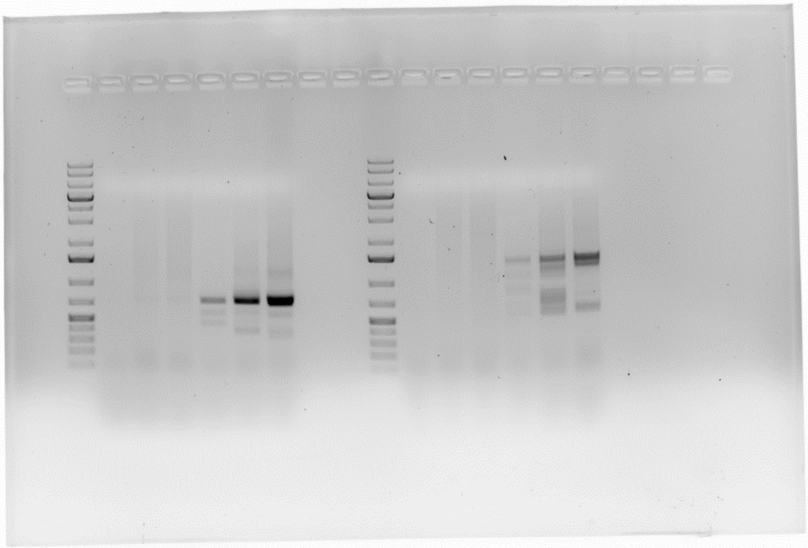

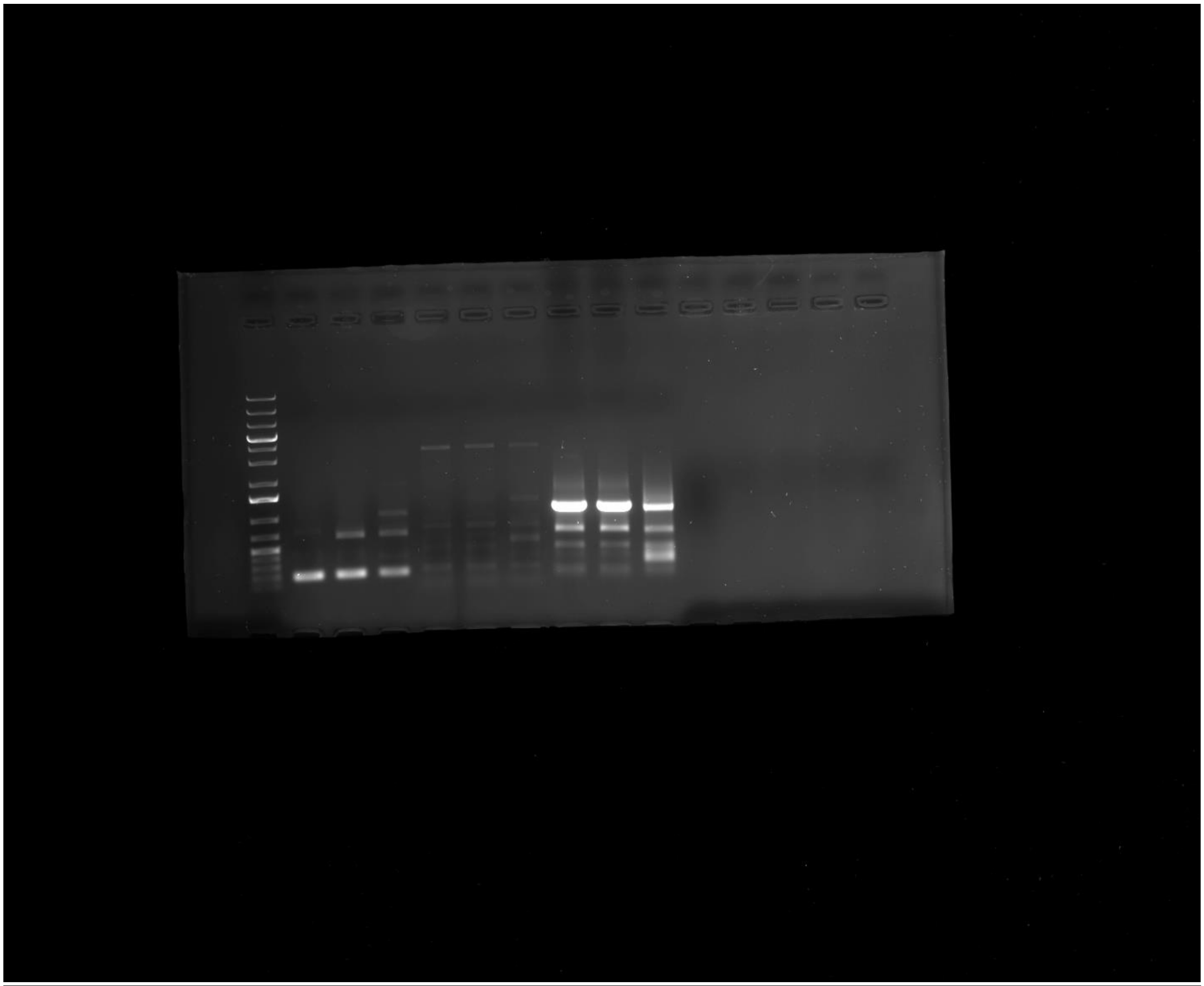

Fig. s4d – 1

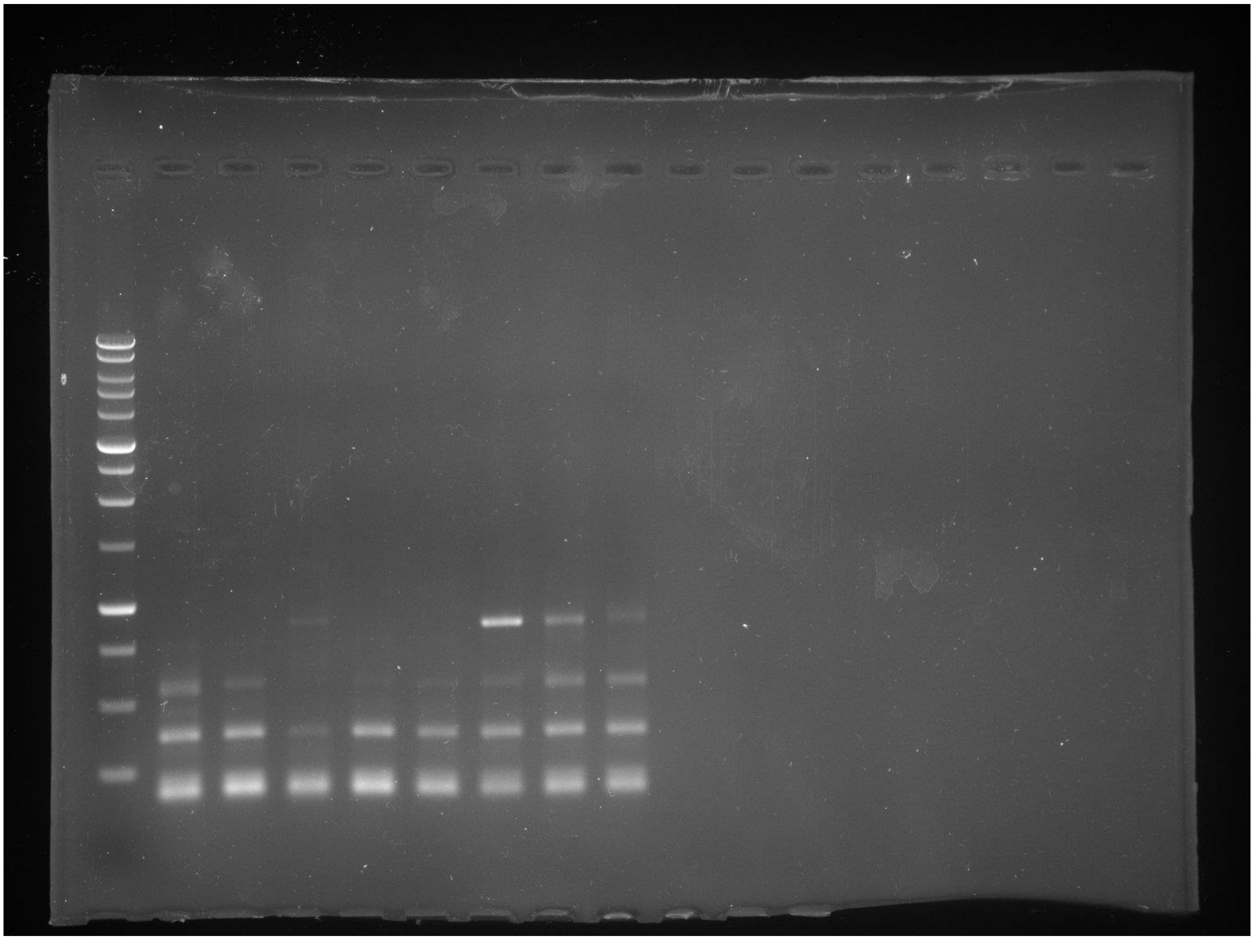

Fig. S4d – 2

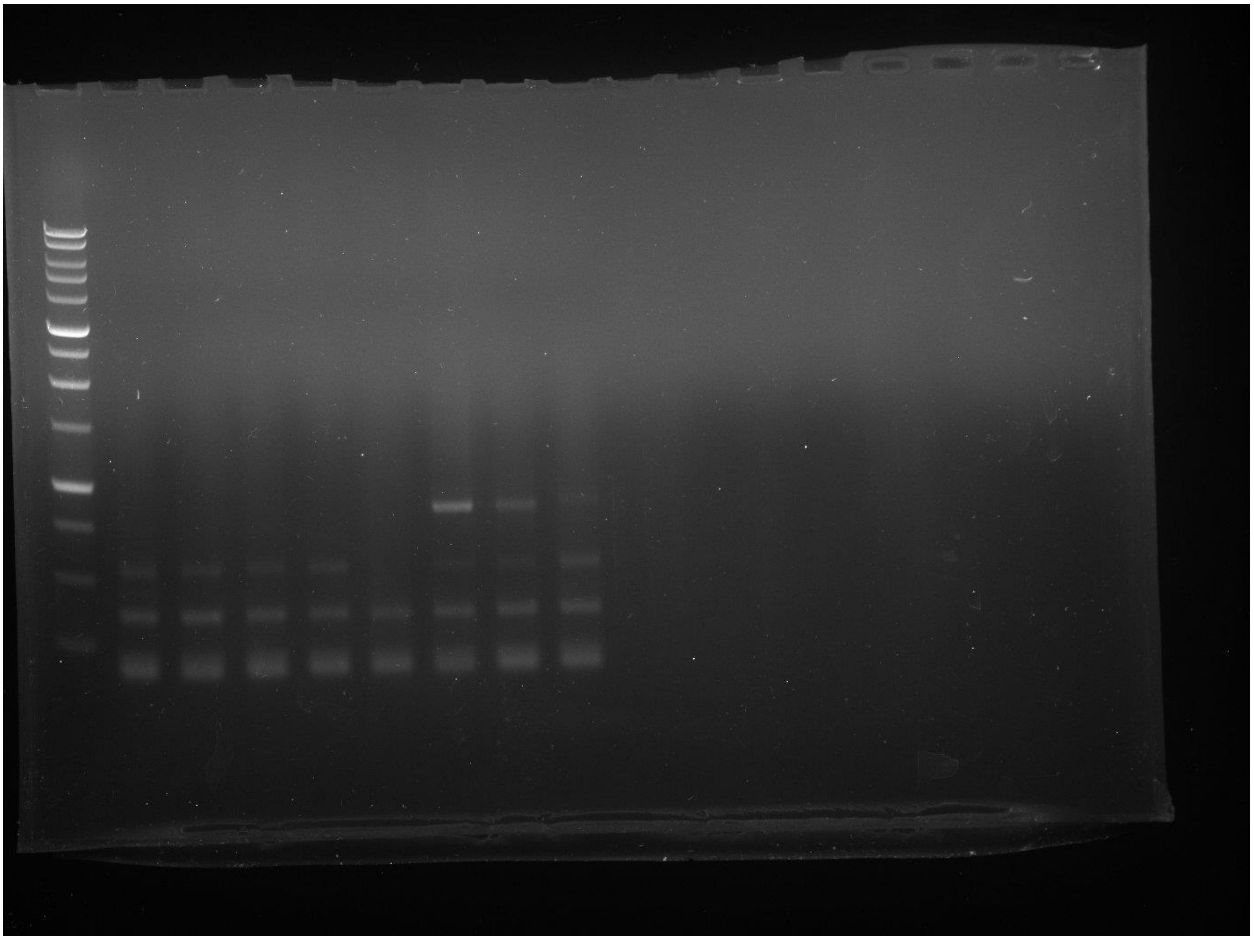

Fig. S4d – 3

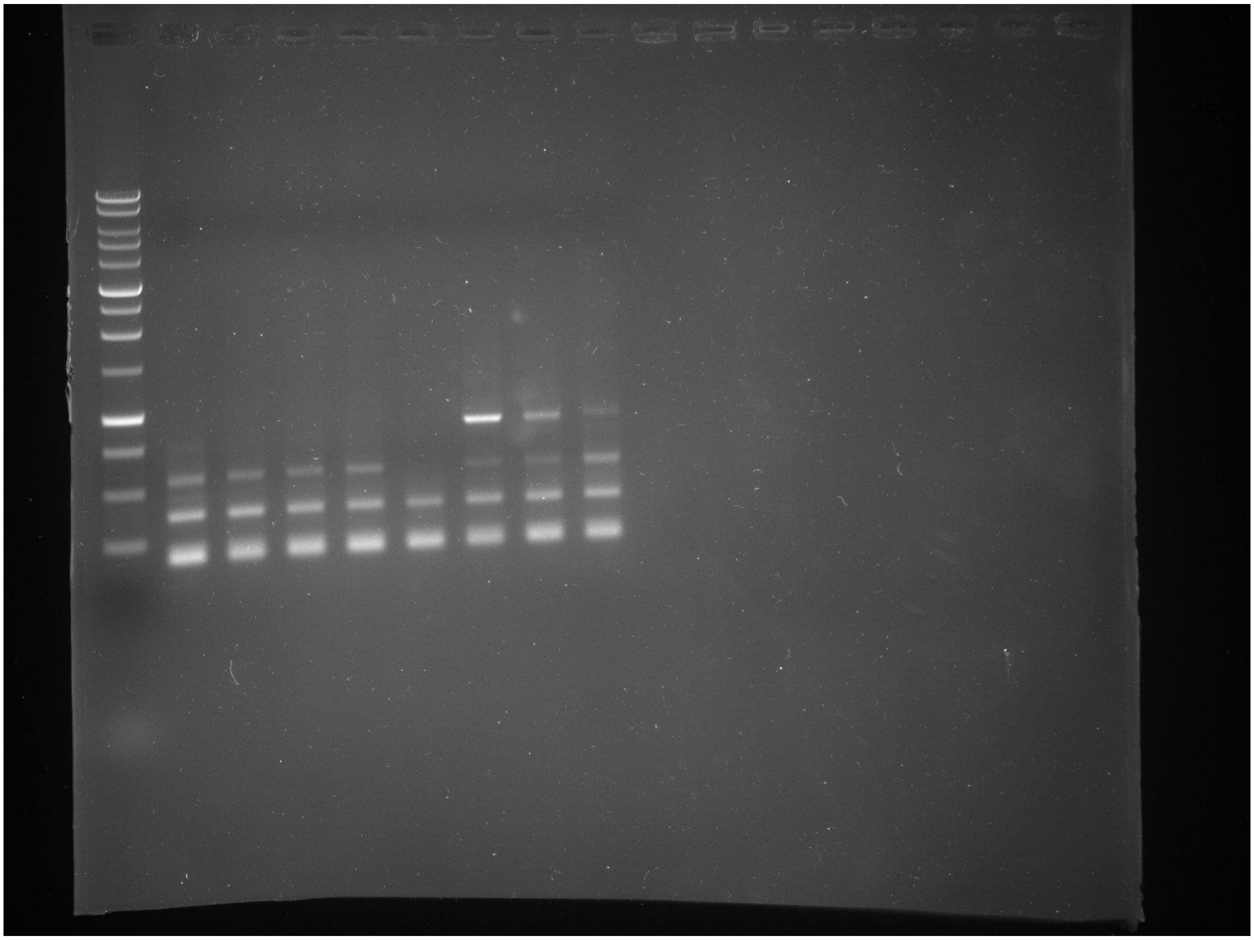

Fig. S8a – 1

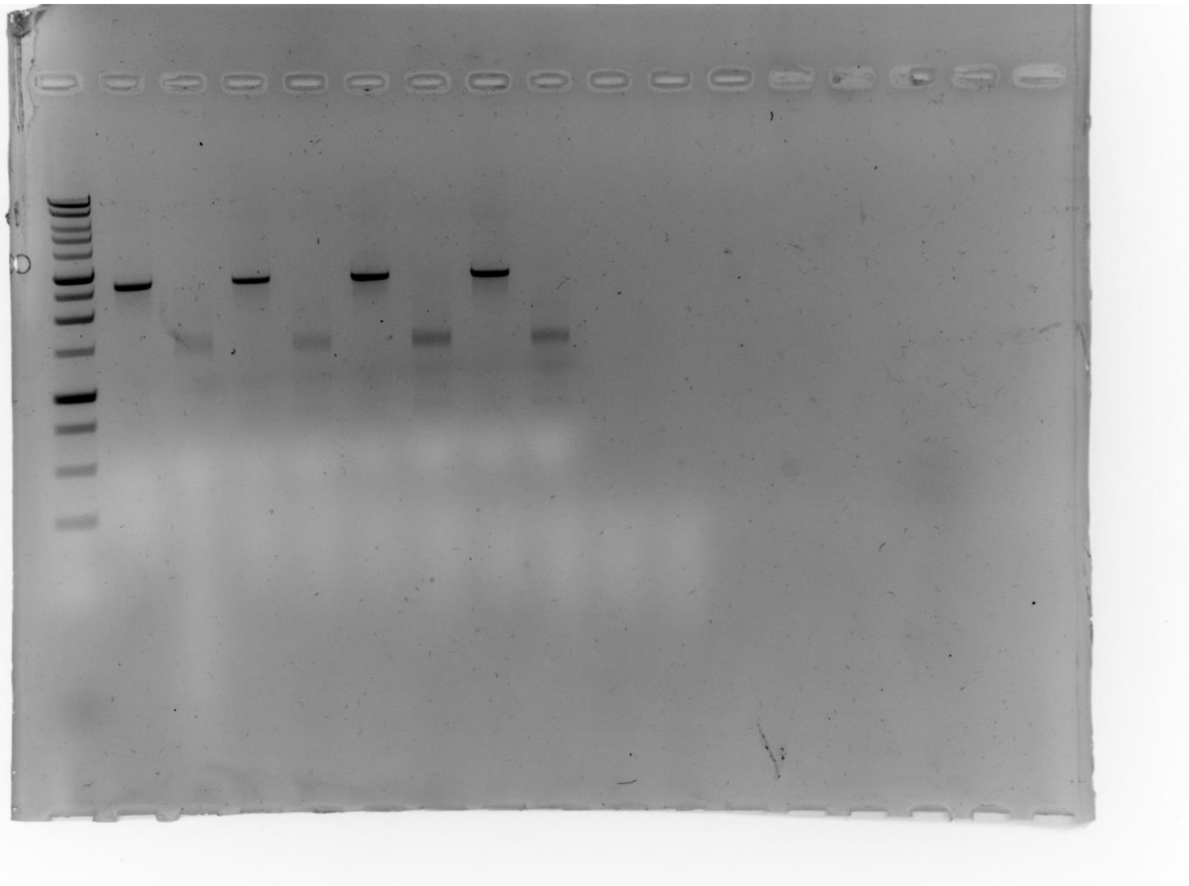

Fig. S8a – 2

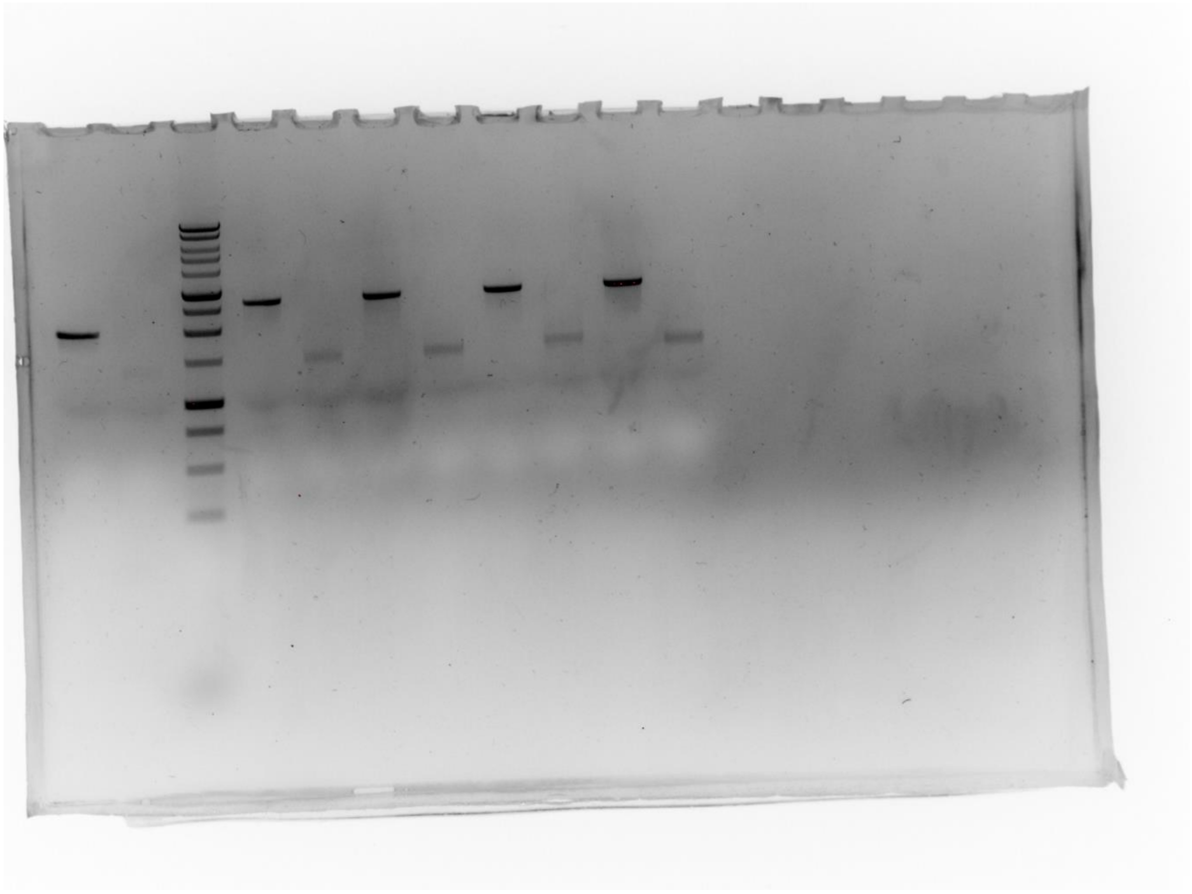

Fig. S10b

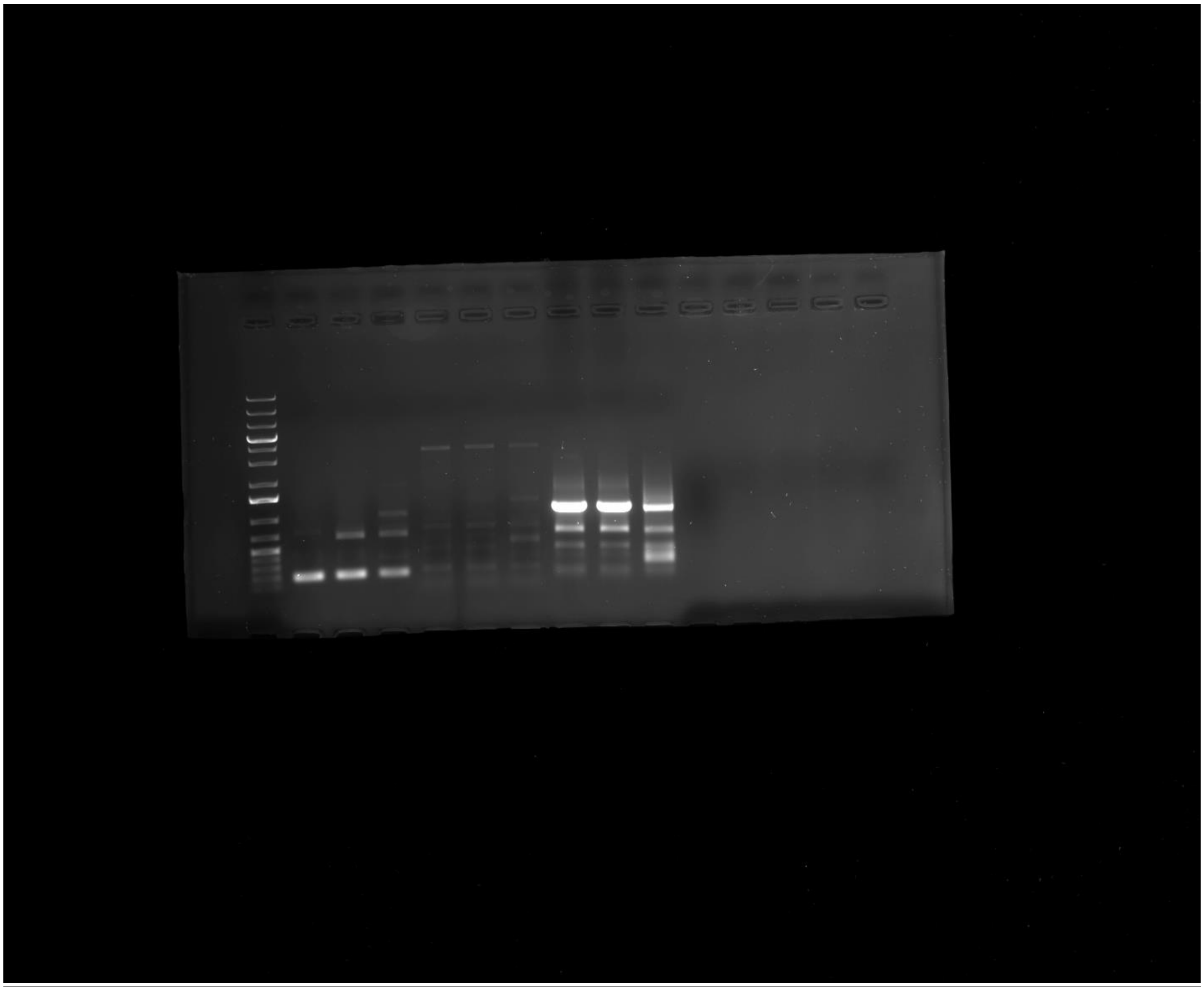

Fig. S10c – 1

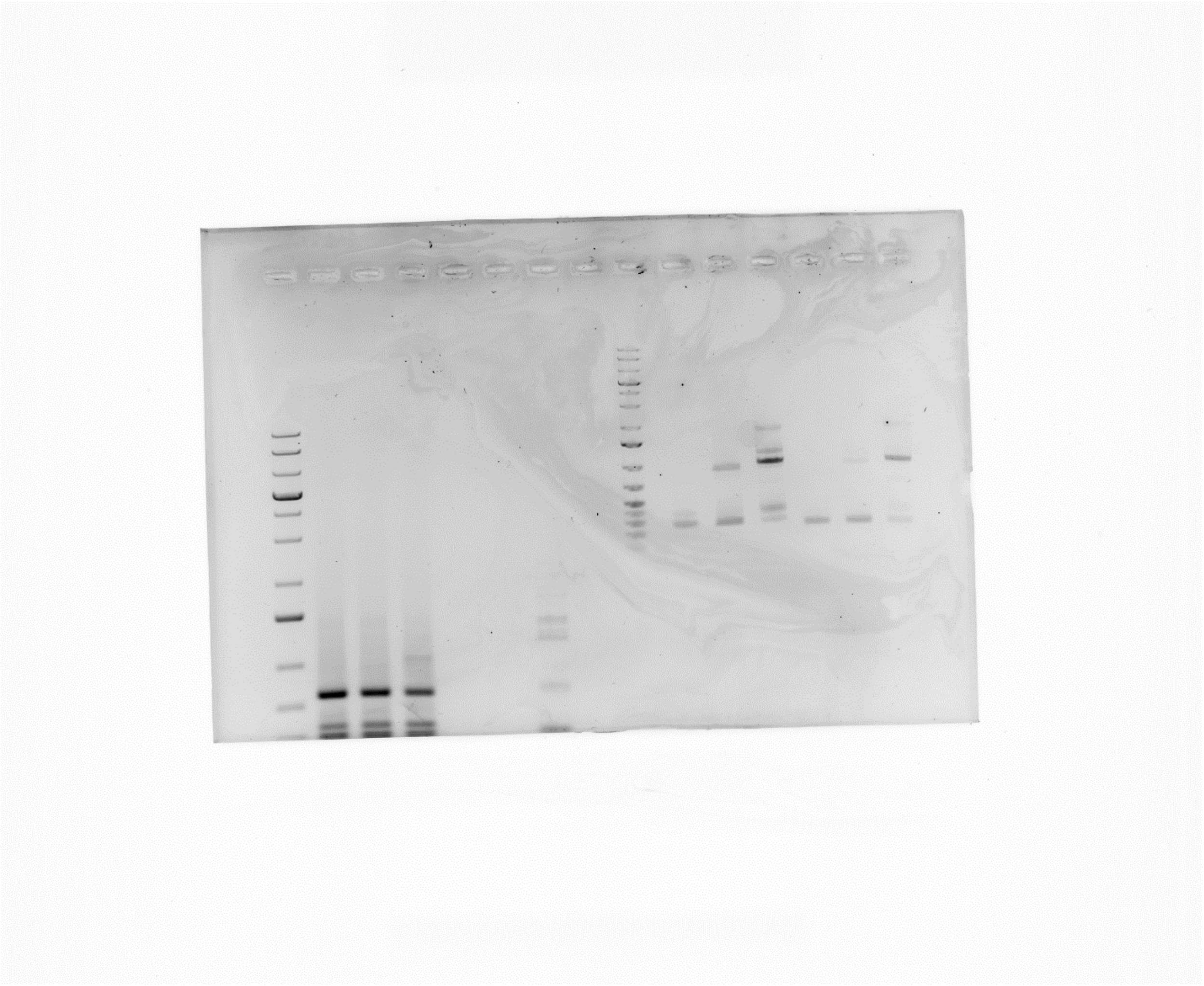

Fig. S10c – 2

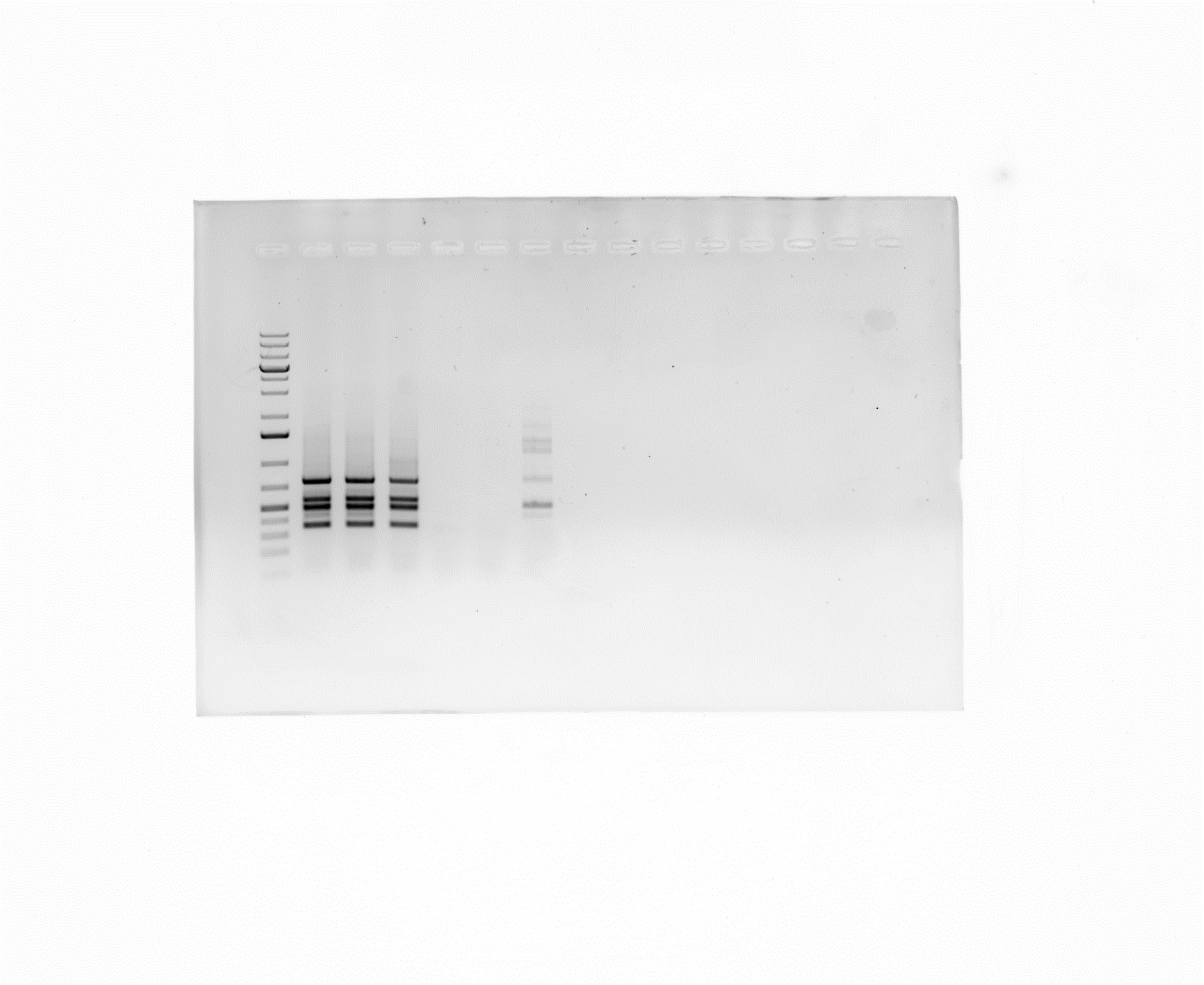

Fig. S10e

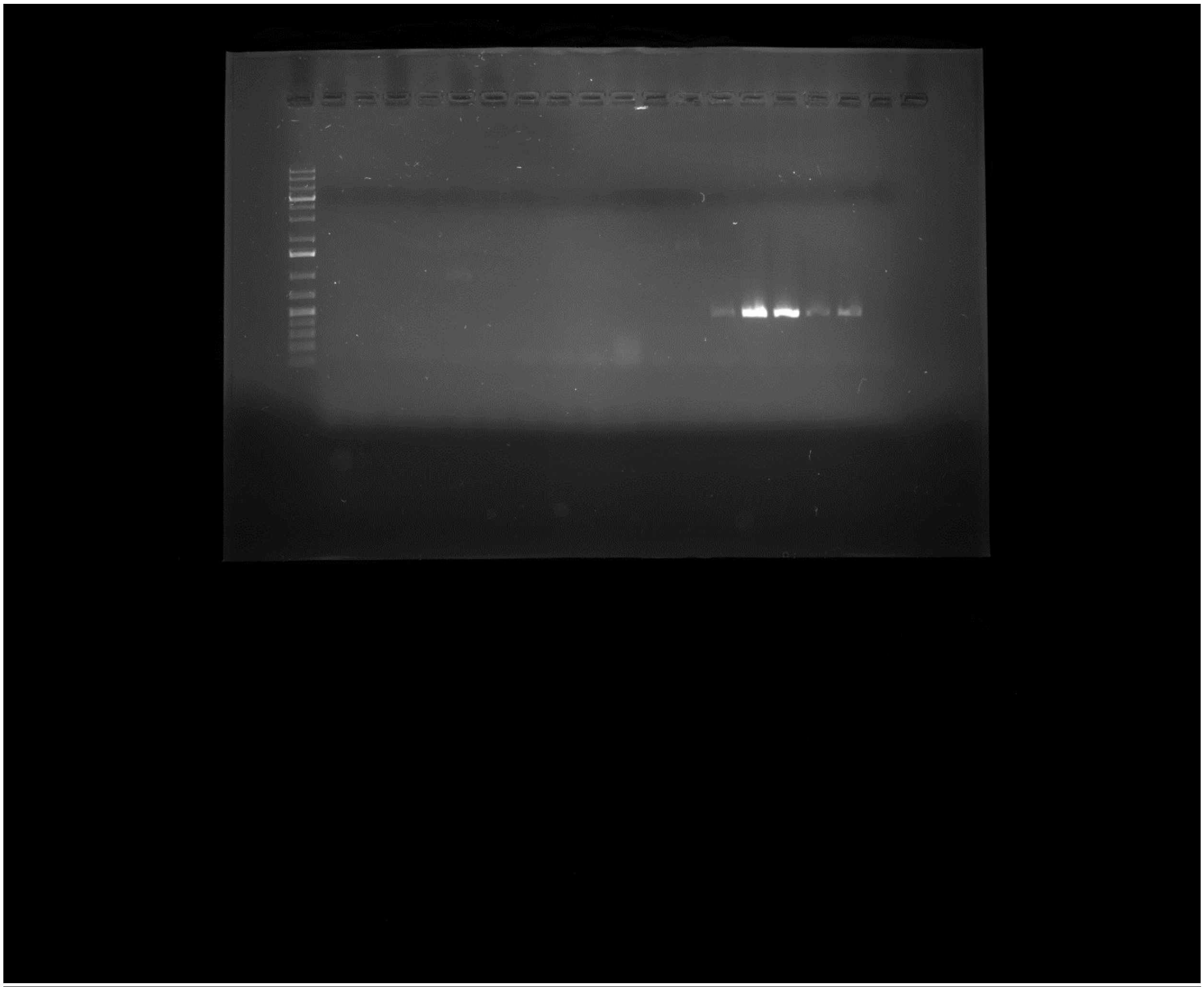

Fig. S11b – 1

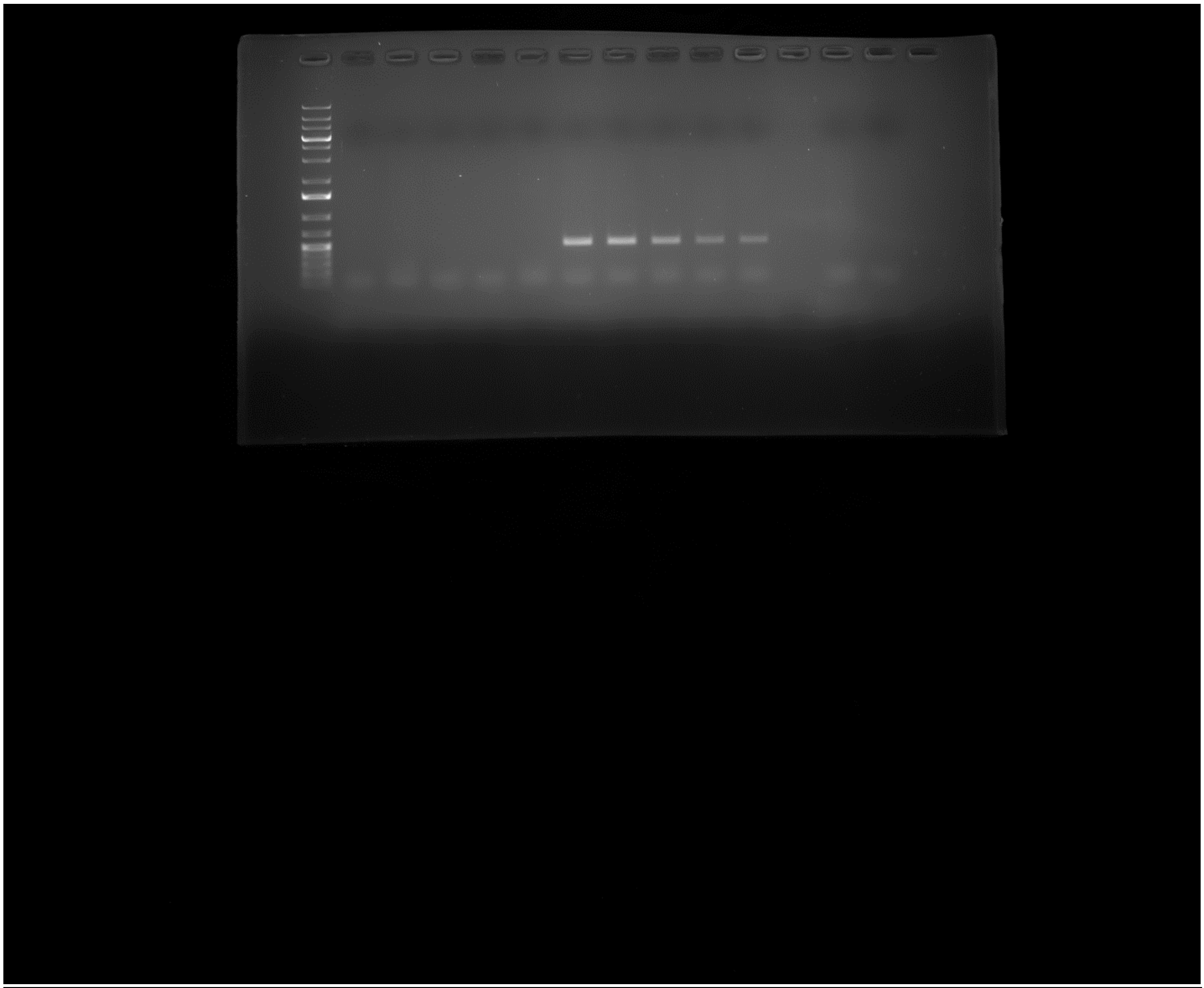

Fig. S11b – 2

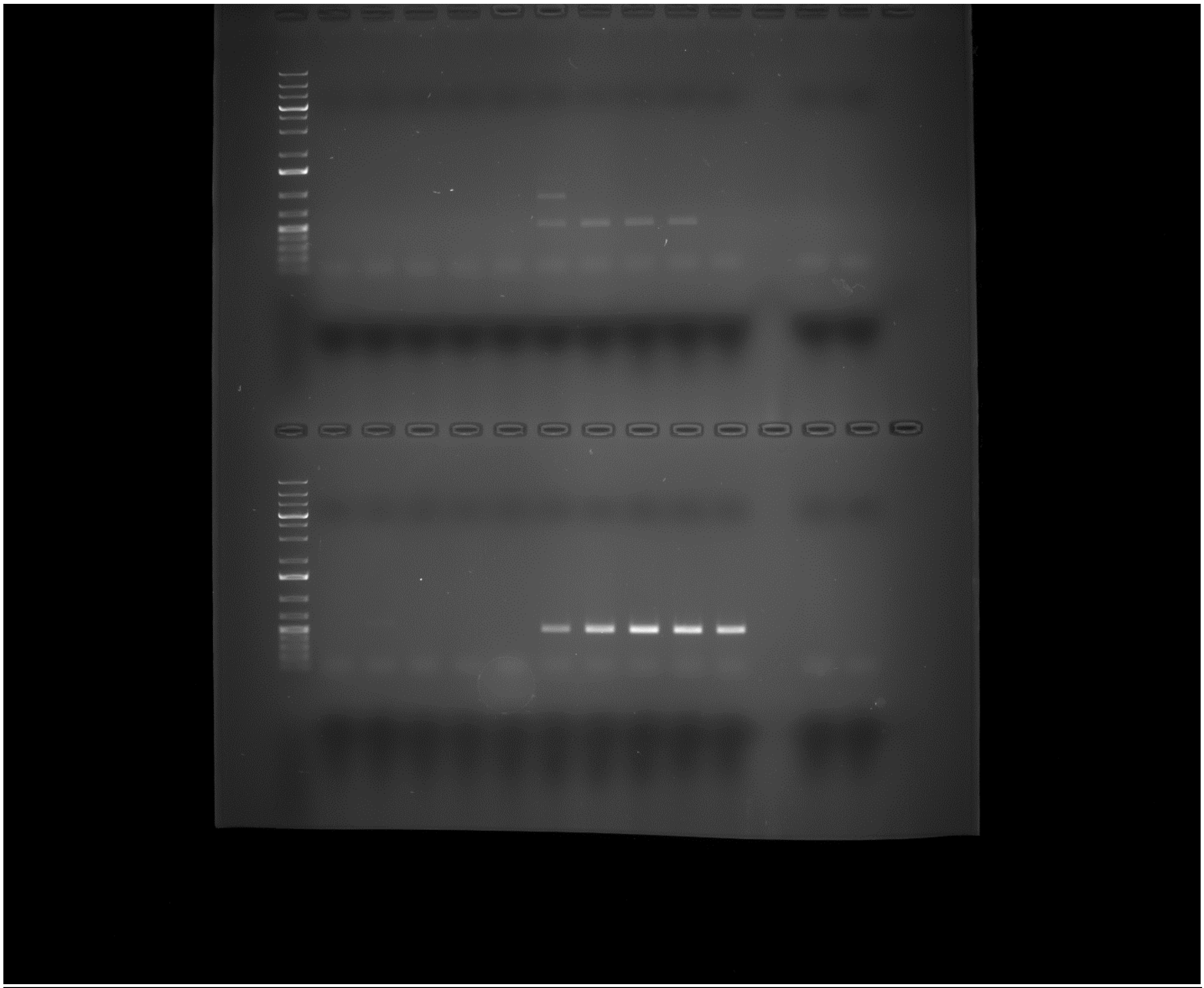

Fig. S11e

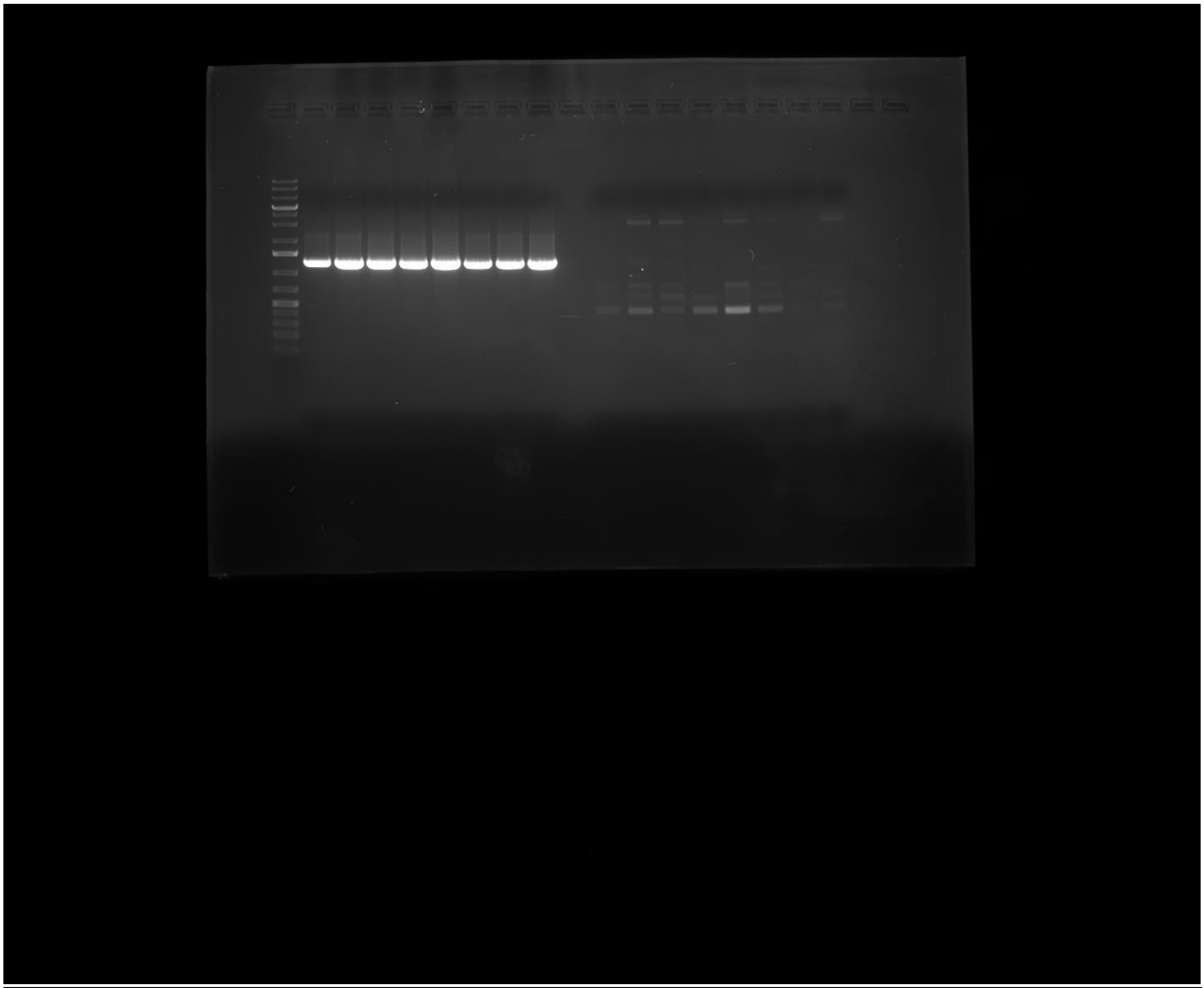

Fig. S11f

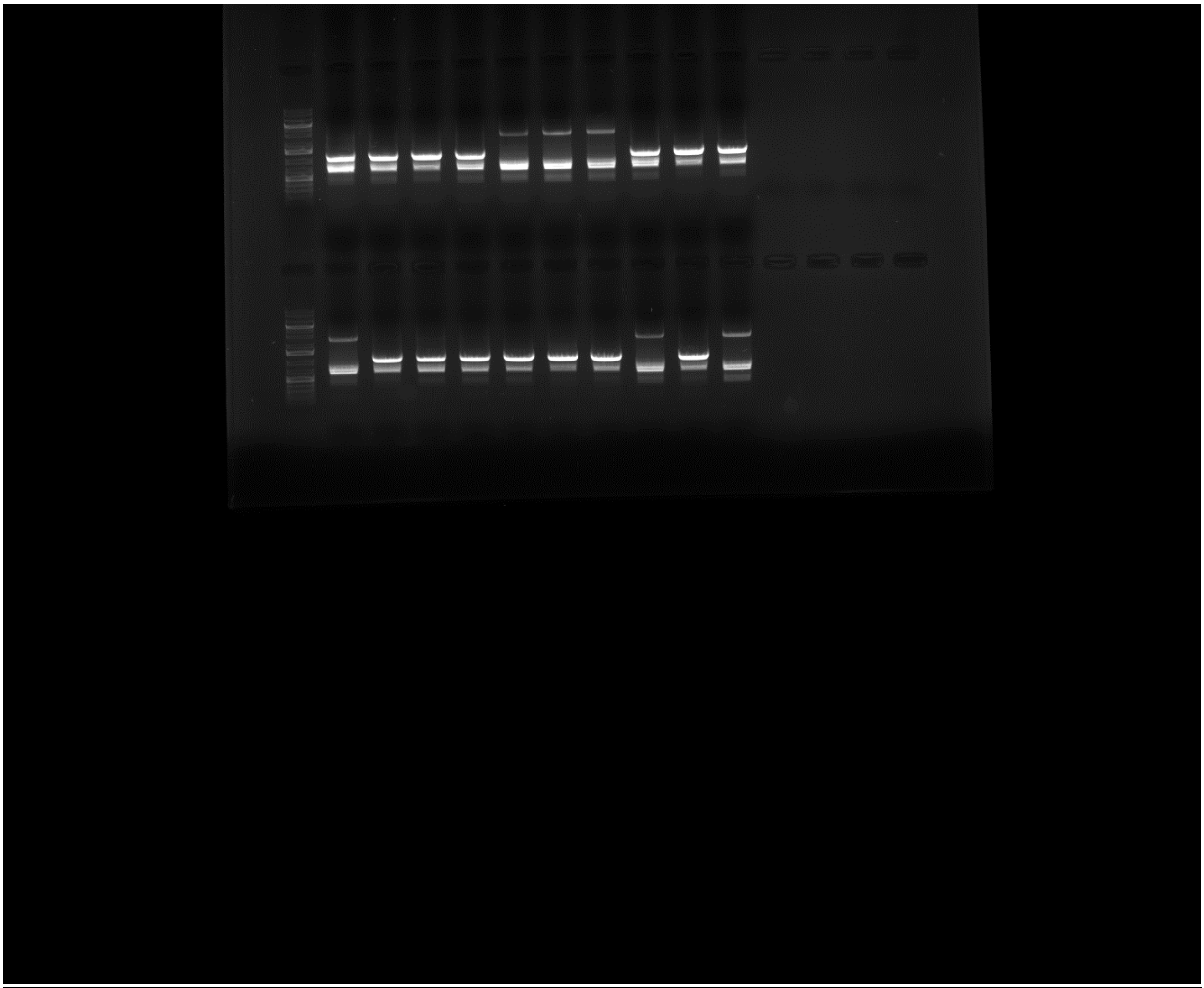

Fig. s14a

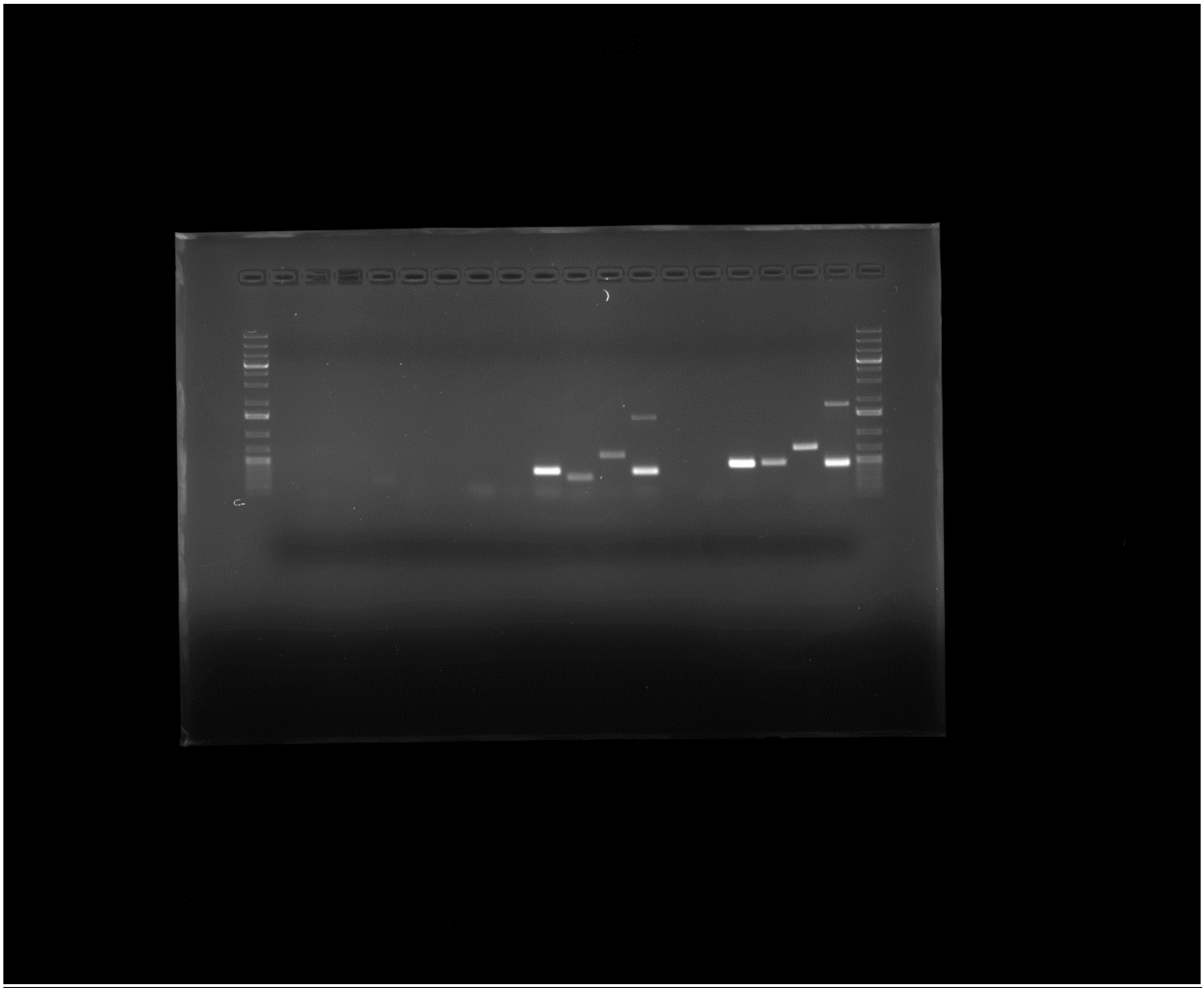

Supplement: Supplementary file 1 — Supplementary Figs. 1–17 (and raw uncropped source data). [file 41587_2025_2771_MOESM1_ESM.pdf]
