# Supplementary material for: Precise, predictable genome integrations by deep-learning-assisted design of microhomology-based templates
Source: Nat Biotechnol. 2025 Aug 12;44(6):1023–36. doi: 10.1038/s41587-025-02771-0 (PMC13271894; doi:10.1038/s41587-025-02771-0)

Precise, predictable genome integrations by deep learning–assisted design of microhomology-based templates

Uncropped blots and gels

Fig. 1g – 1

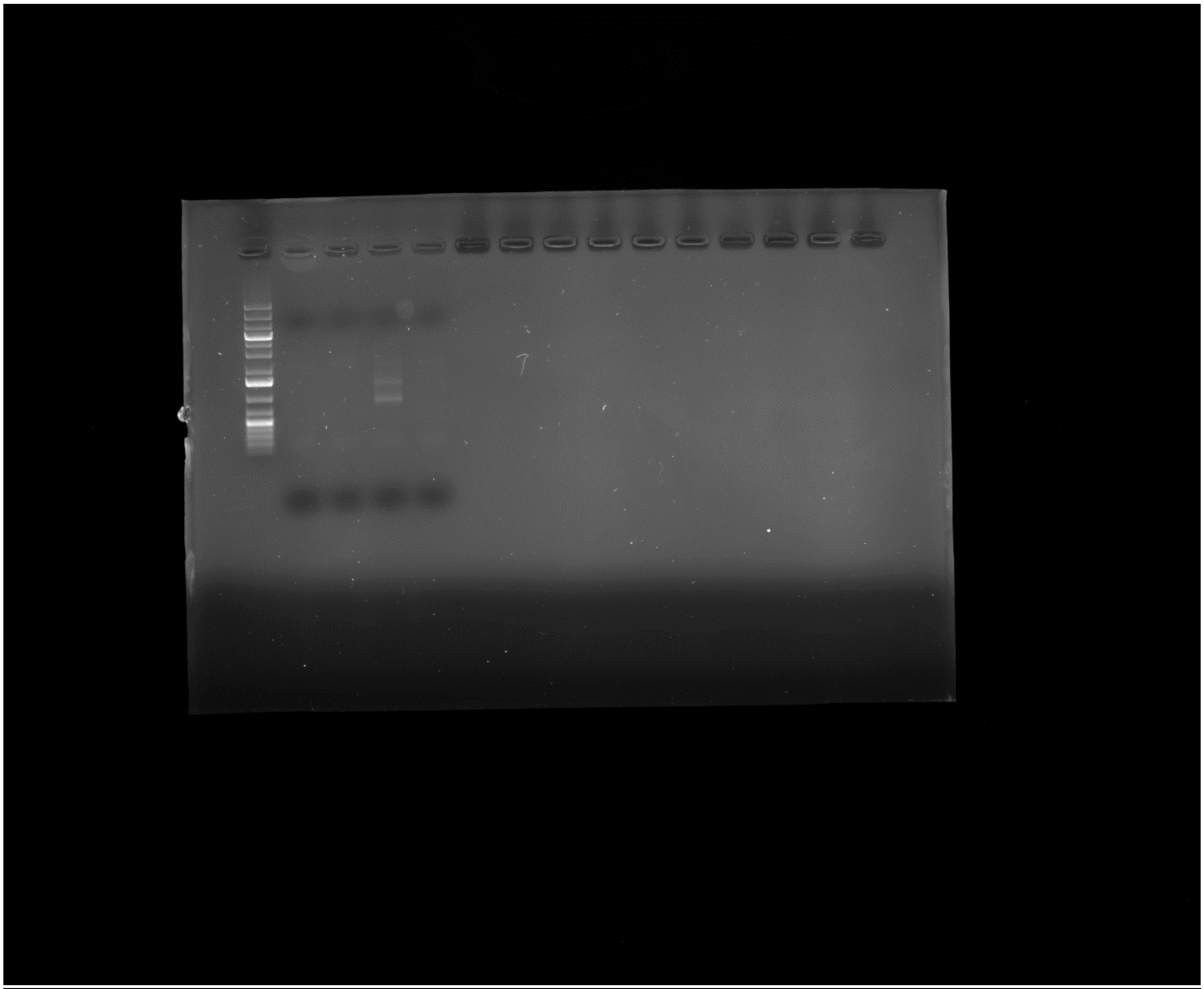

Fig. 1g - 2

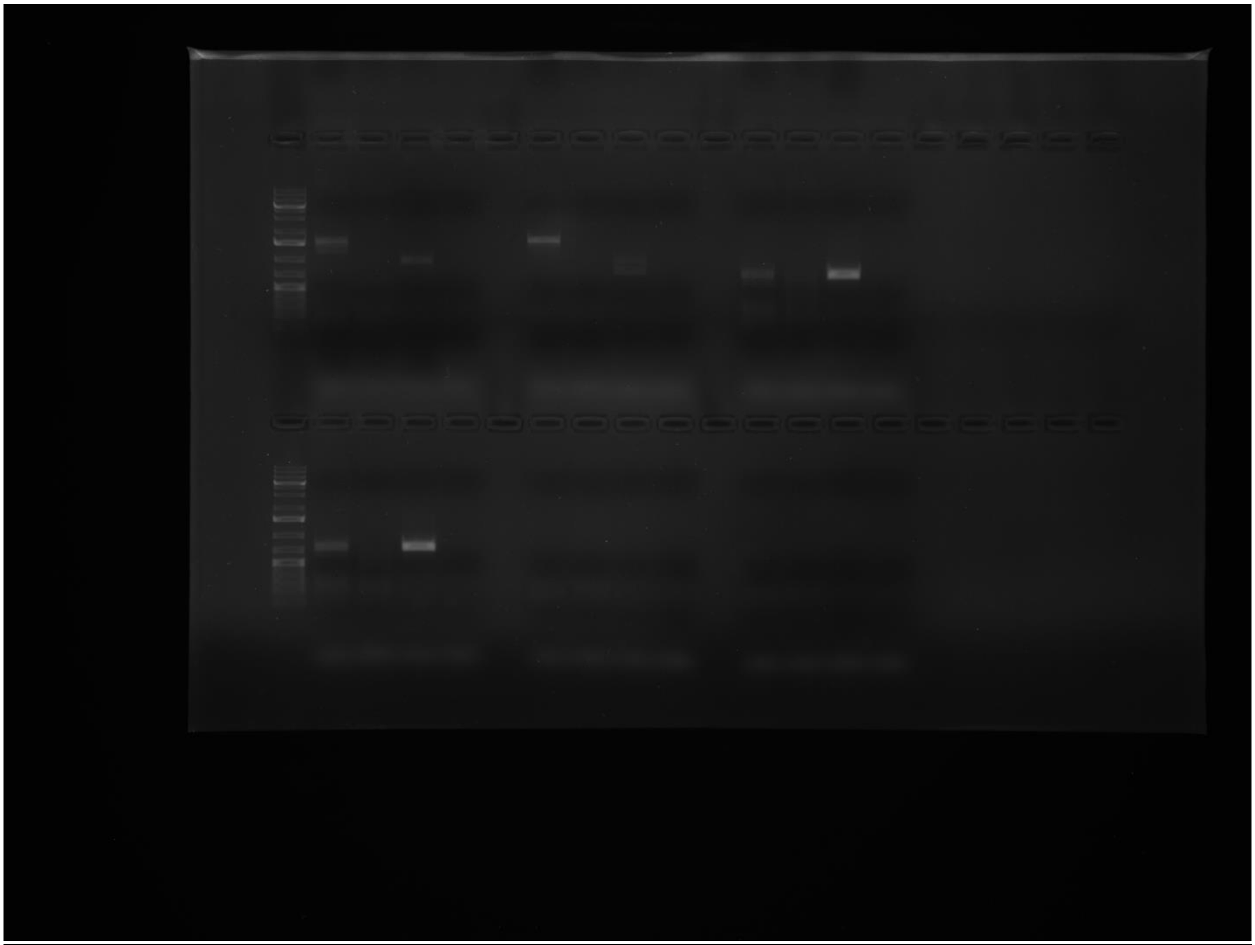

Fig. 3c-1

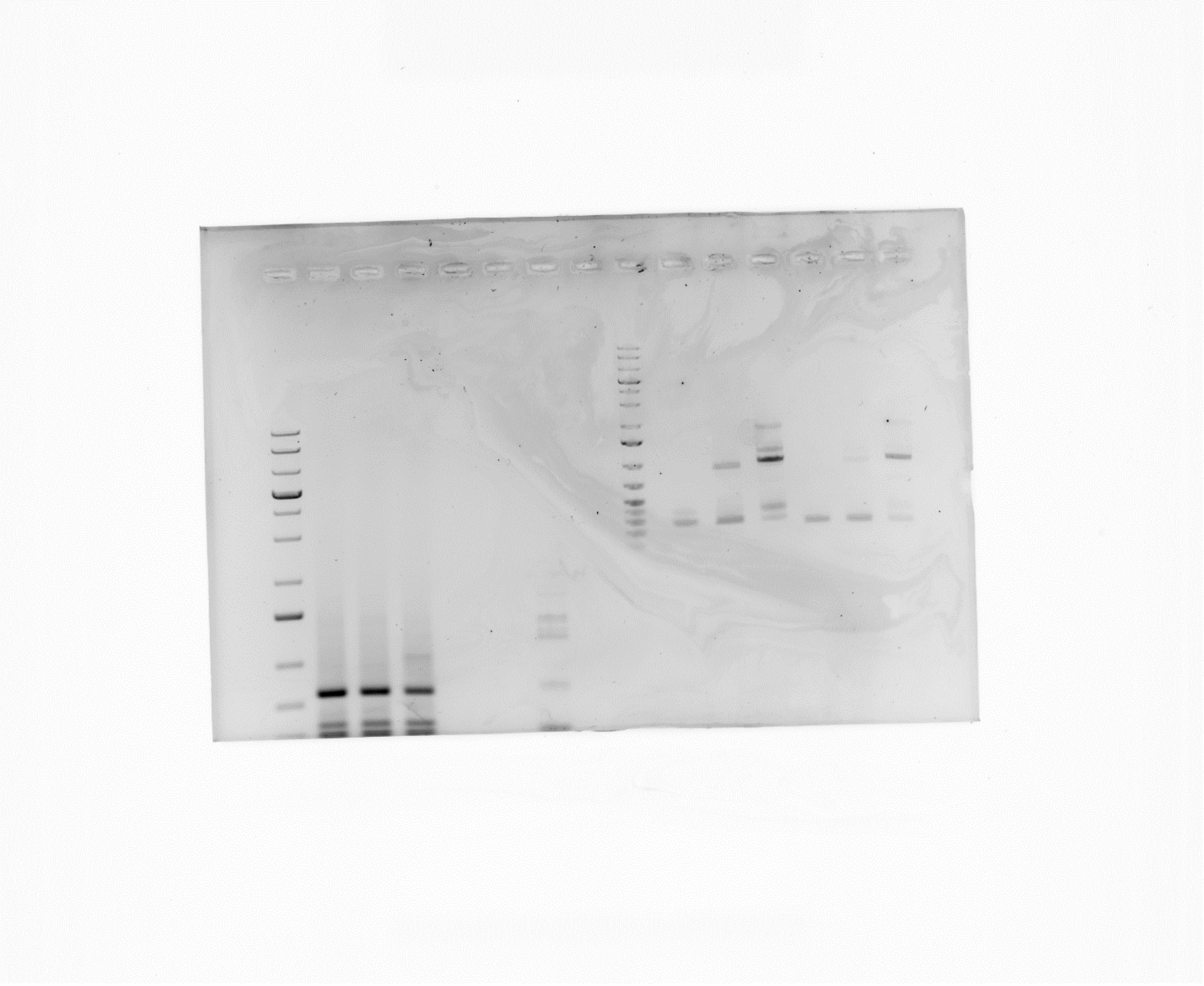

Fig. 3c-2

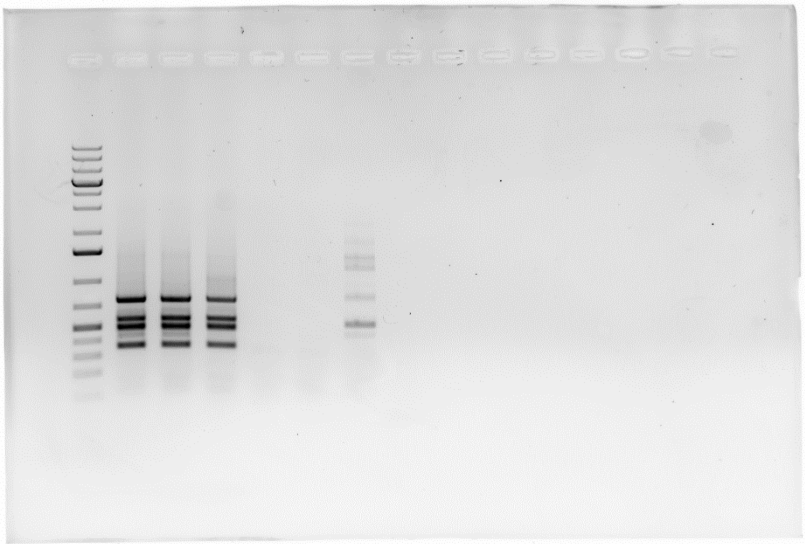

Fig. 3f-1

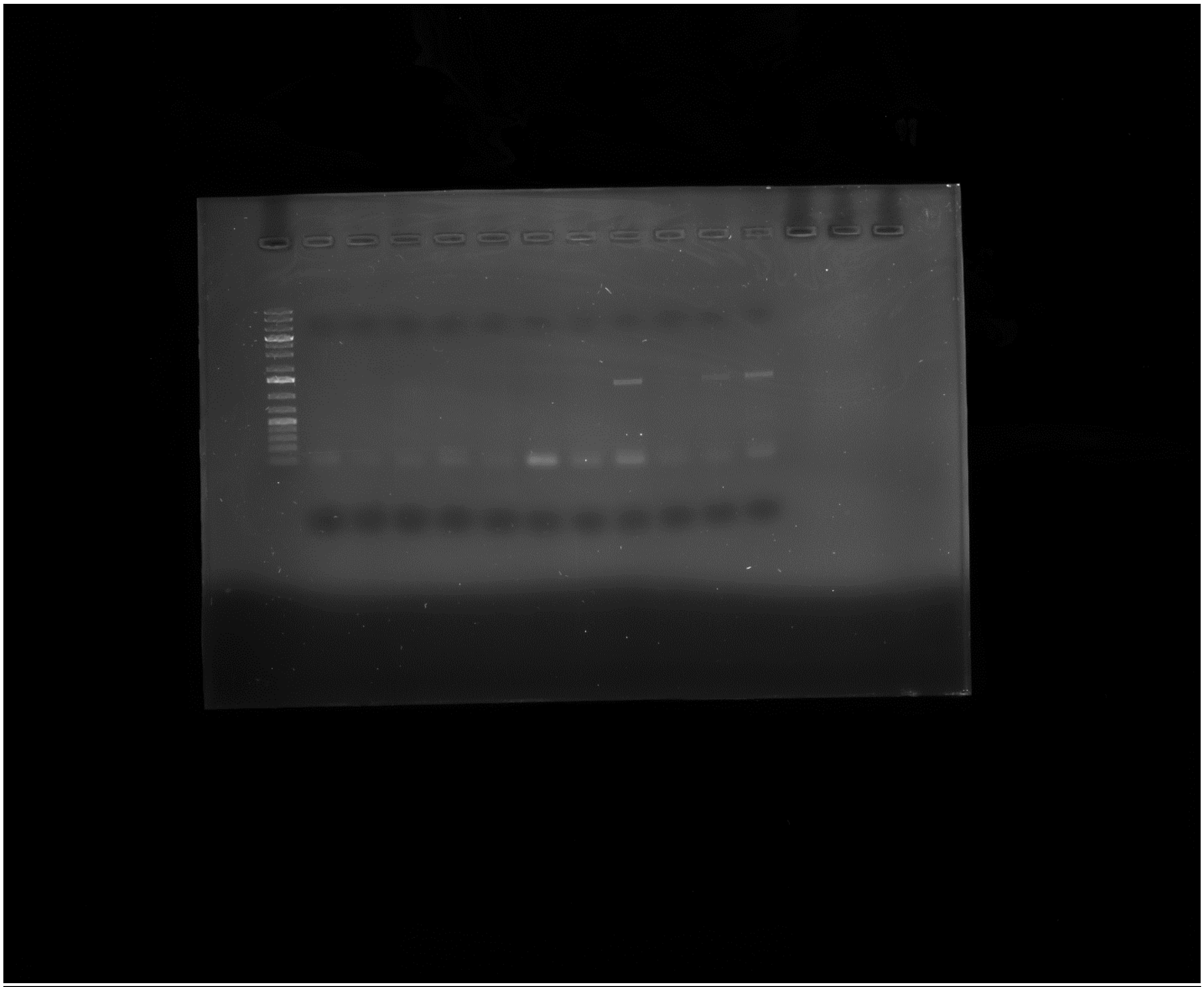

Fig. 3f-2

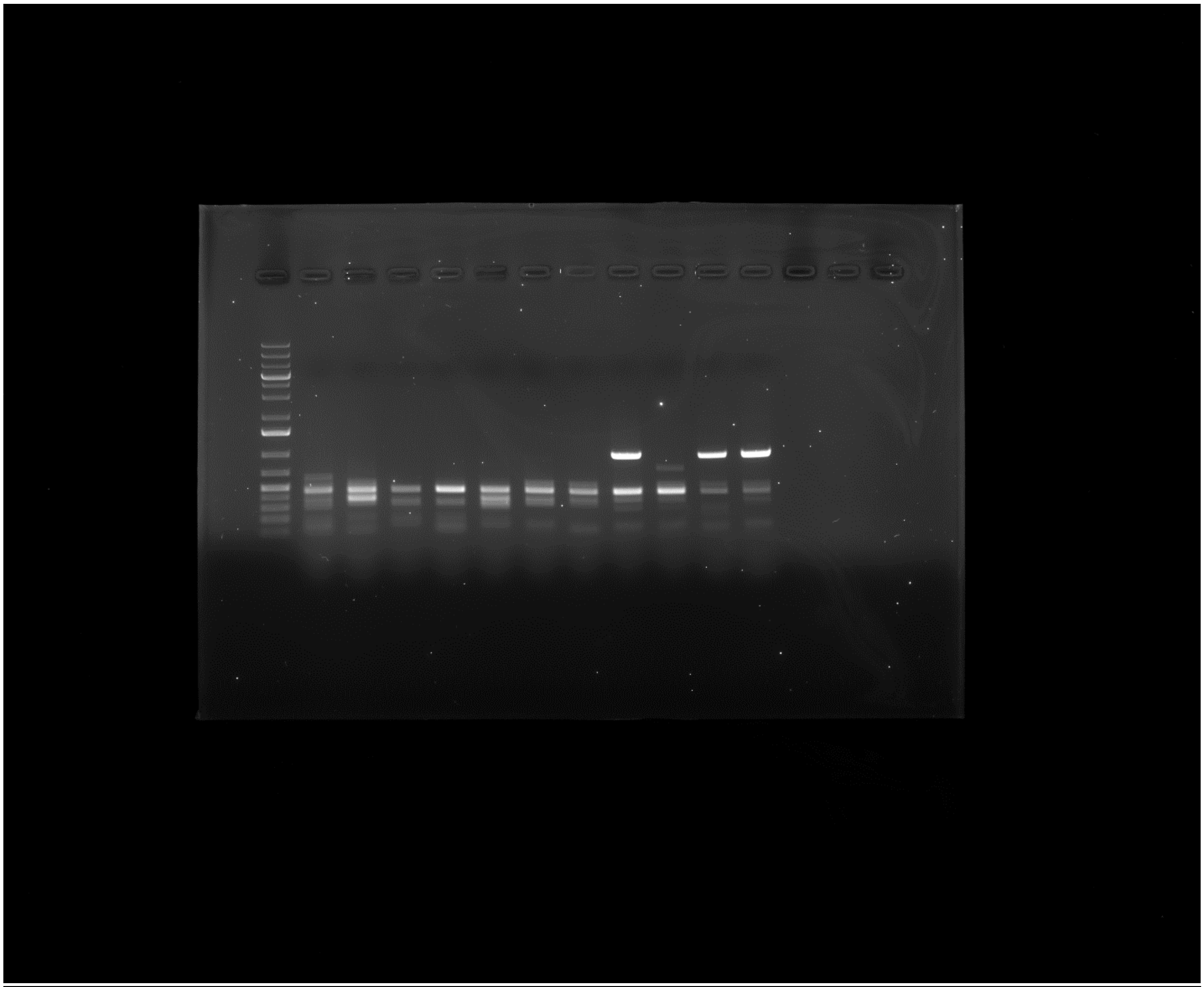

Fig. 4h - 1

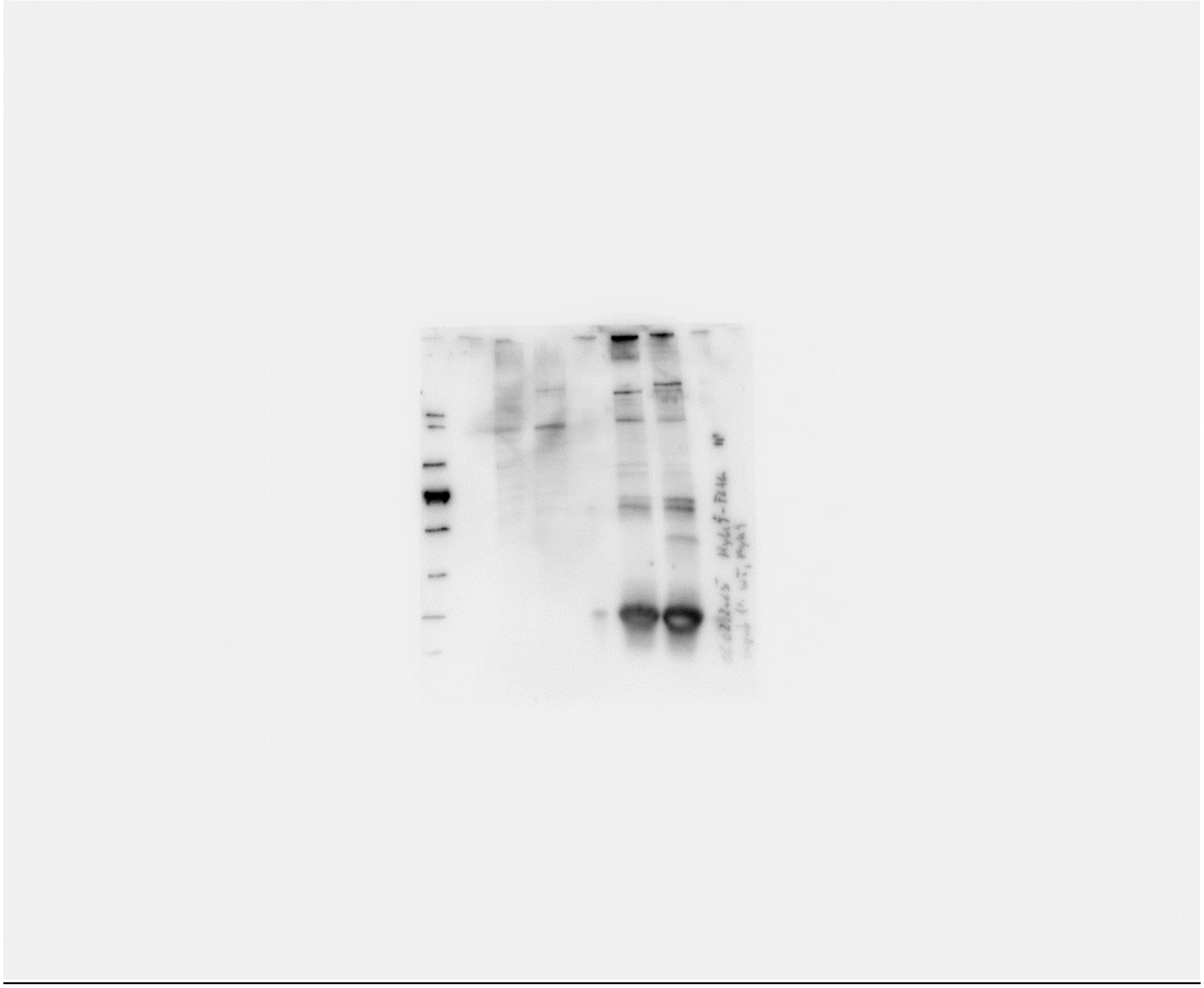

Fig. 4h-2

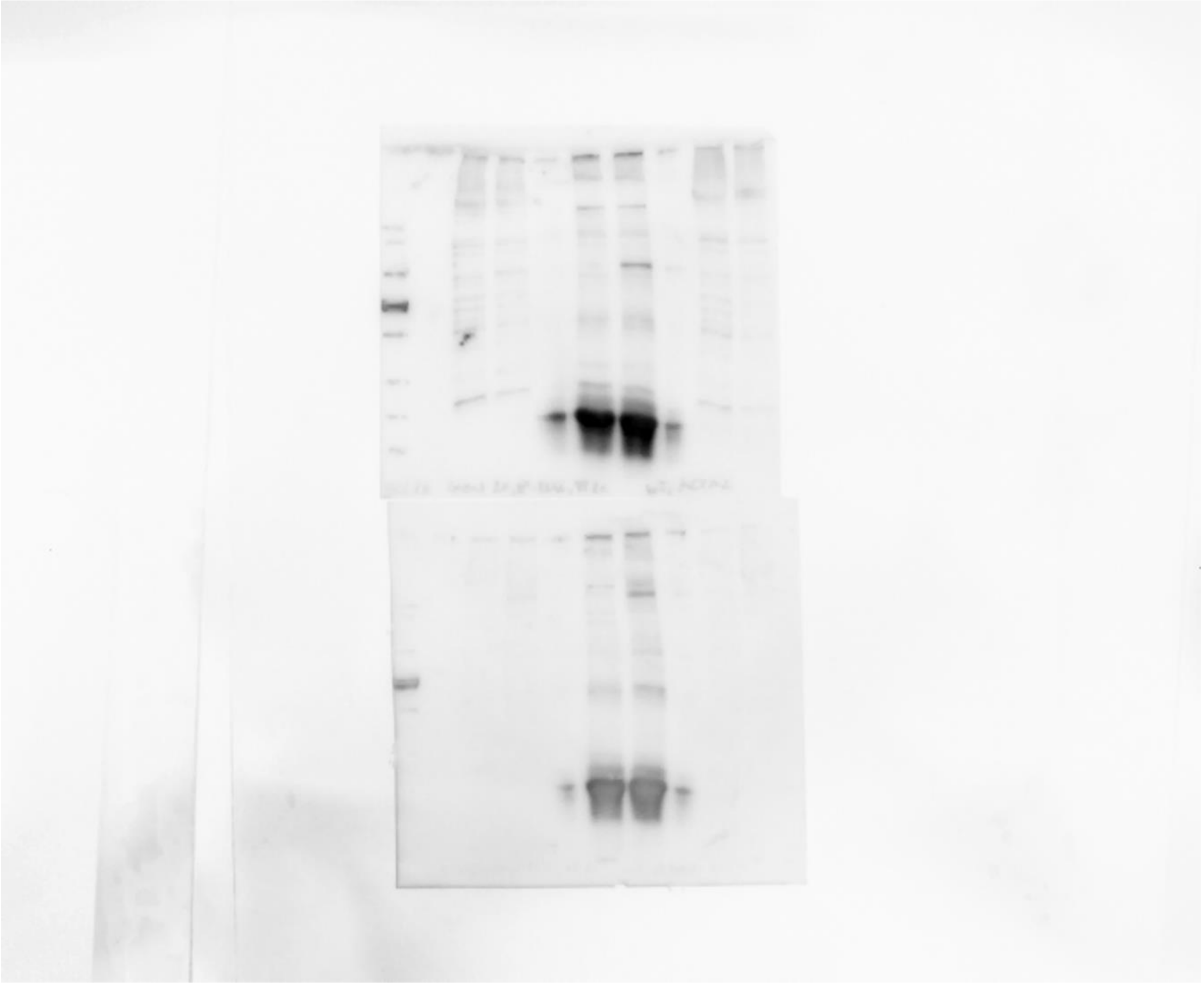

Fig. 5f

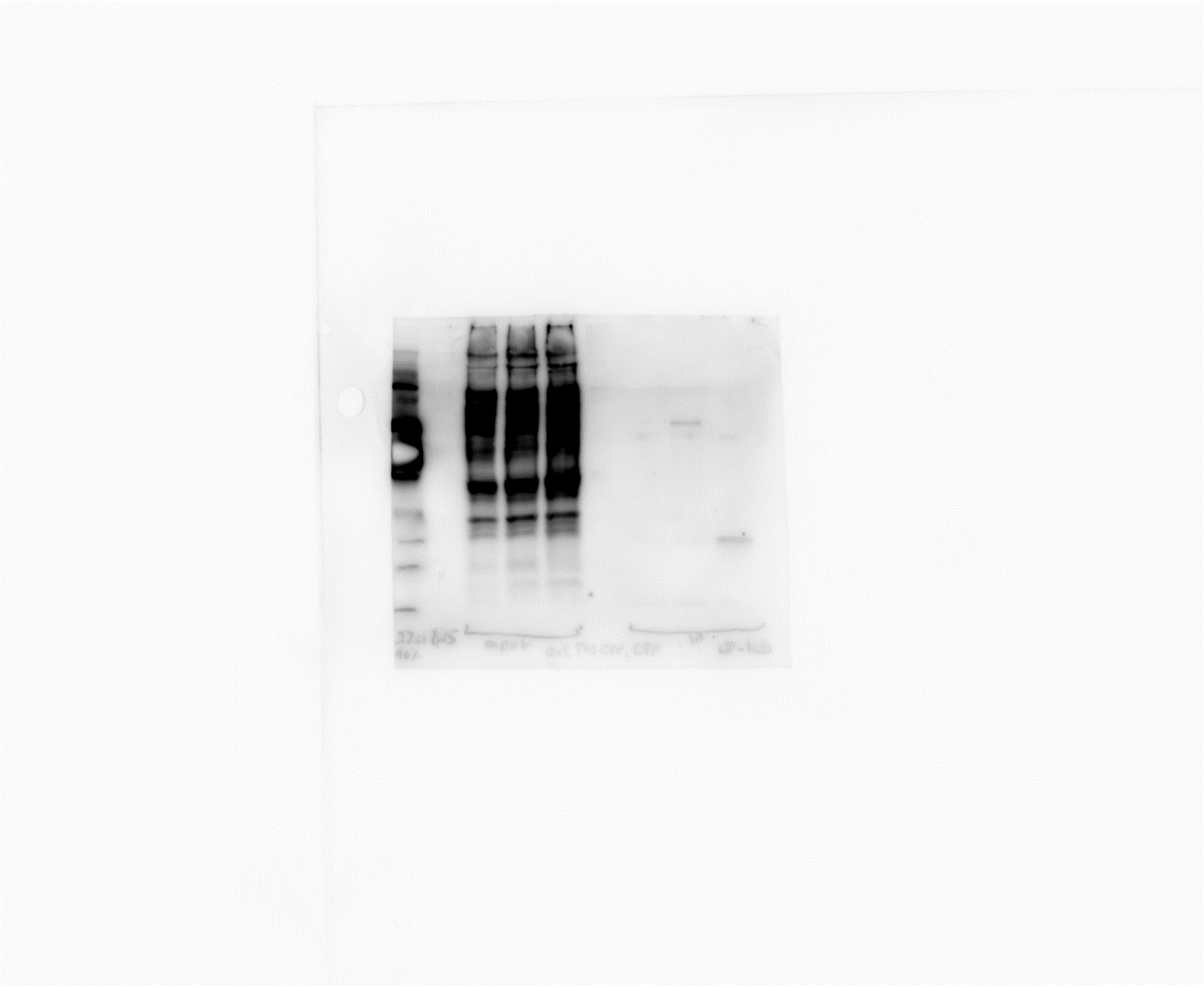

Fig. 6j

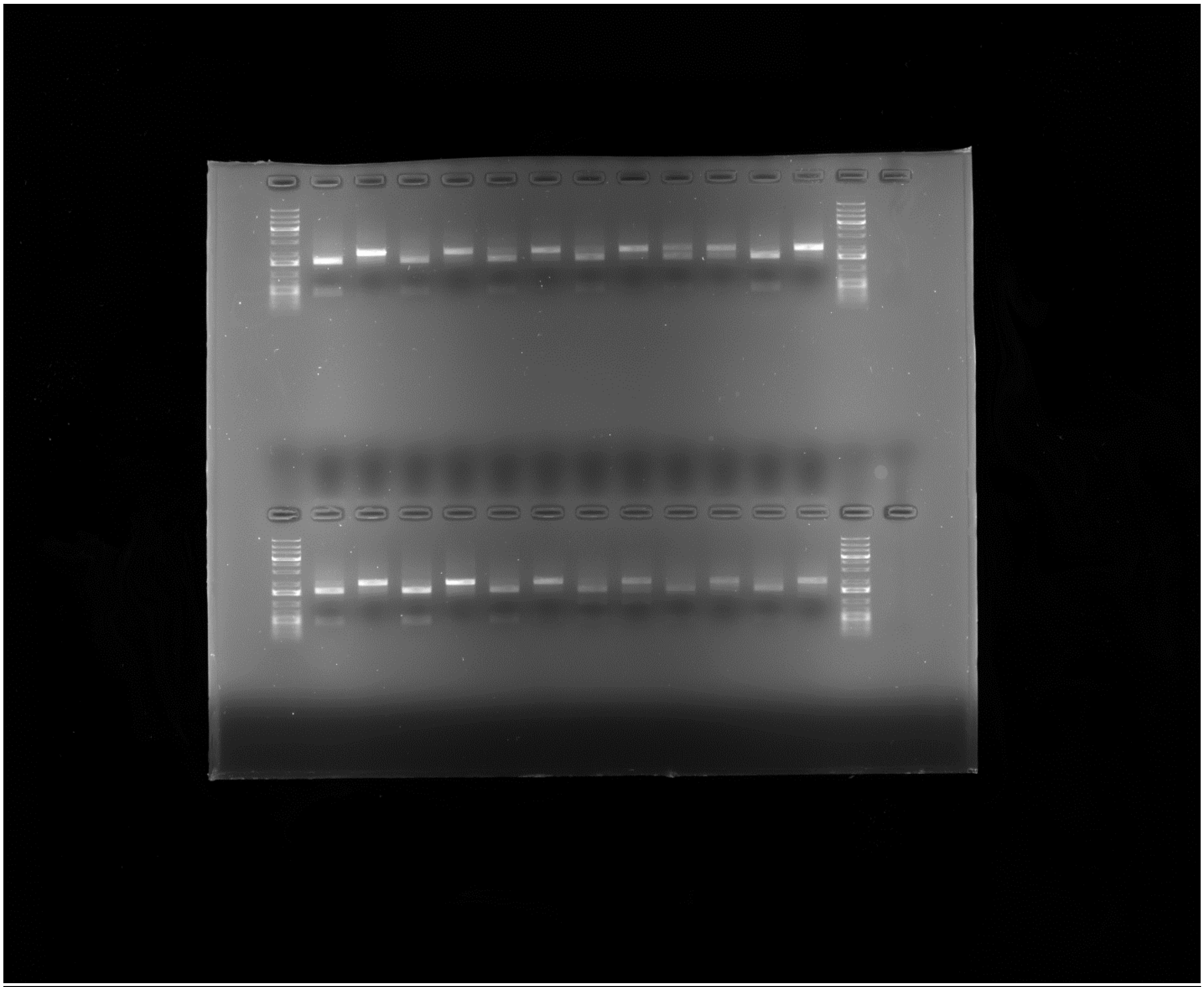

Supplement: Supplementary file 11 — Unprocessed western blots and gels. [file 41587_2025_2771_MOESM11_ESM.pdf]
